# Supplementary material for: Siglec-6 as a therapeutic target for cell migration and adhesion in chronic lymphocytic leukemia
Source: Nat Commun. 2024 Jun 18;15:5180. doi: 10.1038/s41467-024-48678-3 (PMC11189495; doi:10.1038/s41467-024-48678-3)
Supplement: Supplementary file 1 — Supplementary Information [file 41467_2024_48678_MOESM1_ESM.pdf]

Supplementary information

Contents

Supplementary Methods

Supplementary Fig. 1: JML-1 is a blocking antibody specific to Siglec-6.

Supplementary Fig. 2. Siglec-6 promotes migration of B-CLL cells.

Supplementary Fig. 3. Siglec-6 expression on different subsets of CLL cells.

Supplementary Fig. 4. Siglec-6 and DOCK8 are both essential for *in vitro* migration of MEC1-002 cells.

Supplementary Fig. 5. Siglec-6 is essential for membrane localization of DOCK8.

Supplementary Fig. 6. sTn mediated Siglec-6 and DOCK8 dependent Cdc42 activation is independent of SHP-2.

Supplementary Fig. 7. s Tn mediated Siglec-6 and DOCK8 dependent actin polymerization is independent of SHP-2.

Supplementary Fig. 8. Siglec-6 mAb inhibits *in vitro* migration of Siglec-6 positive MEC1-002 cells to mouse BMSCs and Siglec-6 targeted T-biAb improves overall survival of mice with hSiglec-6×TCL1 leukemia.

Supplementary Table 1. Siglec-6 associates with DOCK8, a guanine nucleotide exchange factor.

Supplementary Table 2. List of flow cytometry antibodies.

Supplementary Table 3. List of western blotting, immunoprecipitation and immunofluorescence antibodies.

Supplementary Table 4. List of proteins identified from mass spectrometry analysis of Siglec-6 interacting partners.

Supplementary Methods

Human samples and study approval: Peripheral blood mononuclear cells (PBMCs) and bone marrow mesenchymal stromal cells (BMSCs) were collected from normal healthy donors or CLL patients in compliance with the Declaration of Helsinki. All subjects gave written and informed consent for the blood products to be used for research according to an IRB approved protocol. Blood from CLL patients was collected at The Ohio State University Comprehensive Cancer Center (Columbus, OH, USA). Normal healthy donor PBMCs were obtained from Red Cross partial leukocyte preparations. PBMCs were isolated using Ficoll density-gradient centrifugation, Ficoll-Paque Plus (GE Healthcare). B cells from CLL patients were isolated using B-cell RosetteSep enrichment kit (#15064, StemCell Technologies) according to manufacturer's protocol. CLL-BMSCs were cultured from bone marrow aspirates from CLL patients. Normal healthy donor-BMSCs were isolated from spongiform bone marrow of hip bone samples stored in the Leukemia Tissue Bank (LTB) at OSU.

Animal studies: The human Siglec-6 transgenic mouse (hSiglec-6-Tg) was generated on a C57BL/6 background (Jackson laboratory, 000664) at the OSUCCC Transgenic Mouse Facility by conventional methodology[1]. B-cell restricted hSiglec-6 expression is driven by immunoglobulin (Ig) V_H promoter and Ig_H-μ enhancer elements in the pBH expression vector[1].The transgenic construct was generated by ligating the cDNA sequence of human Siglec-6 into BglII sites within the pBH vector via blunt end ligation. To generate our Siglec-6+ leukemia model, we crossed hSiglec-6-Tg mice with Eμ-TCL1 mice (C57BL/6 background)[2,3]. Mice were housed in microisolator cages under controlled temperature and humidity. All animal procedures were performed in accordance with Federal and Institutional Animal Care and Use Committee (IACUC) requirements. Two founder lines were established and presence of the transgene in founder lines

was determined by PCR. Primer sequences to detect *SIGLEC6* gene are as follows: Forward primer 5'-TTTGTGCATTGGAAACCAGA-3' and reverse primer 5'-CTGTCTGGAACTGGTGCTGA-3'. Humanized CD3ε mice were kindly provided by Dr. Meixiao Long (The Ohio State University). To generate them, human CD3 transgenic mice were obtained from Jackson Laboratory (Stock No: 020456, B6.Cg-Tg (CD3E) 600Cpt/J. Wild-type C57/BL6 mice were bred with these mice, and mice with peripheral T cell numbers and CD4:CD8 ratios closely resembling those of wild-type littermates were chosen for subsequent breeding. 12–16-week mice that were heterozygous for the human CD3 transgene were used for subsequent studies.

Mass spectrometry: Beads were washed twice with 50 mM ammonium bicarbonate (25-50 uL each time depending on beads volume). Each time, the supernatant was kept and pooled. After the second wash, 5 uL of DTT (5 ug/uL in 50 mM ammonium bicarbonate) was added and the sample was incubated at 56ºC for 15 min. After the incubation, 5 uL of iodoacetamide (15 mg/ml in 50 mM ammonium bicarbonate) was added and the sample was kept in dark at room temperature for 30 min. Either 250 ng or 500 ng of sequencing grade-modified trypsin (Promega) prepared in 50 mM ammonium bicarbonate was added to the sample reaction was carried on at 37ºC for overnight, additional 50 mM ammonium bicarbonate was added to make the final volume of the samples to 100 uL. The reaction was quenched the next morning by adding 0.1% acetic acid for acidification. Supernatant was taken out, concentrated for LC/MSMS analysis. Nano-liquid chromatography-nanospray tandem mass spectrometry (Nano-LC/MS/MS) of protein identification was performed on a Thermo Scientific Orbitrap Fusion mass spectrometer equipped with an EASY-Spray™ Sources operated in positive ion mode, carried out at The Ohio State University Campus Chemical Instrument Center (CCIC).

Flow Cytometry: Flow cytometric experiments were performed using an LSRII Fortessa (BD Biosciences) flow cytometer. Flow cytometric data was analyzed using Kaluza Analysis 2.1 (Beckman Coulter, Indianapolis, IN) software. SYTOX blue dead cell stain or near IR (780) fluorescent reactive dye (Invitrogen) were used to exclude dead cells. Fluorochrome-labeled human (hu) or murine (m) mAbs were used to stain cells for analysis by flow cytometry. Staining was done in FACS buffer (PBS + 2% FBS). A full list of antibodies can be found in **Supplementary Table 2**. Human Siglec-6/CD327 PE-conjugated antibody (Clone #767329, R&D Systems) was used to stain Siglec-6 on patient samples, and IgG_{2A} PE-conjugated Antibody (Clone #20102, R&D Systems) was used as control. Chemically biotinylated (BiotinTag Micro Biotinylation Kit; Sigma-Aldrich) JML-1 human IgG1[4] followed by staining with streptavidin PE (Thermo Fisher) was used for all other Siglec-6 surface staining experiments, and biotinylated human IgG1 isotype mAb was used as control. Absolute cell concentrations were obtained by quantitative flow cytometry using CountBright absolute counting beads (Invitrogen).

siRNA knockdown: Transient transfection of MEC1-002 cells was performed using the Lonza Cell Line Nucleofector™ Kit V (# VCA-1003) in the Nucleofector™ I/II/2b Device (Lonza) according to the manufacturer's specifications. Briefly, 1×10^6 cells were resuspended in 100 µl of nucleofector solution and mixed with the relevant siRNA. Nucleofection was performed using program M-13. Cells were rapidly transferred to preheated complete medium (RPMI 1640+10% FBS) and incubated for 48 hours at 37°C. For Siglec-6 knock-down, 500nM Siglec-6-specific SMARTpool siRNAs from Dharmacon (#E-004226-00-0010) was used. Target sequences are as follows: GGAUGAAAUACGGUUAUAC (#A-004226-4), CGUAUAGUUUCAGAUGUUA (#A-004226-15), CCCAUGACCUAAUUUAAAU (#A-004226-16), and GCCAUAACCUUGAUUGGAG (#A-004226-17). For non-targeting control, 500 nM of Accell

Green Non-Targeting siRNA (#D-001950-01-05) with sequence UGGUUUACAUGUCGACUAA was used. For SHP2 knock-down, 150 nM PTPN11 siRNA (AM51331) (sense strand sequence: CCAUGUUAUGAUUCCUGUtt, antisense strand sequence: ACAGCGAAUCAUAACAUGGgt) and related negative control (Silencer® Negative Control #1 siRNA) from ThermoFisher was used.

FACS sorting: Cell sorting was performed using a BD Bioscience BD FACSAria™ Fusion flow cytometer with a blue, red, yellow-green, violet, and UV laser (BD Biosciences). Splenocytes from Siglec-6 x TCL1 mice were selected and sorted for with the following antibody cocktail: mCD19-FITC (6D5) from Biolegend; mCD5-BUV737 (53-7.3) and mCD3-APC-Cy7 (145-2C11) from BD Biosciences; SYTOX blue dead cell stain (Invitrogen); and biotinylated JML-1 human IgG1 followed by staining with streptavidin PE (Thermo Fisher) for Siglec-6 staining. Siglec-6+ leukemic cells were identified as live, CD19+, CD5+, CD3-, and Siglec-6+. Briefly, cells were stained with antibody cocktail on ice for 1 hour before being washed once and resuspended in RPMI media. Cells were sorted through an 85 μm nozzle at a sheath pressure of 25 psi and a drop drive frequency of 60-70 kHz. 4-way purity sorting was used. Sorted purity achieved for all samples was > 98.0%.

***In vitro* migration and adhesion assay:** 12-hour migration assays were conducted using 6 μm pore size 24-well transwell plates (Corning). 0.2 x 10^6 BMSCs were plated in the bottom chambers and allowed to attach overnight. MEC1-002 or primary B cells in (1 × 10^6 in 100 μl serum free media) were added to the upper chamber and allowed to migrate towards BMSCs for 12 hours at 37°C. Transwell inserts were then removed, and migrated cells were counted using CountBright absolute counting beads (Invitrogen) using a LSRII Fortessa (BD Biosciences) flow cytometer. Fold migration was calculated vs baseline migration without stromal cells. In graphs where percent

migration is displayed, percent migration was calculated by normalizing to total number of cells seeded. For adhesion assay, 0.2 x 10^6 BMSCs were plated in 24 well plates and allowed to attach overnight. The following day, 1 × 10^6 MEC1-002 or primary B cells in RPMI media were seeded onto the BMSCs and allowed to adhere for 12 hrs at 37°C. Unattached cells were then washed twice and the cells left behind were trypsinized and counted using CountBright absolute counting beads (Invitrogen) using a LSRII Fortessa (BD Biosciences) flow cytometer. Fold attachment was calculated vs baseline attachment without stromal cells. In graphs where percent attached is displayed, percent attached was calculated by normalizing to total number of cells seeded. In both assays, cells were blocked with either JML-1 anti-Siglec-6 antibody or human IgG1 isotype control; or treated with ibrutinib (1 μM) or DMSO control for 1 hour before seeding into the transwells/onto BMSCs. Human CD19 PE (6D5) was used to identify and count only CD19+ B cells.

Confocal imaging: Cells were centrifugally concentrated on microscope slides using a Cytospin3 centrifuge (Thermo, Asheville, NC). Cells were then fixed in PBS/2% paraformaldehyde and permeabilized with 0.2% Tween 20. Slides were incubated in blocking solution (1% bovine serum albumin in PBS+ 0.1% Tween 20) and stained for Siglec-6 (Sigma Aldrich, # HPA009084) or DOCK8 (Origene, #TA506484) by incubating with the primary antibodies overnight at 4°C, followed by incubation with secondary antibodies, goat anti-rabbit IgG Alexa Fluor 488 (Invitrogen) or goat anti-mouse IgG1 Alexa Fluor 594 (Invitrogen), respectively. Texas Red-X phalloidin (Invitrogen) was used to stain F-actin during the secondary antibody incubation step. Nuclei were stained blue with DAPI (Vector Laboratories). Olympus Fluoview 1000 Laser Scanning Confocal microscope at the Ohio State University Campus Microscopy and Imaging Facility was used for capturing images. For **Fig. 6c** and **supplementary Fig. 7a**, images were

taken using a ×60 objective, and z-stacking was performed at pixel resolution 512 x 512. For **Fig. 1a,** and **supplementary Fig. 4b**, images were captured using a ×20 objective at pixel resolution 512 x512. Equipment specifications are as follows: a) Upright Microscope: Olympus BX61F/BX62F; b) Detectors: 4 filter-based fluorescence PMT detectors and one transmitted, DIC detector; c) Available Objectives: UPLSAPO 10x, N.A. 0.4 LUCPLFLN 20x, N.A. 0.45 UPLSAPO 20x Oil, N.A. 0.85 UPLFLN 40x Oil, N.A. 1.3 PLAPONSC 60x Oil, N.A. 1.4; d) Image info: OIB or OIF Olympus format, 12 bit gray scale 24 bit color, JPEG/BMP/TIFF/AVI image formats also available, and pixel resolution formats range from 64 x 64 up to 4096 x 4096.

Co-culture immunofluorescent microscopy: For live co-culture adhesion assays, BMSCs and CLL/healthy donor B cells were labelled with BioTracker 490 Green or BioTracker 655 Red cytoplasmic membrane dyes respectively, according to manufacturer's instructions (MilliporeSigma). Co-culture immunofluorescence images were captured using ECHO Revolve R4 microscope, at 10x magnification.

Cell Fractionation: 10 X 10^6 MEC1-002 (WT, Siglec-6 KO or DOCK8 KO), B-CLL or normal B cells were plated in 12 well plates, treated with 6 μg sTn for1 hour. Membrane, nuclear and cytoplasmic fractions were then prepared according to manufacturer's protocol (Thermo Fisher #78840).

Cdc42 activation assay: 10 X 10^6 MEC1-002 (WT, Siglec-6 KO or DOCK8 KO) cells were plated in 12 well plates, treated with 6μg sTn for 1 min or blocked with 10 μg anti-Siglec-6 mAb for 30 min followed by sTn treatment for 1 min. Cdc42 activation assay was carried out according to manufacturer's protocol (Abcam, ab211163)

Siglec-6-sTn interaction studies: For ELISA based assay, Pierce™ Protein A Coated Plates (Thermo Scientific) were coated with 2 µg/ml Siglec-6 Fc fusion protein or IgG Fc (Acro Biosystems) in 50 mM carbonate bicarbonate buffer (pH 9.5), overnight at 4°C. After blocking for 1 hour at room temperature with PBS/2%BSA, biotinylated sTn (sTn-B) (GlycoNZ, #0058BP) was added at different concentrations. Binding was measured using tetramethyl benzidine substrate (R&D Systems) according to manufacturer's instructions. For cell line-based assay, the siglec negative DT-40 cell line was transfected either with 1 µg CMV-Siglec-6 construct or CMV empty vector[5] using the Amaxa Nucleofector system. 24 hrs post transfection, cells were either blocked with isotype mAb or Siglec-6 blocking mAb, followed by sequential probing with sTn-B and streptavidin PE. Median fluorescence intensity as a readout of binding was measured via flow cytometry analysis. To test binding of Siglec-6 to sTn on CLL-BMSCs, CLL-BMSCs were blocked with anti-Sialyl Tn antibody [STn 219] (Abcam) or IgG1 isotype control for 30 min at 4°C. Cells were also treated with 0.6 U sialidase (MilliporeSigma) for 30 min at 37°C[6]. Following this, cells were washed, and Siglec-6 Fc fusion protein was added for 30 min at 4°C. After this, cells were washed and stained with biotinylated JML1 for 1 hr, after which cells were washed again and stained with PE-Cy7 Streptavidin (BD Biosciences) for 30 min. Flow cytometry was used to quantify Siglec-6 binding to CLL-BMSC based on fluorescence in the PE-Cy7 channel.

Western blotting: For all western blotting experiments, cells were lysed with RIPA buffer (CellSignaling Technology, #9806) supplemented with protease and phosphatase inhibitors. Lysate concentration was determined using Pierce™ BCA Protein Assay Kit (ThermoFiser Scientific, #23225) and proteins were analyzed via western blotting. A full list of antibodies can be found in **Supplementary Table 3**. Images were captured using SuperSignal™ West Pico PLUS Chemiluminescent Substrate (#34577).

Co-immunoprecipitation (Co-IP): Co-IP experiments were performed according to manufacturer's instructions (Dynabeads™ Protein G Immunoprecipitation Kit, Thermo Fisher). Anti-human Siglec-6 antibody (clone 2G6, LifeSpan Biosciences) or mouse IgG2b isotype control antibody was used to pull down Siglec-6 protein, and followed by immunoblotting with DOCK8 antibody (EPR1251, Abcam). Rabbit Siglec-6 (polyclonal, Abcam) was used to confirm Siglec-6 pull down via immunoblotting. For co-IP experiments with BMSCs, Siglec-6 Fc fusion protein or IgG Fc (Acro Biosystems) was conjugated to Protein A agarose beads (MilliporeSigma) to test interaction of Siglec-6 with sTn+ proteins. Pull down of sTn+ proteins were confirmed using anti-human sialyl Tn antibody (B35.1, LifeSpan Biosciences). Multiple bands were observed in both western blotting and IP experiments with sTn corresponding to a band detected at ~ 150 kDa according to company product specifications. Anti- Siglec-6 antibody (polyclonal, Abcam) was used to confirm conjugation of Siglec-6 to Protein A agarose beads.

B-cell homing assay: NOD.Cg-Prkdc^{scid} Il2rg^{tm1Wjl}/SzJ (NSG) mice were obtained from Jackson Laboratory (005557). B-CLL or MEC1-002 cells were injected into the tail vein of 4- to 8-week-old NSG mice (10 X 10^6 cells per mouse). For blocking experiments, B-CLL or MEC1-002 cells were incubated 1 hour before injection with JML-1 mAb or human IgG1 isotype control antibody. 24 hours after injection, mice were sacrificed, and bone marrow and spleen were collected. Human B cells from the different organs were detected by flow cytometry using human specific anti-CD45 antibody and absolute number of migrated cells were counted using CountBright absolute counting beads (Invitrogen).

T cell expansion: Humanized mouse T cells (huCD3 T cells) were expanded from humanized CD3ε mouse which express the human epitope of the CD3ε chain[7,8] by seeding at 1 million cells

in 200µL IMDM media with concanavalin A (Invitrogen) in the presence of 100 U/mL IL-2 (R & D systems) and 2-mercaptoethanol over a 7 day culture.

***Ex vivo* cytotoxicity assay:** B cells were isolated from the spleens of TCL1 or hSiglec-6 x TCL1 mice using the EasySep™ Mouse B Cell Isolation Kit (StemCell Technologies). RosetteSep enrichment kit (#15064, StemCell Technologies) was used to isolate B cells from CLL and healthy donor whole blood samples. Target B cells were labelled with CellTrace violet (Invitrogen) and cultured with concanavalin A activated effector huCD3 T cells, along with the CD123 control T-biAb or Siglec-6 T-biAb (6 nM) for 5 hrs. Effector: target ratio used was 5:1. CD123 T-biAb was obtained from Xencor (Xmab14045), and Siglec-6 T-biAb in scFv-Fc format (silenced Fc) based on mAb JML-1 was recently described[5]. Cells were then stained with Annexin V and propidium iodide (PI) (Leinco Technologies) to calculate % target dead cells by flow cytometry. To test the Siglec-6 T-biAb against treatment-naïve CLL patients using autologous T cells, PBMCs from 5 CLL patients were isolated using Ficoll density-gradient centrifugation and were cultured with either Siglec-6 targeting T-biAb (6 nM) or CD123 T-biAb (6 nM) for 7 days. Percentage of CLL cells (CD5+CD19+CD3-) that were killed was measured using annexin V/PI staining and flow cytometry analysis. % Killing = (100 - % Annexin V and PI negative cells)

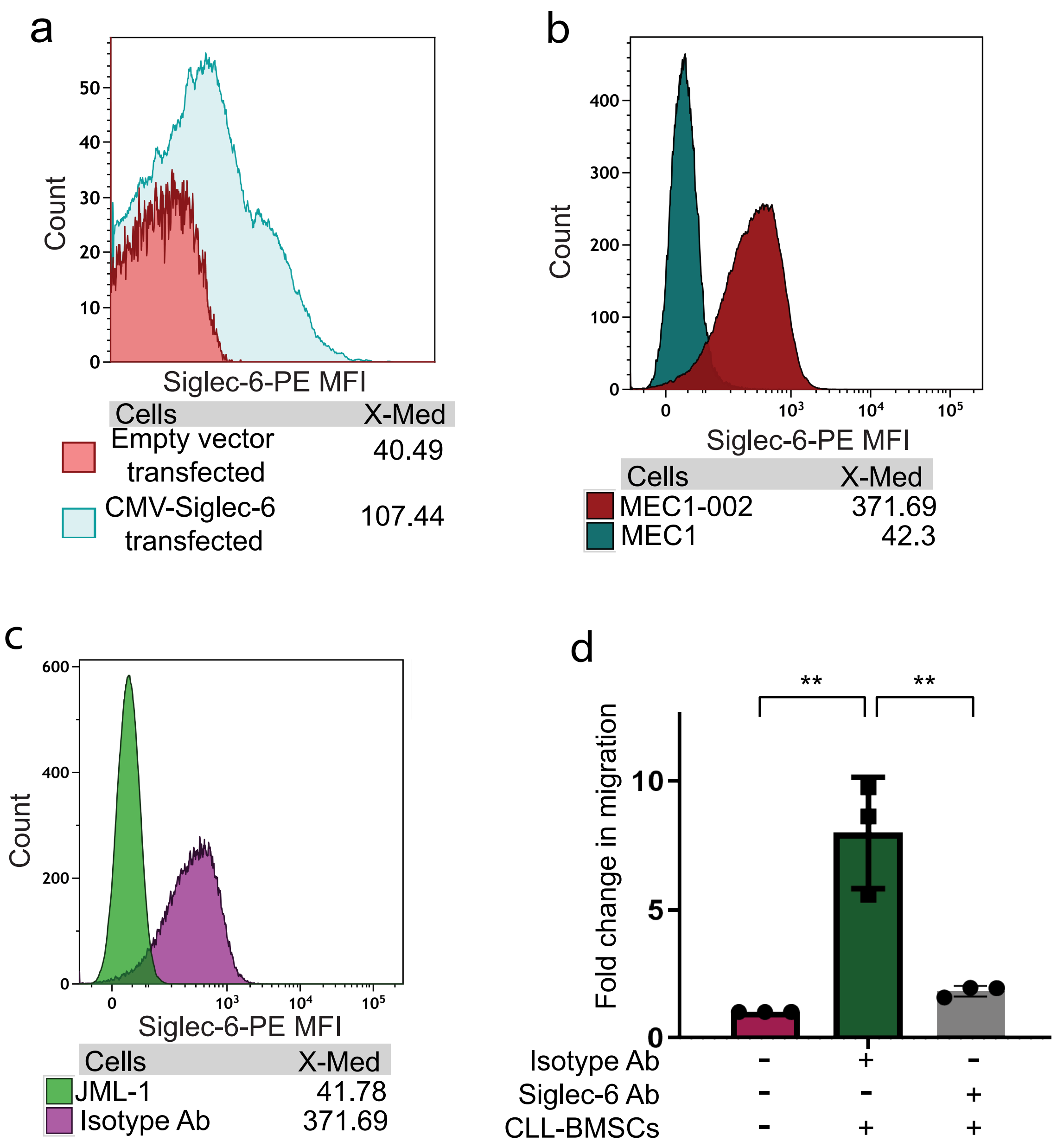

Supplementary Fig. 1: JML-1 is a blocking antibody specific to Siglec-6. a) Surface Siglec-6 expression on DT-40 cells transfected with CMV-Siglec-6 construct was higher when compared to empty vector transfected cells, detected by flow cytometry. **b)** Overlay histogram shows median fluorescence intensity for Siglec-6 staining in MEC1 cells and Siglec-6+ enriched MEC1-002 cells. **c)** Flow cytometry analysis showing blocking of Siglec-6 using the JML-1 anti-Siglec-6 mAb antibody. **d)** Transwell migration analysis of MEC1-002 cells towards CLL-BMSCs. MEC1-002 cells were blocked with JML- 1 Siglec-6 mAb or isotype mAb for 1 hr prior to the start of the assay. Siglec-6 mAb decreases migration of MEC1-002 cells by 4.4-fold (P = 0.0002), n = 3 biological replicates**. (a-c):** Representative data shown from experiments conducted with n = 3 biological replicates. Graphs show mean ± standard error of the mean. *P<0.05; **P<0.005 by mixed effect modeling. ns: not significant. Source data provided in source data file.

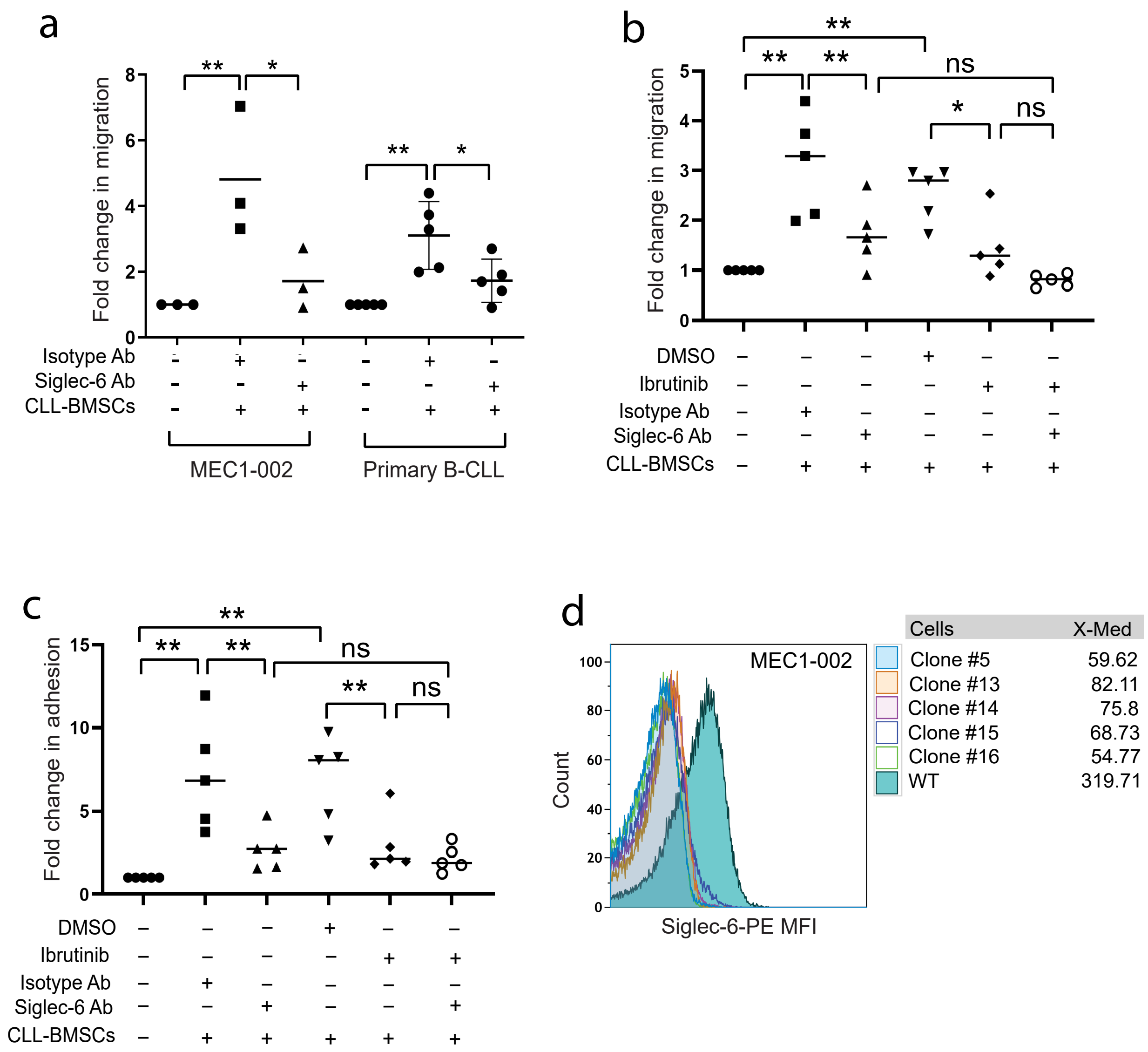

Supplementary Fig. 2. Siglec-6 promotes migration of B-CLL cells. a) Comparison of *in vitro* migration between MEC1-002 and primary B-CLL cells (n = 3 biological replicates). Transwell migration assay **(b)** and adhesion assay **(c)** looking at the effect of ibrutinib on migration and adhesion of B-CLL cells. Ibrutinib significantly reduced migration [t(20) = 2.87, P = 0.009] and adhesion [t(20) = 3.43, P = 0.003] of B-CLL cells when compared to DMSO control. n = 5 patients. **d)** Overlay histogram showing loss of Siglec-6 expression on expanded single cell knock-out clones after CRISPR mediated knock-out of Siglec-6 in MEC1-002 cells, compared to MEC1-002 WT cells. Graphs show mean ± standard error of the mean. **P*<0.05; ***P*<0.005 by mixed effect modeling. ns: not significant. Source data provided in source data file.

a

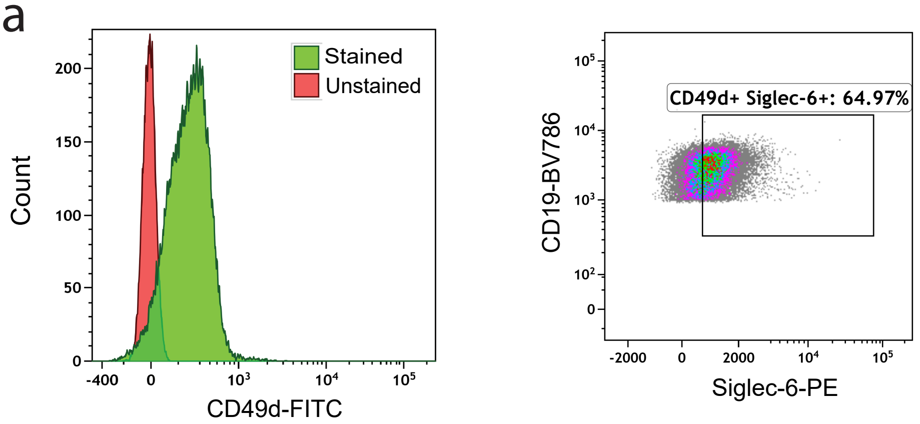

	CD49d MFI	Siglec-6 MFI
**Patient 1**	52.97	2268
**Patient 2**	260.13	854.56
**Patient 3**	138.68	1101.29
**Patient 4**	114.74	1075.04
**Patient 5**	144.74	1027.02
**Patient 6**	174.42	642.35

b

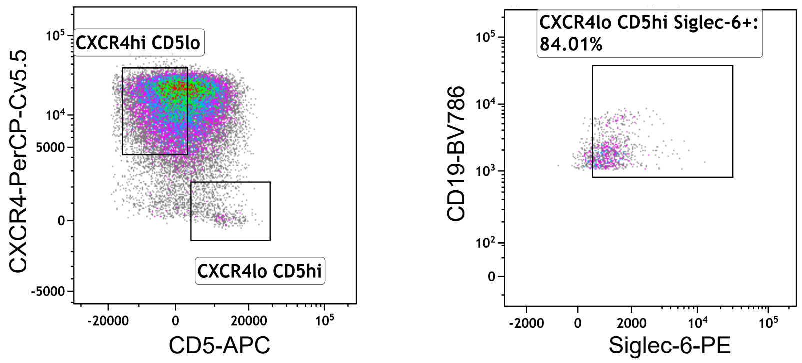

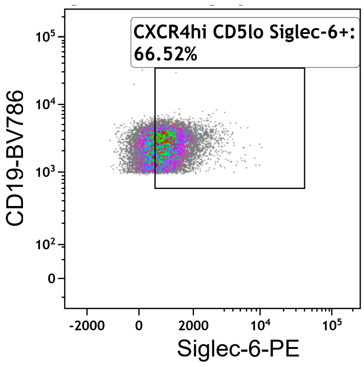

	Siglec-6 MFI	
	CXCR4lo/CD5hi	CXCR4hi/CD5lo
Patient 1	1954.85	2332.71
Patient 2	992.8	749.87
Patient 3	868.46	863.07
Patient 4	941.48	1030.62
Patient 5	1174.12	907.52
Patient 6	871.39	595.17

Supplementary Fig. 3. Siglec-6 expression on different subsets of CLL cells. a) Flow cytometry analysis showing CD49d expression on a representative CLL patient sample, and Siglec-6 expression on CD49d+ B-CLL cells. No correlation was found between CD49d and Siglec-6 expression. **b)** Flow cytometry analysis showing Siglec-6 expression on CXCR4lo/CD5hi and CXCR4hi/CD5lo CLL cells. No differences in Siglec-6 expression were observed. n = 6 patient samples were used. Source data provided in source data file.

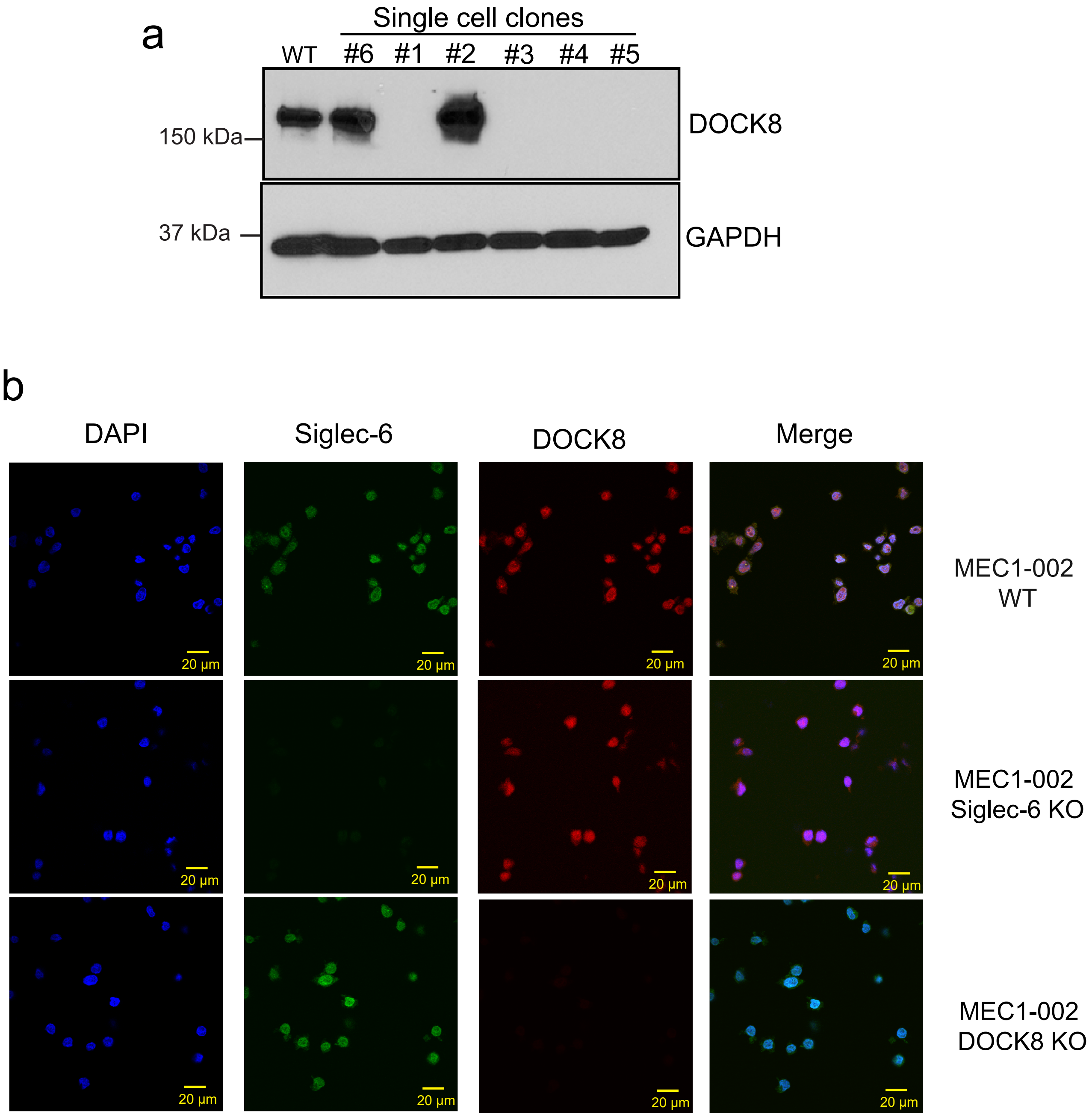

Supplementary Fig. 4. Siglec-6 and DOCK8 are both essential for *in vitro* migration of MEC1-002 cells. a) Immunoblot showing loss of DOCK8 expression in expanded single cell knock-out clones after CRISPR mediated knock-out of DOCK8 in MEC1-002 cells, compared to MEC1-002 WT cells. **b)** Confocal immunofluorescent analysis of Siglec-6 and DOCK8 expression in MEC1-002 WT, Siglec-6-KO or DOCK8 KO cells to confirm loss of Siglec-6 or DOCK8 expression, data from one replicate. Source data provided in source data file.

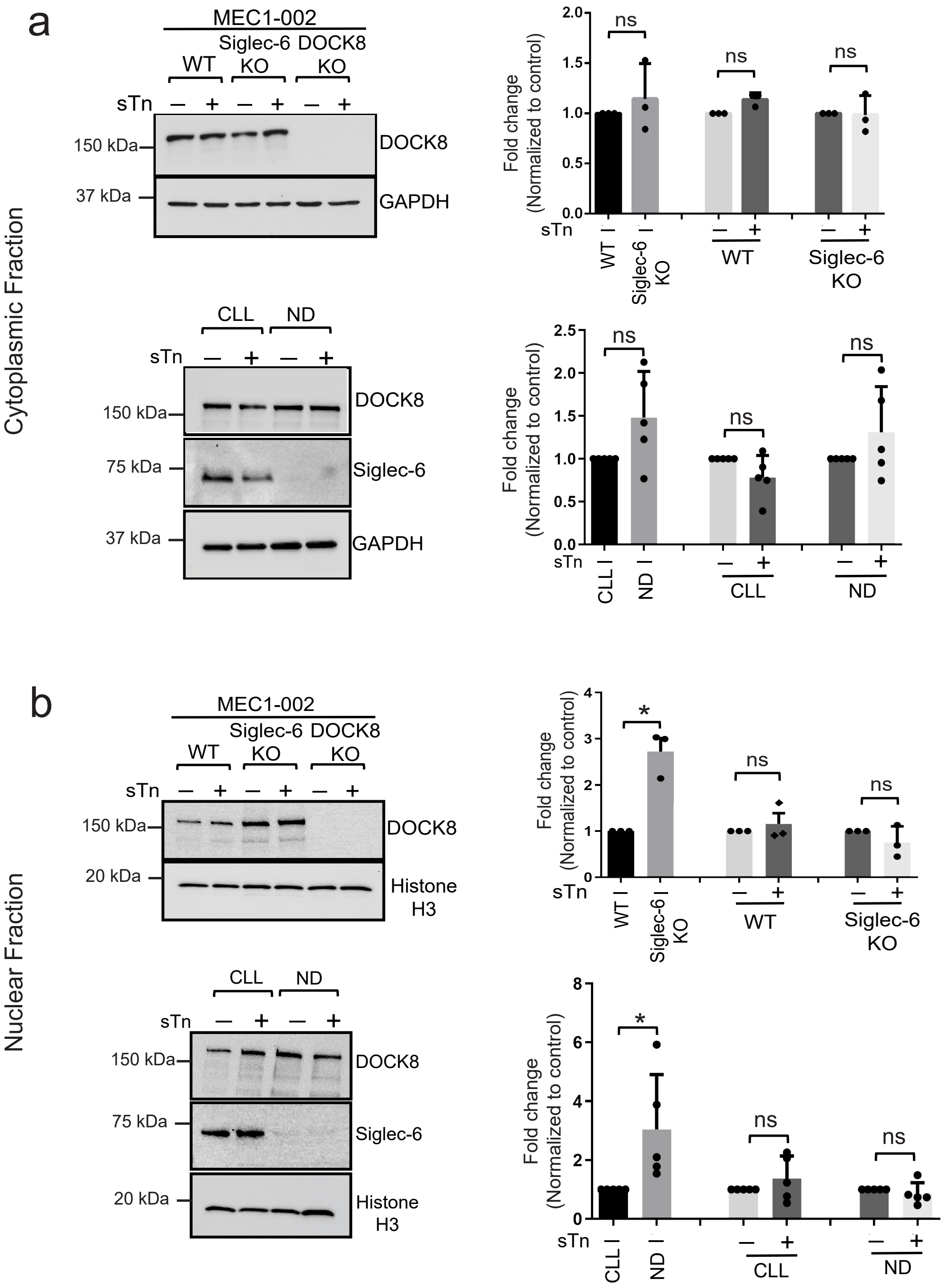

Supplementary Fig. 5. Siglec-6 is essential for membrane localization of DOCK8. MEC1-002 (WT and Siglec-6 KO), B-CLL cells and normal healthy donor (ND) B cells were unstimulated or treated with sTn for 1 hour. Protein lysates were separated into **(a)** cytoplasmic and **(b)** nuclear fractions. Histone H3 and GAPDH were used as a loading control for the nuclear and cytoplasmic fraction, respectively. All experiments performed with n = 3 biological replicates for cell lines, and n = 5 independent donors for primary cells. Graphs show mean ± standard error of the mean. **P*<0.05; ***P*<0.005 by mixed effect modeling. ns: not significant. Source data provided in source data file.

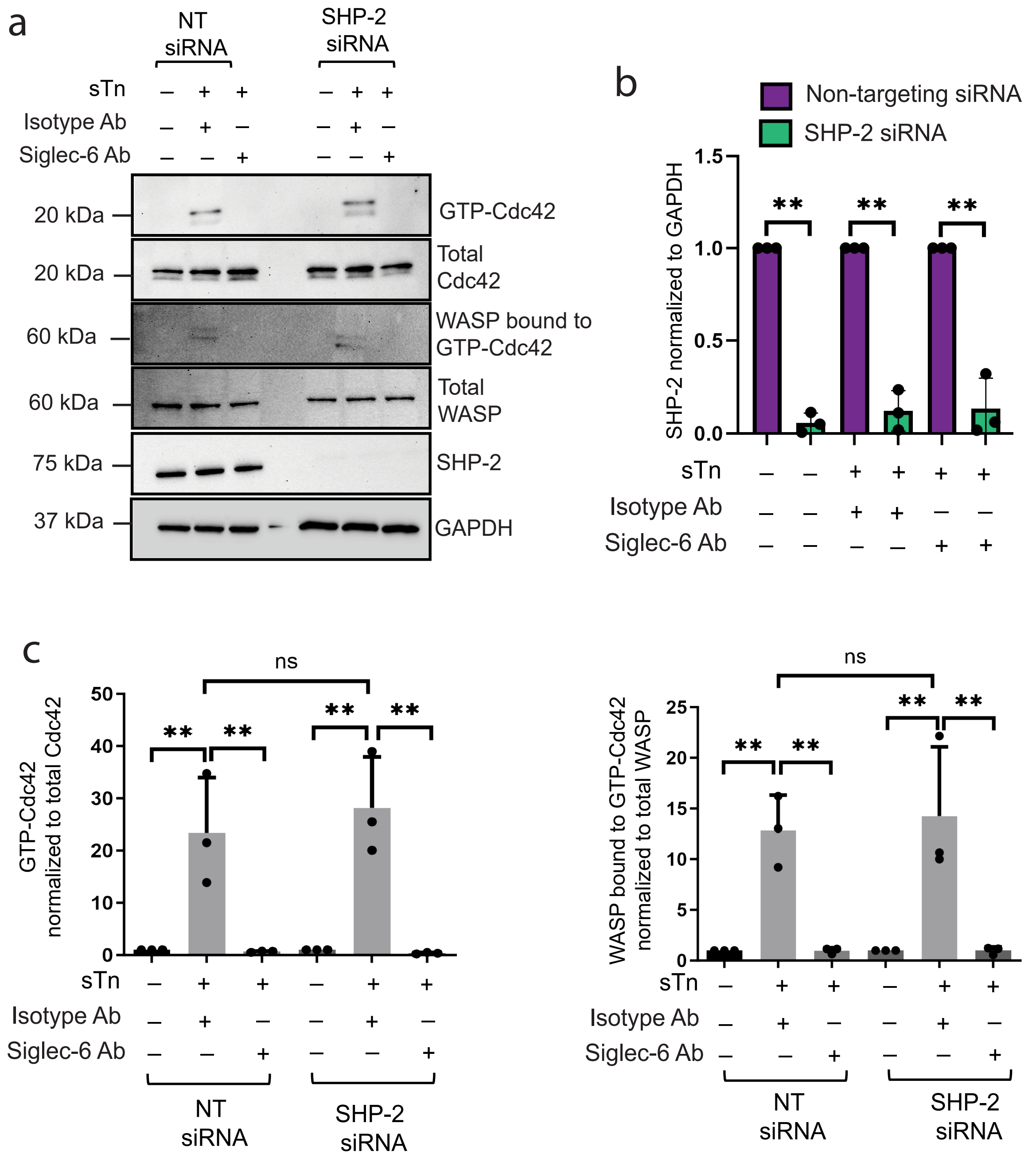

Supplementary Fig. 6. sTn mediated Siglec-6 and DOCK8 dependent Cdc42 activation is independent of SHP-2. a) *SHP-2* was knocked down in MEC1-002 using SHP-2 siRNA. Immunoblotting analysis shows no differences in GTP-Cdc42 [t(10) = 1.51, *P* = 0.2] and WASP [t(10) = 1.14, *P* = 0.3] protein levels between non-targeting (NT) siRNA and SHP-2 siRNA transfected MEC1-002 cells**. b and c)** GTP-Cdc42 levels, active WASP and SHP-2 protein levels were measured by densitometric quantification of immunoblots and normalized to total Cdc42, total WASP and GAPDH protein, respectively**.** n = 3 biological replicates. Graphs show mean ± standard error of the mean. **P*<0.05; ***P*<0.005 by mixed effect modeling. ns: not significant. Source data provided in source data file.

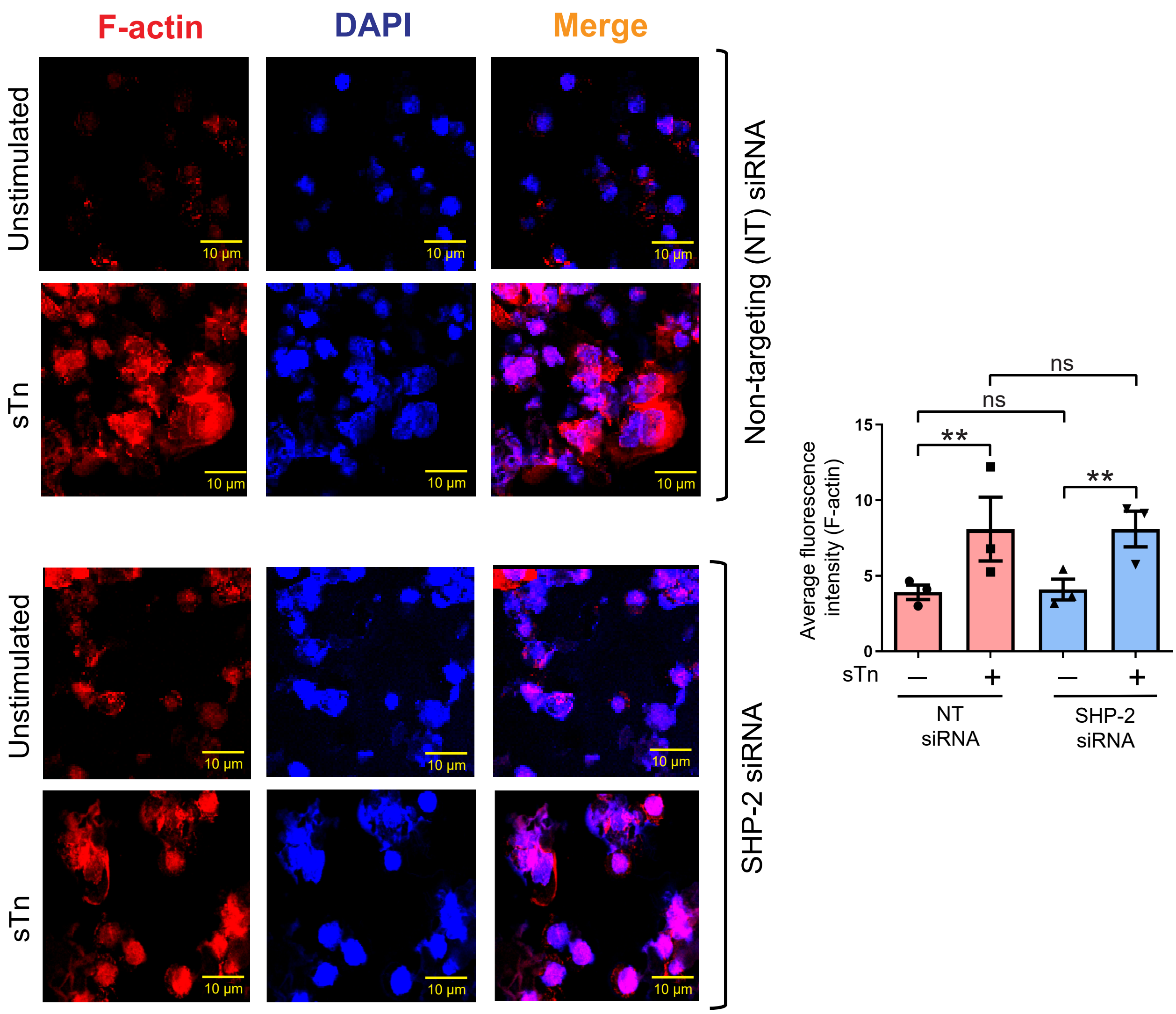

Supplementary Fig. 7. sTn mediated Siglec-6 and DOCK8 dependent actin polymerization is independent of SHP-2. Confocal immunofluorescent analysis of F-actin filaments showed no differences in F-actin polymerization between non-targeting (NT) siRNA and SHP-2 siRNA transfected MEC1-002 cells [$t(119) = -0.04$, $P = 0.97$]. ImageJ was used to quantify fluorescence intensity from F-actin staining from 10 random fields each from n = 3 biological replicates. Graphs show mean ± standard error of the mean. *$P<0.05$; **$P<0.005$ by mixed effect modeling. ns: not significant. Source data provided in source data file.

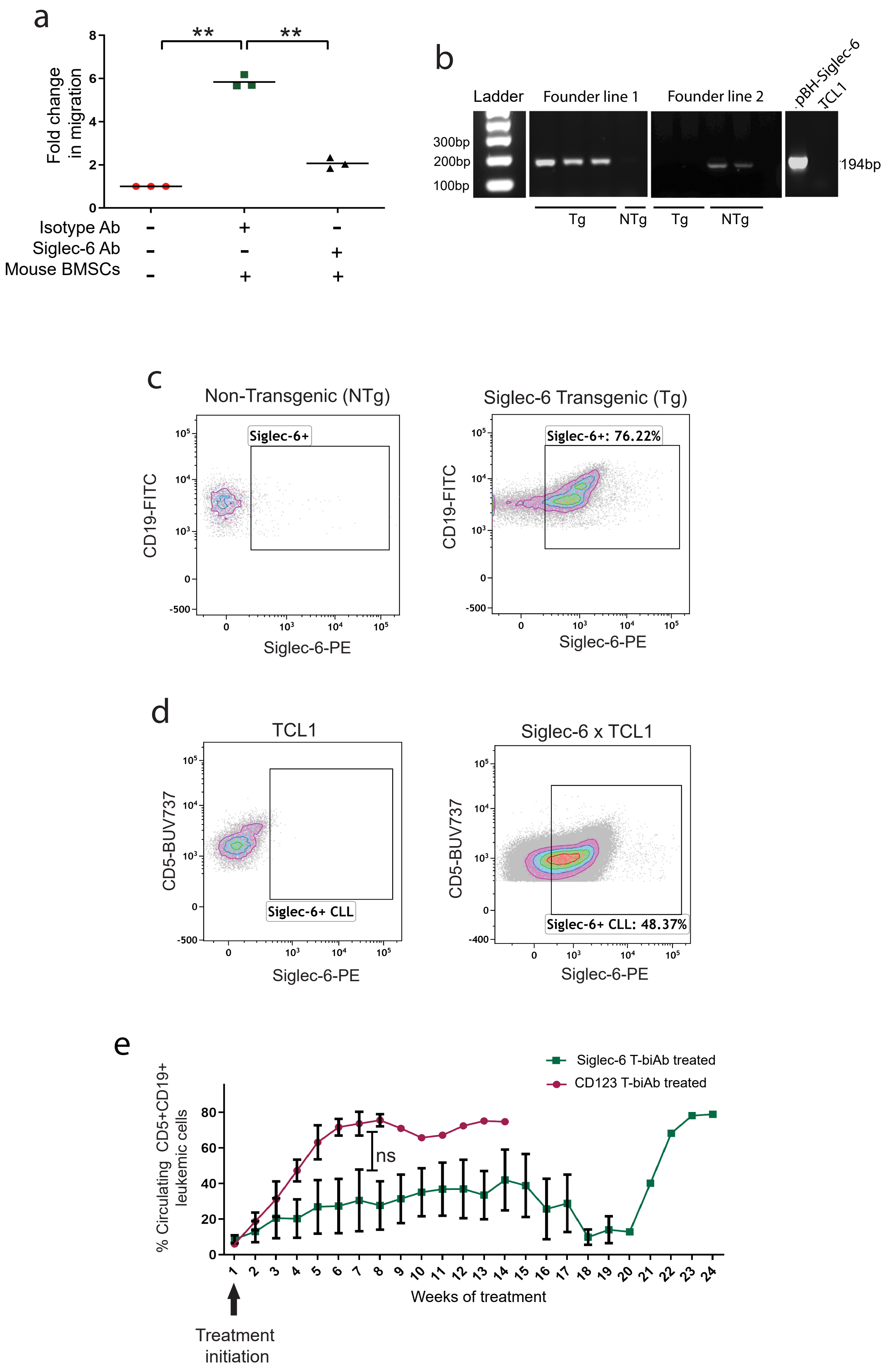

a
Fold change in migration
**
**
Isotype Ab
Siglec-6 Ab
Mouse BMSCs
b
Ladder
Founder line 1
Founder line 2
pBH-Siglec-6
TCL1
300bp
200bp
100bp
194bp
Tg
NTg
Tg
NTg
c
Non-Transgenic (NTg)
Siglec-6 Transgenic (Tg)
Siglec-6+
Siglec-6+: 76.22%
CD19-FITC
Siglec-6-PE
d
TCL1
Siglec-6 x TCL1
Siglec-6+ CLL
Siglec-6+ CLL: 48.37%
CD5-BUV737
Siglec-6-PE
e
Siglec-6 T-biAb treated
CD123 T-biAb treated
% Circulating CD5+CD19+ leukemic cells
ns
Weeks of treatment
Treatment initiation

f

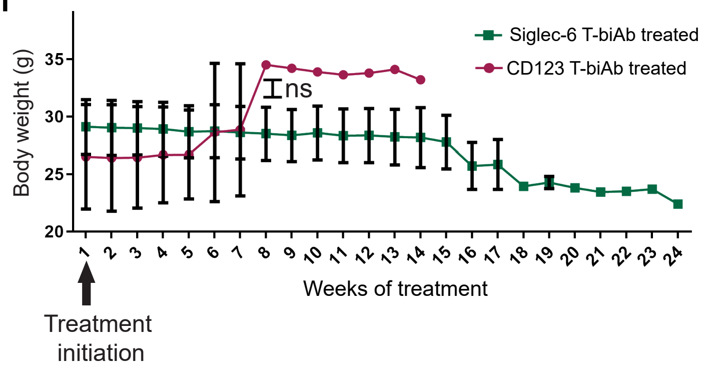

Supplementary Fig. 8. Siglec-6 mAb inhibits *in vitro* migration of Siglec-6 positive MEC1-002 cells to mouse BMSCs and Siglec-6 targeted T-biAb improves overall survival of mice with hSiglec-6×TCL1 leukemia. a) Transwell migration analysis of B-CLL cells towards primary mouse BMSCs cultured from bone marrow flushes of the C57Bl/6 mouse femur (n = 3 mice). The Siglec-6 mAb significantly inhibited migration [t(4) = 14.86, P = 0.0001] when compared to isotype mAb, n=3 mice. **b)** Genotyping and PCR analysis showing Siglec-6 expression in two founder lines of the hSiglec-6 Tg mice. pBH-Siglec-6 construct was used as positive control and TCL1 mouse was used as negative control**. c)** Flow cytometry analysis shows expression of Siglec-6 on a subset of CD19+ B cells in peripheral blood of a Siglec-6 transgenic mouse, but not non-transgenic mouse (16-week male). **d)** Flow cytometry analysis shows expression of Siglec-6 on a subset of CD5+CD19+CD3- leukemic B cells in peripheral blood of Siglec- 6 x TCL1 transgenic mice, but not TCL1 mice (11-month male). **e)** Siglec-6 T-biAb treated mice on an average display a trend towards lower circulating leukemic burden (data not significant). **f)** Body weights of the control and treated mice were compared to confirm absence of treatment-related toxicity and decrease in bodyweight. No significant difference in body weights was observed. CountBright absolute counting beads were used to count the number of cells that have migrated. Graphs show mean ± standard error of the mean. *P<0.05; ** P<0.005. ns: not significant. Source data provided in source data file.

Supplementary Table 1. Siglec-6 associates with DOCK8, a guanine nucleotide exchange factor. The table depicts list of proteins that significantly associate with Siglec-6 (log2 fold change > 1.5 and $P < 0.05$) based on mass spectrometry analysis using JML-1 Siglec-6 mAb to pull down proteins in MEC1-002 protein lysate.

Protein	log2 (Fold Change)	p-value
DOCK8	2.906890596	0.04
LRCH3	2.655351829	0.042
GTF2B	2.40599236	0.015
GMPPA	2.169925001	0.046
DDX24	1.925999419	0.008
CORO1B	1.584962501	0.044
POLDIP3	1.5360529	0.0004
CAPZA2	1.5360529	0.021

Supplementary Table 2. Antibodies for Flow Cytometry

Name	Fluorochrome	Catalog number	Company/Source	Clone	Dilution	Lot No.
CD5	BV510	563380	BD Biosciences	UCHT2	1:30	
CD3	BV421	562426	BD Biosciences	UCHT1	1:30	8316512
CD19	BV786	563325	BD Biosciences	SJ25C1	1:30	9248984
CD73	PECF594	562817	BD Biosciences	AD2	1:30	195686
CD90	PerCP-Cy5.5	561557	BD Biosciences	5E10	1:30	121727
CD34	BV421	562577	BD Biosciences	581	1:30	7718
CD45	APC	555485	BD Biosciences	D058-1283	1:10	9291059
CD49d	FITC	304316	Biolegend	9F10	1:30	B352326
CXCR4	PerCP-Cy5.5	306508	Biolegend	12G5	1:30	B342627
Sialyl Tn	Unconjugated	ab115957	Abcam	M1-14D12	1:25	GR3356902-1
anti-mouse IgG1	PE-Cy7	25-4015-82	ThermoFisher	M1-14D12	1:25	2034749
CD45	PECF594	562420	BD Biosciences	30-F11	1:30	1342647
CD45	APC-Cy7	103116	Biolegend	30-F11	1:30	B370047
CD19	FITC	115506	Biolegend	6D5	1:30	B361055
CD3	BUV395	564000	BD Biosciences	SK7	1:30	2215094
CD5	BUV737	612809	BD Biosciences	53-7.3	1:30	2111304
CD3	APC-Cy7	557596	BD Biosciences	145-2C11	1:30	1336946
Siglec-6	PE	FAB2859	R&D Systems	767329	1:10	ADZQ0221091
Siglec-6	Biotin		Collaborator	JML-1	1:100	Not applicable
Streptavidin-PE	PE	554061	BD Biosciences		1:30	1228892

Supplementary Table 3. Antibodies for Western Blotting, immunoprecipitation and immunofluorescence

Name	Catalog number	Company/Source	Clone	Dilution	Notes	Lot No.
GAPDH	G5262	Millipore Sigma	6C5	1:600000	For western blotting	3587127
WASP	4860	CST	Polyclonal	1:1000	For western blotting	1
Cdc42	2462	CST	Polyclonal	1:1000	For western blotting	6
AIF	5318	CST	D39D2	1:1000	For western blotting	3
Histone H3	4499	CST	D1H2	1:1000	For western blotting	9
Siglec-6	ab262851	Abcam	Polyclonal	1:1000	For western blotting	GR3283489-1
Siglec-6	LS-C196855	LifeSpan Biosciences	2G6	1:50	For IP	126400
DOCK8	ab175208	Abcam	EPR1251	1:3000	For western blotting	GR3287831-1
Sialyl Tn	LS-C170901-0.5	LifeSpan Biosciences	B35.1	1:100	For western blotting	226386
Anti-mouse IgG-HRP	1706516	Biorad	Polyclonal	1:3000	Secondary antibody	64512899
Anti-Rabbit IgG-HRP	7074	CST	Polyclonal	1:1000	Secondary antibody	30
Siglec-6	HPA009084	Sigma Aldrich	Polyclonal	1:500	For immunofluorescence	1536
DOCK8	TA506484	Origene	OTI2C11	1:500	For immunofluorescence	A001
Anti-Rabbit IgG-AlexaFluor 594	A-11005	ThermoFisher	Polyclonal	1:2000	Secondary antibody	UD28413
Anti-mouse IgG-AlexaFluor 488	A-11008	ThermoFisher	Polyclonal	1:2000	Secondary antibody	UD30542

Supplementary Table 4. List of proteins identified from mass spectrometry analysis of Siglec-6 interacting partners

Protein	Log2 Fold Change	-Log10 (p-value)
DOCK7	3.906890596	0.886056648
RRBP1	3.217230716	0.443697499
FGR	3.03562391	0.537602002
SEMA7A	2.981852653	0.585026652
DOCK8	2.906890596	1.397940009
LRCH3	2.655351829	1.37675071
FLNA	2.560714954	1.15490196
PPP1R9B	2.560714954	0.602059991
USP7	2.5360529	0.958607315
MPRIP	2.510961919	0.886056648
GINS3	2.510961919	0.585026652
FRG1	2.432959407	1
GTF2B	2.40599236	1.823908741
VPS4A	2.263034406	0.638272164
CAD	2.232660757	0.468521083
SUN2	2.201633861	1.013228266
GMPPA	2.169925001	1.337242168
NEK7	2.10433666	1.283996656
DCAF7	2.10433666	1.229147988
glyA	2.10433666	0.443697499
POP1	2	0.638272164
DDX24	1.925999419	2.124938737
PAF1	1.887525271	0.568636236
AP3D1	1.887525271	0.283996656
SMC4	1.887525271	0.283996656
MRPS34	1.847996907	1
TXNDC12	1.847996907	0.552841969
NUP88	1.765534746	1.013228266
ACTN4	1.722466024	1.180456064
Serpina1a	1.678071905	0.443697499
DDX18	1.632268215	0.677780705
IPO7	1.632268215	0.552841969
CORO1B	1.584962501	1.356547324
GSN	1.584962501	0.886056648
MYO18A	1.584962501	0.795880017
RBM27	1.584962501	0.744727495
NDRG3	1.584962501	0.408935393
POLDIP3	1.5360529	3.431798276
CAPZA2	1.5360529	1.677780705
LYN	1.5360529	0.920818754
LIMA1	1.5360529	0.677780705
COPZ1	1.5360529	0.537602002
GABPA	1.5360529	0.420216403
CTSS	1.5360529	0.420216403
GMDS	1.485426827	0.397940009
MAGT1	1.485426827	0.397940009

PPP1R18	1.432959407	0.744727495
ADAR	1.432959407	0.744727495
SLC25A1	1.432959407	0.744727495
CTNNA1	1.432959407	0.744727495
MVP	1.432959407	0.721246399
C7orf50	1.432959407	0.619788758
SPN	1.432959407	0.619788758
LRCH4	1.432959407	0.585026652
LMO7	1.432959407	0.468521083
TIMMDC1	1.432959407	0.408935393
SCARB1	1.432959407	0.408935393
RABGGTB	1.432959407	0.408935393
DHCR7	1.432959407	0.408935393
IRF8	1.432959407	0.408935393
HAX1	1.432959407	0.37675071
DIAPH1	1.432959407	0.236572006
TAP2	1.432959407	0.236572006
SYMPK	1.432959407	0.236572006
KTN1	1.432959407	0.236572006
MAP4	1.432959407	0.236572006
RIOX2	1.378511623	1.420216403
TWF2	1.378511623	1.119186408
CSNK1A1	1.378511623	0.853871964
atpD	1.378511623	0.552841969
LUZP1	1.378511623	0.455931956
SACM1L	1.378511623	0.387216143
PUM1	1.378511623	0.387216143
RFX5	1.378511623	0.387216143
VIM	1.321928095	1.244125144
DHX30	1.321928095	0.552841969
NUP85	1.321928095	0.552841969
SPATS2L	1.263034406	1.958607315
EXOSC2	1.263034406	0.920818754
ECHDC1	1.263034406	0.886056648
RPL15	1.201633861	2.619788758
DECR1	1.201633861	2.065501549
ARPC5L	1.201633861	1
MYO1D	1.201633861	0.657577319
WDR1	1.201633861	0.657577319
MYO5A	1.201633861	0.568636236
MEMO1	1.201633861	0.522878745
RACGAP1	1.201633861	0.37675071
SLC29A1	1.201633861	0.346787486
DDX41	1.201633861	0.200659451
RPL36AL	1.137503524	2.180456064
CORO1C	1.137503524	1.187086643
CIRBP	1.137503524	0.823908741

MX2	1.137503524	0.795880017
FLII	1.137503524	0.721246399
MYO1G	1.137503524	0.638272164
ANKRD44	1.137503524	0.619788758
RRAS2	1.137503524	0.585026652
HNRNPUL1	1.137503524	0.508638306
Alb	1.137503524	0.494850022
LGB	1.137503524	0.455931956
ELAVL1	1.070389328	2.30980392
NUP93	1.070389328	2.113509275
PPP1R12C	1.070389328	1.167491087
TNIP1	1.070389328	1.086186148
ACTR2	1.070389328	0.958607315
ATL3	1.070389328	0.853871964
TWF1	1.070389328	0.823908741
WDR77	1.070389328	0.795880017
TDRD3	1.070389328	0.508638306
FAM98B	1.070389328	0.443697499
EPRS	1.070389328	0.397940009
GNB2	1.070389328	0.27572413
RANGAP1	1	2.113509275
RPL31	1	1.420216403
REPIN1	1	1.148741651
ADA	1	0.920818754
ABHD14B	1	0.853871964
ZCCHC3	1	0.769551079
AKAP2	1	0.619788758
TRIM38	1	0.508638306
PFL2215w	1	0.431798276
CLTC	1	0.27572413
TRIM56	1	0.207608311
RPL13A	0.925999419	1.823908741
ARPC5	0.925999419	1
MYO1C	0.925999419	0.638272164
TLN1	0.925999419	0.585026652
LIG3	0.925999419	0.568636236
MTCH2	0.925999419	0.552841969
DBN1	0.925999419	0.494850022
KEAP1	0.925999419	0.443697499
RABEP2	0.925999419	0.431798276
COX7A2	0.925999419	0.346787486
GNPNAT1	0.925999419	0.346787486
NDUFAF4	0.925999419	0.337242168
RHOG	0.925999419	0.292429824
HBA	0.925999419	0.292429824
GNAI2	0.847996907	1.657577319
ATAD3B	0.847996907	1.638272164

ATAD3A	0.847996907	1.214670165
HNRNPA3	0.847996907	1.173925197
TFAM	0.847996907	1.004364805
FLOT1	0.847996907	0.886056648
DTD1	0.847996907	0.638272164
PPP1R12A	0.847996907	0.585026652
ACTR3	0.847996907	0.585026652
RRS1	0.847996907	0.552841969
HIST1H1E	0.847996907	0.537602002
GTPBP4	0.847996907	0.522878745
NCF4	0.847996907	0.48148606
MRPS2	0.847996907	0.455931956
cnot9	0.847996907	0.318758763
SLAMF1	0.847996907	0.318758763
infB	0.847996907	0.301029996
POLR1E	0.847996907	0.26760624
MRPS30	0.847996907	0.259637311
BCKDHA	0.847996907	0.259637311
OAS1	0.847996907	0.244125144
NUP155	0.847996907	0.207608311
ARF6	0.847996907	0.148741651
SEC24C	0.765534746	1.769551079
RPL26	0.765534746	1.721246399
ARPC1B	0.765534746	1.602059991
PPP1CB	0.765534746	1.13667714
ACTG1	0.765534746	0.823908741
GSTO1	0.765534746	0.823908741
RPS24	0.765534746	0.823908741
HSD17B12	0.765534746	0.721246399
ACTC1	0.765534746	0.677780705
CDK6	0.765534746	0.677780705
ZC3HAV1	0.765534746	0.552841969
KIFC1	0.765534746	0.552841969
TAPBP	0.765534746	0.552841969
TBL2	0.765534746	0.537602002
TMOD2	0.765534746	0.468521083
LRRFIP2	0.765534746	0.420216403
ARPC1A	0.765534746	0.420216403
LSM14A	0.765534746	0.387216143
NDUFA10	0.765534746	0.37675071
tuf	0.765534746	0.30980392
CPSF3	0.765534746	0.292429824
GCN1	0.765534746	0.244125144
ETHE1	0.765534746	0.207608311
NDUFB7	0.765534746	0.13667714
DDX46	0.765534746	0.124938737
CCDC97	0.765534746	0.124938737

MAP7D1	0.765534746	0.124938737
ARPC2	0.678071905	1.259637311
PPP1CA	0.678071905	1.055517328
ARPC3	0.678071905	1.017728767
RPL27A	0.678071905	1
RPL28	0.678071905	0.769551079
ARHGEF2	0.678071905	0.744727495
PABPN1	0.678071905	0.677780705
ACTBL2	0.678071905	0.602059991
MYH9	0.678071905	0.537602002
EFHD2	0.678071905	0.508638306
SLC7A5	0.678071905	0.468521083
RFTN1	0.678071905	0.455931956
ZW10	0.678071905	0.431798276
TMOD1	0.678071905	0.366531544
MYO6	0.678071905	0.356547324
CRLF3	0.678071905	0.292429824
GRPEL1	0.678071905	0.27572413
PIN1	0.678071905	0.26760624
SUMF2	0.678071905	0.26760624
BABAM1	0.678071905	0.26760624
Ago2	0.678071905	0.229147988
STX12	0.678071905	0.229147988
QTRT1	0.678071905	0.22184875
ELP3	0.678071905	0.22184875
RPL29	0.584962501	2.214670165
CYTH1	0.584962501	2.187086643
RPL30	0.584962501	1.26760624
RPS8	0.584962501	1.244125144
RPL36A	0.584962501	1.207608311
OAS2	0.584962501	1.15490196
CD48	0.584962501	0.920818754
SNRPD1	0.584962501	0.886056648
PRDX2	0.584962501	0.853871964
SMAP2	0.584962501	0.853871964
BST2	0.584962501	0.795880017
HNRNPH1	0.584962501	0.769551079
SMU1	0.584962501	0.698970004
CSN1S1	0.584962501	0.677780705
GTF2H1	0.584962501	0.677780705
BTLA	0.584962501	0.677780705
ARPC4	0.584962501	0.638272164
HLA-DQB1	0.584962501	0.619788758
RAC2	0.584962501	0.552841969
RBM4	0.584962501	0.552841969
CAPZB	0.584962501	0.494850022
MEF2C	0.584962501	0.494850022

STAU1	0.584962501	0.443697499
SEC61A1	0.584962501	0.431798276
LCK	0.584962501	0.387216143
RPS27	0.584962501	0.387216143
SHPK	0.584962501	0.387216143
DDX3Y	0.584962501	0.366531544
NUP62	0.584962501	0.337242168
LPCAT1	0.584962501	0.30980392
FAM129C	0.584962501	0.301029996
TFB1M	0.584962501	0.301029996
APIP	0.584962501	0.283996656
SSR4	0.584962501	0.26760624
MRPL39	0.584962501	0.26760624
KRT77	0.584962501	0.244125144
SAAL1	0.584962501	0.207608311
DNAJB11	0.584962501	0.207608311
CYC1	0.584962501	0.200659451
KLC1	0.584962501	0.193820026
EMC3	0.584962501	0.173925197
PYCARD	0.584962501	0.173925197
YTHDF3	0.584962501	0.173925197
TP53RK	0.584962501	0.173925197
CBFB	0.584962501	0.173925197
PDCD10	0.584962501	0.119186408
Slc25a5	0.485426827	3.161150909
RPL10	0.485426827	1.552841969
CFL1	0.485426827	1.468521083
TBCB	0.485426827	1.431798276
DDX17	0.485426827	0.920818754
NIFK	0.485426827	0.886056648
PTGES2	0.485426827	0.886056648
SYNGR2	0.485426827	0.886056648
LARP4	0.485426827	0.853871964
RPS7	0.485426827	0.823908741
CAPZA1	0.485426827	0.744727495
DIS3	0.485426827	0.721246399
MYL6	0.485426827	0.698970004
SQOR	0.485426827	0.677780705
CARM1	0.485426827	0.657577319
TRAF1	0.485426827	0.657577319
RBMX	0.485426827	0.619788758
RPS23	0.485426827	0.619788758
GNL3	0.485426827	0.537602002
RAC1	0.485426827	0.537602002
MS4A1	0.485426827	0.508638306
MYL12A	0.485426827	0.494850022
SF3B3	0.485426827	0.494850022

GNAI3	0.485426827	0.468521083
TMOD3	0.485426827	0.455931956
UPF1	0.485426827	0.455931956
RFC4	0.485426827	0.420216403
DHX36	0.485426827	0.420216403
SPATS2	0.485426827	0.387216143
RPL18A	0.485426827	0.37675071
TOMM70	0.485426827	0.366531544
COPE	0.485426827	0.356547324
FAM98A	0.485426827	0.356547324
UBE2M	0.485426827	0.356547324
FBL	0.485426827	0.318758763
IKZF1	0.485426827	0.30980392
RBM8A	0.485426827	0.292429824
FASN	0.485426827	0.251811973
RAB10	0.485426827	0.244125144
ACSL1	0.485426827	0.236572006
POLR2E	0.485426827	0.22184875
STAU2	0.485426827	0.214670165
DPM1	0.485426827	0.207608311
CD38	0.485426827	0.207608311
DERA	0.485426827	0.173925197
Cnot2	0.485426827	0.167491087
SMAP	0.485426827	0.167491087
GSK3B	0.485426827	0.161150909
ARMCX3	0.485426827	0.161150909
SHOC2	0.485426827	0.161150909
FAM91A1	0.485426827	0.161150909
PLEKHO2	0.485426827	0.161150909
PURB	0.485426827	0.161150909
EMC8	0.485426827	0.161150909
DCP1A	0.485426827	0.161150909
CTSC	0.485426827	0.161150909
PUS7	0.485426827	0.148741651
TRIM26	0.485426827	0.148741651
PPP4R2	0.485426827	0.148741651
AHNAK	0.485426827	0.148741651
PAPSS1	0.485426827	0.148741651
RWDD1	0.485426827	0.148741651
DPY30	0.485426827	0.148741651
FCHSD2	0.485426827	0.148741651
MTPN	0.485426827	0.148741651
SH3BP1	0.485426827	0.13076828
MRPS35	0.485426827	0.124938737
RAB21	0.485426827	0.124938737
CTSD	0.485426827	0.124938737
HSD17B8	0.485426827	0.119186408

CDK5	0.485426827	0.113509275
UBTF	0.485426827	0.102372909
REPS1	0.485426827	0.075720714
SLC25A4	0.378511623	1.721246399
SRP14	0.378511623	1.397940009
RPL3	0.378511623	1.387216143
RPS14	0.378511623	1.096910013
PTMA	0.378511623	1.080921908
CHTOP	0.378511623	0.886056648
RPL17	0.378511623	0.795880017
RPS11	0.378511623	0.795880017
DBNL	0.378511623	0.698970004
ACTL6A	0.378511623	0.677780705
ANKRD13A	0.378511623	0.657577319
MRPL28	0.378511623	0.657577319
LSM7	0.378511623	0.657577319
RTRAF	0.378511623	0.638272164
YME1L1	0.378511623	0.638272164
TPM4	0.378511623	0.552841969
PSMB2	0.378511623	0.552841969
BAG2	0.378511623	0.552841969
ISG20	0.378511623	0.552841969
B4GALT1	0.378511623	0.552841969
GPCPD1	0.378511623	0.552841969
COPS7B	0.378511623	0.537602002
TFRC	0.378511623	0.494850022
NUFIP2	0.378511623	0.494850022
PGAM1	0.378511623	0.48148606
RPL34	0.378511623	0.48148606
TSG101	0.378511623	0.443697499
CALM1	0.378511623	0.431798276
CNPY2	0.378511623	0.420216403
ap2m1a	0.378511623	0.420216403
MMAB	0.378511623	0.420216403
PABPC4	0.378511623	0.397940009
RBBP7	0.378511623	0.397940009
MRPL11	0.378511623	0.397940009
LGALS9	0.378511623	0.397940009
RPL7A	0.378511623	0.387216143
PPP2R2A	0.378511623	0.387216143
IGF2BP3	0.378511623	0.356547324
RTCB	0.378511623	0.356547324
ACAT1	0.378511623	0.346787486
COPS5	0.378511623	0.346787486
RPL22	0.378511623	0.327902142
IKZF3	0.378511623	0.30980392
GRSF1	0.378511623	0.30980392

MOV10	0.378511623	0.30980392
MYO1E	0.378511623	0.301029996
SMC2	0.378511623	0.301029996
IFI16	0.378511623	0.292429824
DDX21	0.378511623	0.27572413
UCHL5	0.378511623	0.27572413
DHX9	0.378511623	0.259637311
UNC45A	0.378511623	0.259637311
PPP1R7	0.378511623	0.259637311
GATAD2A	0.378511623	0.259637311
OGT	0.378511623	0.259637311
MYL6B	0.378511623	0.244125144
ABRAXAS2	0.378511623	0.236572006
FXR1	0.378511623	0.229147988
NUP50	0.378511623	0.22184875
HPRT1	0.378511623	0.22184875
NAT10	0.378511623	0.207608311
SNRPA1	0.378511623	0.200659451
IDE	0.378511623	0.200659451
SNRPD2	0.378511623	0.187086643
ADD3	0.378511623	0.167491087
NUSAP1	0.378511623	0.161150909
RALA	0.378511623	0.15490196
GEMIN4	0.378511623	0.15490196
COPS6	0.378511623	0.142667504
PPIL2	0.378511623	0.142667504
UBFD1	0.378511623	0.13667714
PRKAG1	0.378511623	0.13076828
CHCHD2	0.378511623	0.124938737
IQGAP1	0.378511623	0.119186408
SLIRP	0.378511623	0.113509275
SH3BGRL	0.378511623	0.113509275
THOC3	0.378511623	0.113509275
ITPA	0.378511623	0.113509275
ARL1	0.378511623	0.113509275
EIF3A	0.378511623	0.107905397
ZC3H14	0.378511623	0.102372909
EARS2	0.378511623	0.102372909
GSS	0.378511623	0.091514981
BLMH	0.378511623	0.091514981
ISOC2	0.378511623	0.091514981
POLD1	0.378511623	0.080921908
POLR2B	0.378511623	0.075720714
ZC3H11A	0.378511623	0.075720714
DDX6	0.263034406	2.795880017
SLC25A6	0.263034406	2.008773924
RPS3A	0.263034406	1.795880017

DNM1L	0.263034406	1.420216403
RPL24	0.263034406	1.283996656
DDX5	0.263034406	0.920818754
DARS	0.263034406	0.886056648
FARSB	0.263034406	0.769551079
CLIC1	0.263034406	0.721246399
BSG	0.263034406	0.721246399
MCM7	0.263034406	0.698970004
CAPG	0.263034406	0.698970004
DHX15	0.263034406	0.619788758
PSMD11	0.263034406	0.619788758
RPS16	0.263034406	0.585026652
PSMD8	0.263034406	0.585026652
OTUB1	0.263034406	0.568636236
HNRNPA1	0.263034406	0.552841969
SSBP1	0.263034406	0.552841969
HIST2H2AC	0.263034406	0.552841969
AK2	0.263034406	0.537602002
GNB1	0.263034406	0.522878745
HNRNPU	0.263034406	0.508638306
RPS6	0.263034406	0.508638306
TPI1	0.263034406	0.494850022
CORO1A	0.263034406	0.494850022
NAP1L1	0.263034406	0.494850022
CSDE1	0.263034406	0.494850022
HLA-DQA1	0.263034406	0.468521083
PABPC1	0.263034406	0.455931956
CYCS	0.263034406	0.455931956
RDX	0.263034406	0.443697499
RPL7	0.263034406	0.431798276
VDAC3	0.263034406	0.420216403
NCL	0.263034406	0.408935393
RPL19	0.263034406	0.408935393
CDK1	0.263034406	0.408935393
PAFAH1B2	0.263034406	0.408935393
HCLS1	0.263034406	0.397940009
RECQL	0.263034406	0.397940009
PCBP2	0.263034406	0.397940009
HIST1H1B	0.263034406	0.387216143
RPL23	0.263034406	0.387216143
BAP18	0.263034406	0.37675071
HLA-DRB4	0.263034406	0.37675071
ABCE1	0.263034406	0.366531544
RPS25	0.263034406	0.366531544
HNRNPA2B1	0.263034406	0.356547324
ACLY	0.263034406	0.356547324
TPM3	0.263034406	0.346787486

IGF2BP1	0.263034406	0.346787486
RPL23A	0.263034406	0.337242168
EIF3L	0.263034406	0.327902142
PDHA1	0.263034406	0.318758763
CDC5L	0.263034406	0.318758763
DDX3X	0.263034406	0.30980392
LSP1	0.263034406	0.30980392
SNAP23	0.263034406	0.292429824
PSMA1	0.263034406	0.283996656
TRIM25	0.263034406	0.283996656
PSMA6	0.263034406	0.27572413
RPL14	0.263034406	0.27572413
NARS	0.263034406	0.27572413
SNRPB	0.263034406	0.27572413
ABCF2	0.263034406	0.26760624
RFC3	0.263034406	0.259637311
RPLP1	0.263034406	0.259637311
G3BP2	0.263034406	0.251811973
KIF5B	0.263034406	0.244125144
KRT2	0.263034406	0.236572006
TOM1	0.263034406	0.236572006
CCDC6	0.263034406	0.236572006
KRT10	0.263034406	0.214670165
RAVER1	0.263034406	0.214670165
CAPRIN1	0.263034406	0.200659451
AGPS	0.263034406	0.200659451
SNRPA	0.263034406	0.187086643
RAB1A	0.263034406	0.187086643
YTHDF2	0.263034406	0.180456064
BCAP31	0.263034406	0.180456064
MNDA	0.263034406	0.167491087
PSMD5	0.263034406	0.167491087
RUNX3	0.263034406	0.161150909
RAB7A	0.263034406	0.15490196
USP10	0.263034406	0.15490196
FXR2	0.263034406	0.15490196
ARMC6	0.263034406	0.15490196
TSFM	0.263034406	0.148741651
RBBP4	0.263034406	0.148741651
ELOA	0.263034406	0.142667504
ILKAP	0.263034406	0.142667504
AAMP	0.263034406	0.142667504
NUBP2	0.263034406	0.142667504
FAM50A	0.263034406	0.142667504
RBM14	0.263034406	0.13667714
TES	0.263034406	0.13667714
DNAJC2	0.263034406	0.124938737

CYP51A1	0.263034406	0.124938737
SNX1	0.263034406	0.119186408
GM2A	0.263034406	0.119186408
BAX	0.263034406	0.113509275
LGALS1	0.263034406	0.113509275
NDUFV2	0.263034406	0.113509275
ECI1	0.263034406	0.113509275
TRNT1	0.263034406	0.096910013
NUDT4	0.263034406	0.096910013
SEPHS1	0.263034406	0.096910013
CPSF2	0.263034406	0.096910013
RUFY1	0.263034406	0.091514981
PTGES3	0.263034406	0.091514981
ATP5MF	0.263034406	0.091514981
VAPB	0.263034406	0.086186148
SRRM2	0.263034406	0.080921908
NDUFB10	0.263034406	0.080921908
SSSCA1	0.263034406	0.080921908
PLCG2	0.263034406	0.075720714
FMR1	0.263034406	0.075720714
SLC25A10	0.263034406	0.070581074
SEC23IP	0.263034406	0.070581074
ATP13A1	0.263034406	0.065501549
TAGLN2	0.137503524	1.031517051
PFKL	0.137503524	0.677780705
PRPF19	0.137503524	0.585026652
VDAC2	0.137503524	0.568636236
HNRNPD	0.137503524	0.431798276
RPL6	0.137503524	0.420216403
ANXA1	0.137503524	0.356547324
SLC3A2	0.137503524	0.356547324
PTBP1	0.137503524	0.346787486
STRBP	0.137503524	0.346787486
PSME2	0.137503524	0.337242168
HADHA	0.137503524	0.327902142
RPS3	0.137503524	0.318758763
EEF1A1	0.137503524	0.301029996
EEF1G	0.137503524	0.301029996
HIST1H1D	0.137503524	0.301029996
RPL27	0.137503524	0.283996656
EIF3E	0.137503524	0.27572413
DRG1	0.137503524	0.27572413
CTBP1	0.137503524	0.26760624
RCC2	0.137503524	0.259637311
Gnas	0.137503524	0.259637311
PHB	0.137503524	0.251811973
CTPS1	0.137503524	0.251811973

STX7	0.137503524	0.251811973
FDFT1	0.137503524	0.251811973
AAAS	0.137503524	0.251811973
HNRNPL	0.137503524	0.244125144
IMMT	0.137503524	0.244125144
VAPA	0.137503524	0.244125144
PYM1	0.137503524	0.236572006
RPL13	0.137503524	0.214670165
IDH3A	0.137503524	0.214670165
PPP2CA	0.137503524	0.214670165
RASAL3	0.137503524	0.207608311
SDHA	0.137503524	0.207608311
GPS1	0.137503524	0.200659451
ABCF1	0.137503524	0.193820026
CD99	0.137503524	0.193820026
RPL10A	0.137503524	0.187086643
SNW1	0.137503524	0.187086643
NSUN5	0.137503524	0.187086643
DDX1	0.137503524	0.180456064
HNRNPR	0.137503524	0.180456064
MRPS22	0.137503524	0.173925197
XRCC1	0.137503524	0.167491087
EMD	0.137503524	0.167491087
STOML2	0.137503524	0.161150909
EIF2S1	0.137503524	0.161150909
RPS17	0.137503524	0.15490196
LYAR	0.137503524	0.15490196
RPS2	0.137503524	0.148741651
NONO	0.137503524	0.142667504
KPNB1	0.137503524	0.142667504
RPS5	0.137503524	0.142667504
DNAJA3	0.137503524	0.142667504
C8orf33	0.137503524	0.142667504
PARP1	0.137503524	0.13667714
EIF3M	0.137503524	0.13667714
RPS20	0.137503524	0.13076828
HIST1H1C	0.137503524	0.124938737
MSH2	0.137503524	0.124938737
PSMB10	0.137503524	0.124938737
ILVBL	0.137503524	0.124938737
SLC16A1	0.137503524	0.124938737
COLGALT1	0.137503524	0.124938737
SNRPD3	0.137503524	0.124938737
TARS	0.137503524	0.119186408
PGD	0.137503524	0.119186408
FAM120A	0.137503524	0.119186408
GPX1	0.137503524	0.119186408

MMS19	0.137503524	0.119186408
MAT2A	0.137503524	0.113509275
ARHGEF1	0.137503524	0.113509275
PLG	0.137503524	0.113509275
ISG15	0.137503524	0.113509275
G3BP1	0.137503524	0.107905397
PIH1D1	0.137503524	0.102372909
MIPEP	0.137503524	0.102372909
TRAFD1	0.137503524	0.102372909
HNRNPA0	0.137503524	0.096910013
KRT14	0.137503524	0.091514981
NME2	0.137503524	0.091514981
MRTO4	0.137503524	0.091514981
PFDN2	0.137503524	0.091514981
SH3GL1	0.137503524	0.091514981
IKBIP	0.137503524	0.091514981
SORD	0.137503524	0.091514981
HNRNPF	0.137503524	0.086186148
SFPQ	0.137503524	0.080921908
RPL8	0.137503524	0.080921908
NT5C2	0.137503524	0.080921908
TBL1XR1	0.137503524	0.080921908
LBR	0.137503524	0.080921908
C1QBP	0.137503524	0.075720714
SYK	0.137503524	0.075720714
MAN2B1	0.137503524	0.075720714
LARP4B	0.137503524	0.075720714
PAWR	0.137503524	0.075720714
EHD1	0.137503524	0.070581074
BUB3	0.137503524	0.070581074
UBE2I	0.137503524	0.070581074
LAT2	0.137503524	0.070581074
PGRMC2	0.137503524	0.070581074
ERLIN2	0.137503524	0.070581074
Igkc	0.137503524	0.065501549
PRDX3	0.137503524	0.065501549
ARFGAP2	0.137503524	0.065501549
COPA	0.137503524	0.060480747
GMIP	0.137503524	0.060480747
FAM107B	0.137503524	0.055517328
ASNA1	0.137503524	0.055517328
INPP5D	0.137503524	0.055517328
HGH1	0.137503524	0.055517328
TMED9	0.137503524	0.050609993
TYMS	0.137503524	0.050609993
RABEP1	0.137503524	0.050609993
COIL	0.137503524	0.050609993

ADSL	0.137503524	0.045757491
ELF1	0.137503524	0.045757491
PSAP	0.137503524	0.045757491
PIK3AP1	0.137503524	0.045757491
CDK5RAP3	0.137503524	0.040958608
DNAAF5	0.137503524	0.040958608
HDHD5	0.137503524	0.040958608
Gapdh	0.137503524	0.040958608
GLO1	0.137503524	0.036212173
SRPK1	0.137503524	0.036212173
PDS5A	0.137503524	0.036212173
XPO7	0.137503524	0.031517051
PPP6R1	0.137503524	0.031517051
HSPBP1	0.137503524	0.031517051
NOP53	0.137503524	0.026872146
KRT4	0	0.920818754
UBL4A	0	0.823908741
DDX27	0	0.823908741
FAH	0	0.823908741
GRK3	0	0.823908741
FGD2	0	0.431798276
OSGEP	0	0.431798276
GRN	0	0.366531544
TNPO1	0	0.318758763
LDHB	0	0.27572413
TMPO	0	0.27572413
AARS	0	0.259637311
STAT1	0	0.251811973
MCM4	0	0.251811973
PRMT1	0	0.180456064
EIF2S3	0	0.173925197
ILF3	0	0.167491087
ATP5F1B	0	0.148741651
FKBP4	0	0.148741651
MTDH	0	0.148741651
HNRNPC	0	0.148741651
EEF1D	0	0.142667504
RPLP0	0	0.13076828
PCBP1	0	0.13076828
PGM1	0	0.13076828
SNRPB2	0	0.13076828
AHCY	0	0.124938737
DNAJA2	0	0.119186408
TUBB	0	0.113509275
YWHAZ	0	0.113509275
HSPA1A	0	0.113509275
PPIA	0	0.107905397

HIST1H2BK	0	0.107905397
HSPA9	0	0.102372909
PAK2	0	0.102372909
RpS9	0	0.102372909
RPS10	0	0.096910013
PPA2	0	0.096910013
TBCA	0	0.096910013
ENO1	0	0.091514981
NPM1	0	0.086186148
RPL5	0	0.086186148
PCNP	0	0.086186148
TRIM22	0	0.086186148
TKT	0	0.080921908
ANXA2	0	0.080921908
MDH2	0	0.080921908
NACA	0	0.080921908
HSPA14	0	0.080921908
CYB5R3	0	0.080921908
PPT1	0	0.080921908
SYNCRIP	0	0.075720714
RPL12	0	0.070581074
ETFA	0	0.070581074
EIF3H	0	0.065501549
XRCC5	0	0.060480747
RPL4	0	0.060480747
Phb2	0	0.060480747
PAICS	0	0.060480747
FARSA	0	0.060480747
CCAR2	0	0.060480747
GLS	0	0.060480747
CDC42	0	0.060480747
PCK2	0	0.060480747
PA2G4	0	0.055517328
RPS13	0	0.055517328
TOP1	0	0.055517328
ASNS	0	0.055517328
YWHAQ	0	0.055517328
RAB11B	0	0.055517328
PSMB3	0	0.055517328
TUBB3	0	0.055517328
GAPDH	0	0.050609993
RPS4X	0	0.050609993
EIF2AK2	0	0.050609993
GRWD1	0	0.050609993
TUBB4B	0	0.050609993
ELMO1	0	0.050609993
PRDX6	0	0.050609993

PSMA5	0	0.050609993
TUBB6	0	0.050609993
ADH5	0	0.050609993
TCP1	0	0.045757491
GDI2	0	0.045757491
PSMC1	0	0.045757491
ERP29	0	0.045757491
PSMC5	0	0.045757491
DDX19A	0	0.045757491
PUS1	0	0.045757491
TMX1	0	0.045757491
MSN	0	0.040958608
PDIA3	0	0.040958608
PARK7	0	0.040958608
HSD17B10	0	0.040958608
PSMA4	0	0.040958608
KRT5	0	0.040958608
AKR1A1	0	0.040958608
PRMT5	0	0.040958608
ZC3H15	0	0.040958608
KIF2C	0	0.040958608
CASP3	0	0.040958608
PTPN6	0	0.036212173
HNRNPM	0	0.036212173
EIF3D	0	0.036212173
SF3A3	0	0.036212173
RPL18	0	0.036212173
IRF4	0	0.036212173
HMGCS1	0	0.036212173
EPS15L1	0	0.036212173
CAMK4	0	0.036212173
MOGS	0	0.036212173
MYD88	0	0.036212173
HMGB1	0	0.031517051
SSB	0	0.031517051
DNAJA1	0	0.031517051
UQCRC1	0	0.031517051
FDPS	0	0.031517051
RABGGTA	0	0.031517051
PPP6C	0	0.031517051
NCAPH	0	0.031517051
AKR7A2	0	0.031517051
FCRLA	0	0.031517051
ANXA5	0	0.026872146
UQCRC2	0	0.026872146
ST13	0	0.026872146
ETF1	0	0.026872146

TBK1	0	0.026872146
RPL11	0	0.026872146
RPS12	0	0.026872146
ARHGAP18	0	0.026872146
LDAH	0	0.026872146
FAS	0	0.026872146
vps29	0	0.026872146
RIC8A	0	0.026872146
PPP4C	0	0.026872146
GCSAM	0	0.026872146
DNAJB6	0	0.026872146
PCNA	0	0.022276395
TALDO1	0	0.022276395
EVL	0	0.022276395
LASP1	0	0.022276395
AHSA1	0	0.022276395
RPS15A	0	0.022276395
TPD52L2	0	0.022276395
TSR1	0	0.022276395
COTL1	0	0.022276395
OCIAD1	0	0.022276395
Hdac2	0	0.022276395
PFDN5	0	0.022276395
XRCC6	0	0.017728767
CCT4	0	0.017728767
VDAC1	0	0.017728767
ACO2	0	0.017728767
SCFD1	0	0.017728767
CORO7	0	0.017728767
CBR1	0	0.017728767
EEF1B2	0	0.017728767
CHCHD3	0	0.017728767
NFS1	0	0.017728767
SEC31A	0	0.017728767
MCTS1	0	0.017728767
ASF1A	0	0.017728767
KRT1	0	0.013228266
HNRNPK	0	0.013228266
ILF2	0	0.013228266
DLD	0	0.013228266
RAN	0	0.013228266
BTF3	0	0.013228266
MATR3	0	0.013228266
HIST1H4A	0	0.013228266
HK2	0	0.013228266
RRM2	0	0.013228266
KCMF1	0	0.013228266

PKN1	0	0.013228266
EIF2B2	0	0.013228266
EIF4A1	0	0.008773924
GARS	0	0.008773924
VASP	0	0.008773924
RPL9	0	0.008773924
PSMA3	0	0.008773924
ALB	0	0.008773924
LETM1	0	0.008773924
RPL38	0	0.008773924
PEPD	0	0.008773924
RAB5C	0	0.008773924
RFC2	0	0.008773924
UBA5	0	0.008773924
PSMG1	0	0.008773924
HVCN1	0	0.008773924
H1FX	0	0.008773924
KRT16	0	0.008773924
COPS7A	0	0.008773924
PATL1	0	0.008773924
PPIH	0	0.008773924
SPTLC1	0	0.008773924
SELL	0	0.008773924
COX6B1	0	0.008773924
POLRMT	0	0.008773924
LIMD1	0	0.008773924
NUCKS1	0	0.008773924
EZR	0	0.004364805
CS	0	0.004364805
HLA-DRA	0	0.004364805
RPS18	0	0.004364805
RPS27A	0	0.004364805
SLC25A12	0	0.004364805
NAP1L4	0	0.004364805
DNAJB1	0	0.004364805
FDXR	0	0.004364805
AKR1B1	0	0.004364805
EIF2B4	0	0.004364805
CDKN2AIP	0	0.004364805
NUDCD3	0	0.004364805
YY1	0	0.004364805
SEL1L	0	0.004364805
TSC22D4	0	0.004364805
COPS8	0	0.004364805
UAP1	0	0.004364805
CD82	0	0.004364805
NUP37	0	0.004364805

KRT9	0	0
SGTA	0	0
cugbp1-a	0	0
MTHFD1	-0.152003093	1.420216403
CCT5	-0.152003093	1.13667714
CCT8	-0.152003093	1.055517328
LCP1	-0.152003093	1.017728767
IMPDH2	-0.152003093	1
PGK1	-0.152003093	0.886056648
GRHPR	-0.152003093	0.853871964
SH3GLB1	-0.152003093	0.853871964
PPP3CB	-0.152003093	0.853871964
sucC	-0.152003093	0.853871964
PDXK	-0.152003093	0.853871964
ATP5F1D	-0.152003093	0.853871964
HSP90AB1	-0.152003093	0.823908741
YWHAG	-0.152003093	0.744727495
PSME1	-0.152003093	0.721246399
EEF2	-0.152003093	0.698970004
MX1	-0.152003093	0.677780705
LAP3	-0.152003093	0.657577319
PPP2R1A	-0.152003093	0.657577319
PDLIM1	-0.152003093	0.657577319
HSP90AA1	-0.152003093	0.638272164
TUFM	-0.152003093	0.619788758
ANP32A	-0.152003093	0.619788758
PLS3	-0.152003093	0.619788758
SAE1	-0.152003093	0.602059991
RRM1	-0.152003093	0.585026652
FERMT3	-0.152003093	0.568636236
TRIR	-0.152003093	0.552841969
CKAP4	-0.152003093	0.522878745
PDCD4	-0.152003093	0.508638306
GNA13	-0.152003093	0.508638306
WARS	-0.152003093	0.494850022
HLA-B	-0.152003093	0.48148606
PSPC1	-0.152003093	0.48148606
PIP4K2A	-0.152003093	0.468521083
TOMM40	-0.152003093	0.455931956
YBX1	-0.152003093	0.443697499
ANP32B	-0.152003093	0.431798276
LDHA	-0.152003093	0.420216403
RACK1	-0.152003093	0.420216403
HSPA8	-0.152003093	0.408935393
DLST	-0.152003093	0.387216143
PRDX1	-0.152003093	0.366531544
MARCKS	-0.152003093	0.356547324

ARCN1	-0.152003093	0.356547324
PEBP1	-0.152003093	0.356547324
LRRC59	-0.152003093	0.356547324
RARS	-0.152003093	0.346787486
ANXA6	-0.152003093	0.337242168
ALDOA	-0.152003093	0.337242168
SHMT2	-0.152003093	0.337242168
UBA1	-0.152003093	0.327902142
SERBP1	-0.152003093	0.327902142
USP5	-0.152003093	0.327902142
NAMPT	-0.152003093	0.327902142
CCT2	-0.152003093	0.318758763
CAP1	-0.152003093	0.318758763
MCM5	-0.152003093	0.318758763
HSPA5	-0.152003093	0.30980392
RPL21	-0.152003093	0.30980392
ATP5PO	-0.152003093	0.30980392
ATP5F1A	-0.152003093	0.301029996
RPN1	-0.152003093	0.301029996
ME2	-0.152003093	0.301029996
SUCLG2	-0.152003093	0.301029996
PFKP	-0.152003093	0.27572413
SRM	-0.152003093	0.27572413
AP2A1	-0.152003093	0.26760624
PPAT	-0.152003093	0.259637311
RNH1	-0.152003093	0.251811973
RAB35	-0.152003093	0.251811973
KARS	-0.152003093	0.244125144
CDC37	-0.152003093	0.244125144
CCT6A	-0.152003093	0.236572006
AHSG	-0.152003093	0.236572006
EIF3F	-0.152003093	0.236572006
LRRC47	-0.152003093	0.236572006
KIF2A	-0.152003093	0.229147988
NSUN2	-0.152003093	0.229147988
DENR	-0.152003093	0.229147988
HSPA4L	-0.152003093	0.229147988
KHSRP	-0.152003093	0.22184875
PDIA6	-0.152003093	0.22184875
LONP1	-0.152003093	0.22184875
MCMBP	-0.152003093	0.22184875
ADRM1	-0.152003093	0.22184875
EHD4	-0.152003093	0.214670165
PLIN3	-0.152003093	0.214670165
GSK3A	-0.152003093	0.214670165
PGM2	-0.152003093	0.214670165
STAT2	-0.152003093	0.214670165

ACP1	-0.152003093	0.207608311
DPP9	-0.152003093	0.207608311
FKBP1A	-0.152003093	0.207608311
EFTUD2	-0.152003093	0.200659451
TUBA1B	-0.152003093	0.193820026
RUVBL2	-0.152003093	0.193820026
HLA-DRB1	-0.152003093	0.193820026
OGDH	-0.152003093	0.193820026
SKP1	-0.152003093	0.193820026
PNP	-0.152003093	0.187086643
APEX1	-0.152003093	0.187086643
YWHAB	-0.152003093	0.187086643
USO1	-0.152003093	0.187086643
LMAN1	-0.152003093	0.187086643
ACBD3	-0.152003093	0.187086643
TIA1	-0.152003093	0.187086643
YARS	-0.152003093	0.180456064
MCM2	-0.152003093	0.180456064
CPNE3	-0.152003093	0.180456064
PSMA7	-0.152003093	0.180456064
TUBA4A	-0.152003093	0.180456064
COPG2	-0.152003093	0.180456064
UBQLN2	-0.152003093	0.180456064
PHGDH	-0.152003093	0.173925197
TIMM44	-0.152003093	0.173925197
ACSL3	-0.152003093	0.173925197
OGFR	-0.152003093	0.173925197
NOSIP	-0.152003093	0.173925197
SAR1A	-0.152003093	0.173925197
PPIB	-0.152003093	0.167491087
MRI1	-0.152003093	0.167491087
OSBPL8	-0.152003093	0.167491087
NELFB	-0.152003093	0.167491087
SEC13	-0.152003093	0.167491087
CNN2	-0.152003093	0.161150909
ITGB7	-0.152003093	0.161150909
GSPT1	-0.152003093	0.161150909
LAMP2	-0.152003093	0.161150909
ALDH18A1	-0.152003093	0.15490196
NME1	-0.152003093	0.15490196
SRSF1	-0.152003093	0.15490196
FH	-0.152003093	0.15490196
PSMD4	-0.152003093	0.15490196
DDOST	-0.152003093	0.15490196
HSPD1	-0.152003093	0.148741651
QARS	-0.152003093	0.148741651
AFG3L2	-0.152003093	0.148741651

COX4I1	-0.152003093	0.148741651
BCAT2	-0.152003093	0.148741651
PLRG1	-0.152003093	0.148741651
GATM	-0.152003093	0.148741651
TRMT1	-0.152003093	0.148741651
EIF4E	-0.152003093	0.148741651
pstS	-0.152003093	0.148741651
NUDC	-0.152003093	0.142667504
AP1M1	-0.152003093	0.142667504
CLPP	-0.152003093	0.142667504
IFI44L	-0.152003093	0.13667714
NAA10	-0.152003093	0.13667714
ACTR1A	-0.152003093	0.13667714
ATP1B3	-0.152003093	0.13667714
GLYR1	-0.152003093	0.13667714
CPT1A	-0.152003093	0.13667714
PCID2	-0.152003093	0.13667714
OXCT1	-0.152003093	0.13076828
TRABD	-0.152003093	0.13076828
GANAB	-0.152003093	0.124938737
PSMC6	-0.152003093	0.124938737
SF3B2	-0.152003093	0.124938737
XPOT	-0.152003093	0.124938737
TAF15	-0.152003093	0.124938737
AIFM1	-0.152003093	0.119186408
CSK	-0.152003093	0.119186408
MAPRE1	-0.152003093	0.119186408
IRF2BP2	-0.152003093	0.119186408
SSR1	-0.152003093	0.119186408
UBLCP1	-0.152003093	0.119186408
CCT3	-0.152003093	0.113509275
HNRNPDL	-0.152003093	0.113509275
CANX	-0.152003093	0.107905397
BTK	-0.152003093	0.107905397
SF3A2	-0.152003093	0.107905397
CD3EAP	-0.152003093	0.107905397
ZNF207	-0.152003093	0.107905397
SIT1	-0.152003093	0.107905397
GSTP1	-0.152003093	0.102372909
GLRX3	-0.152003093	0.102372909
CD97	-0.152003093	0.102372909
LIG1	-0.152003093	0.102372909
UBE2N	-0.152003093	0.102372909
GGH	-0.152003093	0.102372909
EIF3B	-0.152003093	0.096910013
HMGB2	-0.152003093	0.096910013
PRPS1	-0.152003093	0.096910013

RTN4	-0.152003093	0.096910013
COPS4	-0.152003093	0.096910013
PCMT1	-0.152003093	0.096910013
PSMB4	-0.152003093	0.096910013
ABCF3	-0.152003093	0.096910013
RPS26	-0.152003093	0.096910013
CHMP2A	-0.152003093	0.096910013
LRPPRC	-0.152003093	0.091514981
MRPL12	-0.152003093	0.091514981
XRN2	-0.152003093	0.091514981
GLOD4	-0.152003093	0.091514981
EPS15	-0.152003093	0.091514981
NCBP1	-0.152003093	0.091514981
CLPX	-0.152003093	0.091514981
DNAJC7	-0.152003093	0.086186148
PARP9	-0.152003093	0.086186148
NAGK	-0.152003093	0.086186148
CLTA	-0.152003093	0.086186148
Lyar	-0.152003093	0.086186148
RPL32	-0.152003093	0.080921908
TECR	-0.152003093	0.080921908
CUL1	-0.152003093	0.080921908
HIBCH	-0.152003093	0.080921908
PSMG2	-0.152003093	0.080921908
RBM17	-0.152003093	0.080921908
POLD3	-0.152003093	0.080921908
AIMP2	-0.152003093	0.075720714
DAP3	-0.152003093	0.075720714
PRDX5	-0.152003093	0.075720714
PSMD13	-0.152003093	0.070581074
WAS	-0.152003093	0.070581074
DNAJC9	-0.152003093	0.070581074
EXOSC6	-0.152003093	0.070581074
LTV1	-0.152003093	0.070581074
PNKP	-0.152003093	0.070581074
HSD17B4	-0.152003093	0.065501549
RAB8A	-0.152003093	0.065501549
STAT3	-0.152003093	0.065501549
DDX54	-0.152003093	0.065501549
ATP5PD	-0.152003093	0.060480747
FKBP3	-0.152003093	0.060480747
TUBG1	-0.152003093	0.060480747
AGK	-0.152003093	0.060480747
PTPRCAP	-0.152003093	0.060480747
MIF	-0.152003093	0.060480747
SERPINH1	-0.152003093	0.060480747
Grcc10	-0.152003093	0.060480747

NEDD1	-0.152003093	0.060480747
CMTR1	-0.152003093	0.060480747
CSTF3	-0.152003093	0.060480747
PAPOLA	-0.152003093	0.060480747
SPCS2	-0.152003093	0.060480747
LSM12	-0.152003093	0.060480747
Babam2	-0.152003093	0.060480747
BUD23	-0.152003093	0.060480747
VCL	-0.152003093	0.055517328
NAPA	-0.152003093	0.055517328
P4HA1	-0.152003093	0.055517328
OPA1	-0.152003093	0.055517328
FAF1	-0.152003093	0.055517328
PICALM	-0.152003093	0.055517328
SASH3	-0.152003093	0.050609993
BLVRA	-0.152003093	0.050609993
ELOB	-0.152003093	0.050609993
TBC1D5	-0.152003093	0.050609993
SH3BGRL3	-0.152003093	0.050609993
AARS2	-0.152003093	0.050609993
F11R	-0.152003093	0.050609993
FAM129A	-0.152003093	0.050609993
WASF2	-0.152003093	0.045757491
DIMT1	-0.152003093	0.045757491
POLD2	-0.152003093	0.045757491
CBX3	-0.152003093	0.045757491
PYGB	-0.152003093	0.045757491
MAPRE2	-0.152003093	0.045757491
ICAM3	-0.152003093	0.045757491
ITGA4	-0.152003093	0.045757491
TLE3	-0.152003093	0.045757491
CCAR1	-0.152003093	0.045757491
IDH3G	-0.152003093	0.045757491
DOK2	-0.152003093	0.045757491
GMPR2	-0.152003093	0.045757491
PLAA	-0.152003093	0.040958608
DHODH	-0.152003093	0.040958608
CAMK2A	-0.152003093	0.040958608
CWF19L1	-0.152003093	0.040958608
IGBP1	-0.152003093	0.036212173
PCYT1A	-0.152003093	0.036212173
TOMM34	-0.152003093	0.036212173
TRAF3IP3	-0.152003093	0.036212173
CNBP	-0.152003093	0.036212173
ZNF428	-0.152003093	0.036212173
AK6	-0.152003093	0.036212173
SUPT5H	-0.152003093	0.036212173

BDH1	-0.152003093	0.036212173
NAF1	-0.152003093	0.036212173
TTLL12	-0.152003093	0.031517051
MRPL44	-0.152003093	0.031517051
PYCR2	-0.152003093	0.031517051
SBDS	-0.152003093	0.031517051
RNASEH2B	-0.152003093	0.031517051
RNPS1	-0.152003093	0.031517051
DIABLO	-0.152003093	0.031517051
MYO1F	-0.152003093	0.031517051
SAMM50	-0.152003093	0.031517051
HTATSF1	-0.152003093	0.031517051
MRPL22	-0.152003093	0.031517051
QDPR	-0.152003093	0.031517051
VPS51	-0.152003093	0.031517051
RPRD1B	-0.152003093	0.026872146
SMG9	-0.152003093	0.026872146
EPN1	-0.152003093	0.026872146
PPM1A	-0.152003093	0.026872146
IDI1	-0.152003093	0.026872146
ERAL1	-0.152003093	0.026872146
ATP6V1D	-0.152003093	0.026872146
nuoB	-0.152003093	0.026872146
RCN1	-0.152003093	0.022276395
CASP1	-0.152003093	0.022276395
RELA	-0.152003093	0.022276395
LAMP1	-0.152003093	0.022276395
NRDC	-0.152003093	0.022276395
GALNT7	-0.152003093	0.022276395
NAA50	-0.152003093	0.022276395
CCDC59	-0.152003093	0.022276395
GOLPH3	-0.152003093	0.022276395
APOL3	-0.152003093	0.022276395
TXLNG	-0.152003093	0.022276395
HSP90B1	-0.321928095	2.060480747
EIF4A3	-0.321928095	1.823908741
ATP5F1C	-0.321928095	1.677780705
VCP	-0.321928095	1.468521083
MCM3	-0.321928095	1.397940009
STRAP	-0.321928095	1.318758763
RPS9	-0.321928095	1.30980392
YBX3	-0.321928095	1.251811973
COPG1	-0.321928095	1.229147988
RANBP3	-0.321928095	1.113509275
P4HB	-0.321928095	0.958607315
RUVBL1	-0.321928095	0.920818754
CAPN1	-0.321928095	0.920818754

MDH1	-0.321928095	0.886056648
TIGAR	-0.321928095	0.886056648
CALR	-0.321928095	0.823908741
BID	-0.321928095	0.823908741
HSPH1	-0.321928095	0.769551079
EIF3I	-0.321928095	0.769551079
SLC25A3	-0.321928095	0.769551079
YWHAE	-0.321928095	0.744727495
NDUFS1	-0.321928095	0.744727495
TAP1	-0.321928095	0.744727495
MTA2	-0.321928095	0.721246399
IL16	-0.321928095	0.721246399
ISOC1	-0.321928095	0.721246399
HLA-A	-0.321928095	0.698970004
HLA-DRB1	-0.321928095	0.677780705
NANS	-0.321928095	0.677780705
TMPO	-0.321928095	0.657577319
RPSA	-0.321928095	0.657577319
PUF60	-0.321928095	0.657577319
DCTN2	-0.321928095	0.657577319
MRPL4	-0.321928095	0.657577319
ACAD9	-0.321928095	0.638272164
DLAT	-0.321928095	0.638272164
TRMT10C	-0.321928095	0.638272164
CNPY3	-0.321928095	0.638272164
APOL2	-0.321928095	0.638272164
EIF5A	-0.321928095	0.619788758
YWHAH	-0.321928095	0.619788758
APBB1IP	-0.321928095	0.619788758
PRPF31	-0.321928095	0.602059991
IST1	-0.321928095	0.602059991
TRIM28	-0.321928095	0.585026652
EIF4G2	-0.321928095	0.585026652
POU2F1	-0.321928095	0.585026652
HLA-C	-0.321928095	0.585026652
TCEA1	-0.321928095	0.568636236
PGAM5	-0.321928095	0.568636236
TIMM50	-0.321928095	0.568636236
PFN1	-0.321928095	0.552841969
HSPA4	-0.321928095	0.552841969
EIF4B	-0.321928095	0.552841969
NASP	-0.321928095	0.552841969
RPS19	-0.321928095	0.552841969
HLA-A	-0.321928095	0.552841969
DDB1	-0.321928095	0.537602002
KPNA2	-0.321928095	0.537602002
HLA-B	-0.321928095	0.537602002

PSMC3	-0.321928095	0.522878745
PSMC4	-0.321928095	0.522878745
U2AF2	-0.321928095	0.522878745
NT5C3A	-0.321928095	0.522878745
MARCKSL1	-0.321928095	0.522878745
GPI	-0.321928095	0.494850022
CCT7	-0.321928095	0.494850022
VPS35	-0.321928095	0.494850022
NFKB1	-0.321928095	0.494850022
IDH3B	-0.321928095	0.494850022
HNRNPAB	-0.321928095	0.494850022
PSMD1	-0.321928095	0.48148606
Eif4h	-0.321928095	0.48148606
PMPCA	-0.321928095	0.468521083
GRAP2	-0.321928095	0.468521083
ETFB	-0.321928095	0.455931956
UBA2	-0.321928095	0.455931956
Eno1	-0.321928095	0.455931956
SCRN1	-0.321928095	0.455931956
GMFG	-0.321928095	0.455931956
CAND1	-0.321928095	0.443697499
PSAT1	-0.321928095	0.443697499
PRPF6	-0.321928095	0.443697499
AP3B1	-0.321928095	0.443697499
Kpna3	-0.321928095	0.443697499
ATIC	-0.321928095	0.431798276
SET	-0.321928095	0.431798276
AP2B1	-0.321928095	0.431798276
ERP44	-0.321928095	0.431798276
GLUD1	-0.321928095	0.420216403
ITGB2	-0.321928095	0.420216403
NCF1	-0.321928095	0.408935393
FNBP1	-0.321928095	0.397940009
PDIA4	-0.321928095	0.387216143
OLA1	-0.321928095	0.387216143
HDAC1	-0.321928095	0.387216143
RAD23B	-0.321928095	0.37675071
ESYT1	-0.321928095	0.37675071
GTPBP1	-0.321928095	0.37675071
PRKCSH	-0.321928095	0.356547324
ARHGAP17	-0.321928095	0.356547324
RFC5	-0.321928095	0.356547324
SRC	-0.321928095	0.356547324
SARS	-0.321928095	0.346787486
PSMB1	-0.321928095	0.346787486
GTF2F1	-0.321928095	0.346787486
TIAL1	-0.321928095	0.346787486

NOP56	-0.321928095	0.346787486
GMPS	-0.321928095	0.337242168
ANXA4	-0.321928095	0.337242168
CNDP2	-0.321928095	0.337242168
TPT1	-0.321928095	0.337242168
PNO1	-0.321928095	0.337242168
PRKACA	-0.321928095	0.337242168
TSTA3	-0.321928095	0.337242168
ANXA7	-0.321928095	0.327902142
ARHGDIA	-0.321928095	0.327902142
RHOA	-0.321928095	0.327902142
RPA2	-0.321928095	0.327902142
STAT5A	-0.321928095	0.327902142
MAP2K2	-0.321928095	0.327902142
PKM	-0.321928095	0.318758763
GOT2	-0.321928095	0.318758763
ACADVL	-0.321928095	0.318758763
MRPL19	-0.321928095	0.318758763
LUC7L2	-0.321928095	0.30980392
MTHFD1L	-0.321928095	0.30980392
UHRF1	-0.321928095	0.30980392
CAB39	-0.321928095	0.301029996
SFXN1	-0.321928095	0.301029996
FSCN1	-0.321928095	0.301029996
U2AF1L5	-0.321928095	0.301029996
ARHGDIB	-0.321928095	0.292429824
HLA-DPB1	-0.321928095	0.292429824
ATP6V1B2	-0.321928095	0.27572413
SUB1	-0.321928095	0.26760624
RPIA	-0.321928095	0.26760624
ARF3	-0.321928095	0.26760624
DNAJC8	-0.321928095	0.259637311
TPD52	-0.321928095	0.259637311
CHMP5	-0.321928095	0.259637311
STAT6	-0.321928095	0.259637311
MT-CO2	-0.321928095	0.259637311
NDUFS3	-0.321928095	0.259637311
HDGF	-0.321928095	0.251811973
ATXN2L	-0.321928095	0.251811973
HSPE1	-0.321928095	0.251811973
KPNA6	-0.321928095	0.251811973
SRP72	-0.321928095	0.244125144
CD44	-0.321928095	0.244125144
SRP54	-0.321928095	0.244125144
PMPCB	-0.321928095	0.244125144
RPL35	-0.321928095	0.236572006
ACAA2	-0.321928095	0.229147988

YARS2	-0.321928095	0.229147988
CHEK1	-0.321928095	0.229147988
RTCA	-0.321928095	0.229147988
OXA1L	-0.321928095	0.229147988
TGOLN2	-0.321928095	0.229147988
SF1	-0.321928095	0.22184875
CAPNS1	-0.321928095	0.22184875
PAK1	-0.321928095	0.214670165
PPP5C	-0.321928095	0.214670165
TERF2IP	-0.321928095	0.214670165
UBE2V1	-0.321928095	0.214670165
GATAD2B	-0.321928095	0.214670165
SRSF2	-0.321928095	0.207608311
NIT2	-0.321928095	0.207608311
RPL36	-0.321928095	0.207608311
GFPT1	-0.321928095	0.193820026
SEC22B	-0.321928095	0.193820026
MBD3	-0.321928095	0.187086643
CARS2	-0.321928095	0.187086643
DCXR	-0.321928095	0.187086643
RTFDC1	-0.321928095	0.187086643
SUMO2	-0.321928095	0.187086643
SNRPF	-0.321928095	0.187086643
PPM1G	-0.321928095	0.180456064
MAP2K1	-0.321928095	0.180456064
CUL3	-0.321928095	0.180456064
IMPA1	-0.321928095	0.180456064
UBXN7	-0.321928095	0.180456064
KYNU	-0.321928095	0.180456064
PQBP1	-0.321928095	0.180456064
MOB1B	-0.321928095	0.180456064
TFCP2	-0.321928095	0.180456064
GMFB	-0.321928095	0.180456064
NOP2	-0.321928095	0.173925197
SUGT1	-0.321928095	0.173925197
DDX23	-0.321928095	0.173925197
M6PR	-0.321928095	0.173925197
SNAP29	-0.321928095	0.173925197
SPTLC2	-0.321928095	0.173925197
PRDX4	-0.321928095	0.173925197
UBE2L3	-0.321928095	0.167491087
PTPA	-0.321928095	0.167491087
ATP5MG	-0.321928095	0.167491087
SNX6	-0.321928095	0.161150909
RAB8B	-0.321928095	0.161150909
GTF2I	-0.321928095	0.15490196
ARHGAP1	-0.321928095	0.15490196

NBN	-0.321928095	0.15490196
HMGN5	-0.321928095	0.15490196
COPS2	-0.321928095	0.148741651
RAB14	-0.321928095	0.148741651
NDUFA9	-0.321928095	0.142667504
CD5	-0.321928095	0.142667504
SRI	-0.321928095	0.142667504
MAP4K1	-0.321928095	0.142667504
TMSB4X	-0.321928095	0.13667714
JPT2	-0.321928095	0.13667714
CTSZ	-0.321928095	0.13667714
FCER2	-0.321928095	0.13076828
PFAS	-0.321928095	0.13076828
MZB1	-0.321928095	0.13076828
SUCLG1	-0.321928095	0.13076828
SCAMP3	-0.321928095	0.13076828
SEC23A	-0.321928095	0.13076828
PRPS2	-0.321928095	0.13076828
ARF5	-0.321928095	0.13076828
ABI3	-0.321928095	0.13076828
EIF5	-0.321928095	0.124938737
IARS	-0.321928095	0.119186408
ARID3A	-0.321928095	0.119186408
C12orf10	-0.321928095	0.119186408
GTPBP10	-0.321928095	0.119186408
SDHB	-0.321928095	0.113509275
UBAP2	-0.321928095	0.113509275
LUC7L3	-0.321928095	0.113509275
IPO8	-0.321928095	0.113509275
DDX49	-0.321928095	0.113509275
CUL4A	-0.321928095	0.107905397
SUPT16H	-0.321928095	0.102372909
ZCCHC8	-0.321928095	0.091514981
HYOU1	-0.321928095	0.091514981
TPP2	-0.321928095	0.080921908
HK1	-0.321928095	0.080921908
FGA	-0.321928095	0.065501549
THOC6	-0.321928095	0.055517328
SNX3	-0.321928095	0.040958608
FUBP1	-0.514573173	2.346787486
SRP68	-0.514573173	2.229147988
PRKCB	-0.514573173	2.22184875
NAE1	-0.514573173	1.698970004
SARNP	-0.514573173	1.677780705
ATXN10	-0.514573173	1.443697499
PDCD6IP	-0.514573173	1.387216143
AP1B1	-0.514573173	1.292429824

SND1	-0.514573173	1.26760624
ALYREF	-0.514573173	1.259637311
TXNDC5	-0.514573173	1.251811973
MCM6	-0.514573173	1.22184875
GART	-0.514573173	1.207608311
RPS28	-0.514573173	1.207608311
HDGFL2	-0.514573173	1.167491087
EIF3C	-0.514573173	1.13076828
DDX39A	-0.514573173	1.102372909
PRIM2	-0.514573173	1.091514981
SERPINB10	-0.514573173	1.091514981
MTAP	-0.514573173	1.080921908
EIF2A	-0.514573173	1.060480747
FUBP3	-0.514573173	1.060480747
MARK2	-0.514573173	1.055517328
CCDC50	-0.514573173	1.045757491
MLEC	-0.514573173	1.040958608
SSRP1	-0.514573173	1.026872146
PSMD6	-0.514573173	1
PRKAR1A	-0.514573173	1
RPA1	-0.514573173	0.958607315
RANBP1	-0.514573173	0.958607315
PSMB8	-0.514573173	0.958607315
UFL1	-0.514573173	0.958607315
CCDC9	-0.514573173	0.958607315
UQCRFS1	-0.514573173	0.958607315
HADHB	-0.514573173	0.920818754
LRMP	-0.514573173	0.920818754
ZYX	-0.514573173	0.920818754
PSMC2	-0.514573173	0.886056648
RPLP2	-0.514573173	0.886056648
EIF2S2	-0.514573173	0.886056648
SLC25A11	-0.514573173	0.886056648
STIP1	-0.514573173	0.853871964
PSMD2	-0.514573173	0.853871964
PLEK	-0.514573173	0.853871964
PFKM	-0.514573173	0.853871964
PSMD7	-0.514573173	0.853871964
TRAP1	-0.514573173	0.823908741
ESD	-0.514573173	0.823908741
CHMP4A	-0.514573173	0.823908741
SLC1A5	-0.514573173	0.795880017
XPO1	-0.514573173	0.769551079
LMNB1	-0.514573173	0.769551079
ATP6V1A	-0.514573173	0.769551079
KHDRBS1	-0.514573173	0.769551079
COPB1	-0.514573173	0.744727495

RPL35A	-0.514573173	0.744727495
RPS15	-0.514573173	0.721246399
THUMPD1	-0.514573173	0.721246399
RPN2	-0.514573173	0.698970004
GOT1	-0.514573173	0.698970004
AP1G1	-0.514573173	0.698970004
AP3M1	-0.514573173	0.698970004
STK4	-0.514573173	0.677780705
PSMD12	-0.514573173	0.677780705
EWSR1	-0.514573173	0.677780705
STK26	-0.514573173	0.657577319
DPYSL2	-0.514573173	0.638272164
SF3A1	-0.514573173	0.638272164
IFIT1	-0.514573173	0.638272164
PTPN1	-0.514573173	0.638272164
DDX47	-0.514573173	0.619788758
COL4A3BP	-0.514573173	0.619788758
DCPS	-0.514573173	0.619788758
AASDHPPT	-0.514573173	0.619788758
PRPSAP1	-0.514573173	0.619788758
USP15	-0.514573173	0.619788758
IDH2	-0.514573173	0.602059991
EIF3G	-0.514573173	0.602059991
NUDT5	-0.514573173	0.602059991
SRA1	-0.514573173	0.602059991
SNX2	-0.514573173	0.585026652
NCLN	-0.514573173	0.585026652
SRRT	-0.514573173	0.585026652
EIF2B1	-0.514573173	0.585026652
NR3C1	-0.514573173	0.585026652
PRPSAP2	-0.514573173	0.568636236
UBQLN1	-0.514573173	0.552841969
ICAM1	-0.514573173	0.552841969
MTHFD2	-0.514573173	0.552841969
CTTN	-0.514573173	0.552841969
SF3B4	-0.514573173	0.552841969
DUT	-0.514573173	0.537602002
PSMD9	-0.514573173	0.508638306
SUCLA2	-0.514573173	0.508638306
NPEPPS	-0.514573173	0.48148606
NSFL1C	-0.514573173	0.48148606
CPSF7	-0.514573173	0.48148606
STMN1	-0.514573173	0.468521083
CACYBP	-0.514573173	0.468521083
NUDT21	-0.514573173	0.468521083
GRB2	-0.514573173	0.468521083
PSMD3	-0.514573173	0.455931956

PSME3	-0.514573173	0.443697499
CD74	-0.514573173	0.443697499
ACAP1	-0.514573173	0.443697499
ACAT2	-0.514573173	0.431798276
PGLS	-0.514573173	0.431798276
ALDOC	-0.514573173	0.420216403
PDHB	-0.514573173	0.420216403
METAP2	-0.514573173	0.408935393
TXNRD1	-0.514573173	0.408935393
TARDBP	-0.514573173	0.408935393
KPNA1	-0.514573173	0.408935393
CCDC124	-0.514573173	0.397940009
CDK9	-0.514573173	0.397940009
ENTPD1	-0.514573173	0.387216143
CNOT11	-0.514573173	0.387216143
CLIC4	-0.514573173	0.37675071
IFIT3	-0.514573173	0.37675071
PSIP1	-0.514573173	0.37675071
SRSF3	-0.514573173	0.37675071
EIF4A2	-0.514573173	0.37675071
AIMP1	-0.514573173	0.366531544
UMPS	-0.514573173	0.366531544
UBXN1	-0.514573173	0.366531544
PPID	-0.514573173	0.356547324
UPF3B	-0.514573173	0.356547324
PSMB5	-0.514573173	0.356547324
CMPK1	-0.514573173	0.346787486
TUBB2B	-0.514573173	0.346787486
POLR1C	-0.514573173	0.337242168
MAPK1	-0.514573173	0.337242168
COX5A	-0.514573173	0.337242168
CDK2	-0.514573173	0.337242168
JPT1	-0.514573173	0.337242168
PEF1	-0.514573173	0.337242168
HELLS	-0.514573173	0.337242168
ECH1	-0.514573173	0.327902142
CPOX	-0.514573173	0.327902142
SCP2	-0.514573173	0.327902142
IDH1	-0.514573173	0.327902142
DKC1	-0.514573173	0.327902142
PPA1	-0.514573173	0.318758763
BASP1	-0.514573173	0.318758763
COPB2	-0.514573173	0.318758763
UBAP2L	-0.514573173	0.318758763
ECHS1	-0.514573173	0.318758763
HNRNPH3	-0.514573173	0.318758763
FAM49B	-0.514573173	0.30980392

RPS21	-0.514573173	0.30980392
CPNE1	-0.514573173	0.30980392
GBE1	-0.514573173	0.30980392
SMARCE1	-0.514573173	0.30980392
HADH	-0.514573173	0.301029996
LRRFIP1	-0.514573173	0.301029996
ELAC2	-0.514573173	0.301029996
TXN	-0.514573173	0.292429824
B2M	-0.514573173	0.292429824
EIF3J	-0.514573173	0.283996656
Gphn	-0.514573173	0.283996656
VPS37B	-0.514573173	0.283996656
VTA1	-0.514573173	0.283996656
STIM1	-0.514573173	0.27572413
LRRC40	-0.514573173	0.27572413
RPS4Y1	-0.514573173	0.27572413
EML2	-0.514573173	0.27572413
TBRG4	-0.514573173	0.27572413
GATD3B	-0.514573173	0.27572413
GINS4	-0.514573173	0.27572413
RRAGC	-0.514573173	0.27572413
AURKB	-0.514573173	0.27572413
PLEKHF2	-0.514573173	0.27572413
CSNK2A2	-0.514573173	0.27572413
PRPF40A	-0.514573173	0.27572413
DHRS7	-0.514573173	0.27572413
THYN1	-0.514573173	0.27572413
UFC1	-0.514573173	0.27572413
REEP5	-0.514573173	0.27572413
Ifitm3	-0.514573173	0.27572413
CD58	-0.514573173	0.27572413
PFDN1	-0.514573173	0.27572413
MCCC2	-0.514573173	0.26760624
MRPL1	-0.514573173	0.26760624
FAU	-0.514573173	0.26760624
CUL4B	-0.514573173	0.26760624
DDX28	-0.514573173	0.26760624
DYNC1LI1	-0.514573173	0.259637311
PDCD5	-0.514573173	0.259637311
MRPS9	-0.514573173	0.251811973
CARS	-0.514573173	0.244125144
PSMB6	-0.514573173	0.244125144
PES1	-0.514573173	0.244125144
DFFA	-0.514573173	0.236572006
UBE2K	-0.514573173	0.236572006
RSL1D1	-0.514573173	0.236572006
NDE1	-0.514573173	0.236572006

RTN3	-0.514573173	0.236572006
PSPH	-0.514573173	0.236572006
FIS1	-0.514573173	0.236572006
TXNDC17	-0.514573173	0.236572006
NDUFA4	-0.514573173	0.236572006
DEK	-0.514573173	0.229147988
CYB5B	-0.514573173	0.229147988
PDLIM5	-0.514573173	0.229147988
TMEM109	-0.514573173	0.229147988
RAB2A	-0.514573173	0.229147988
HMBS	-0.514573173	0.229147988
CD47	-0.514573173	0.229147988
Tmco1	-0.514573173	0.229147988
EXOSC4	-0.514573173	0.229147988
DAD1	-0.514573173	0.229147988
CPPED1	-0.514573173	0.229147988
PSMB7	-0.514573173	0.22184875
ZMPSTE24	-0.514573173	0.22184875
YJU2	-0.514573173	0.22184875
CUL2	-0.514573173	0.22184875
DCK	-0.514573173	0.214670165
NDUFV1	-0.514573173	0.214670165
RPL22L1	-0.514573173	0.214670165
EDF1	-0.514573173	0.207608311
ARFGAP1	-0.514573173	0.207608311
LARP1	-0.514573173	0.200659451
AHCYL1	-0.514573173	0.200659451
EMC2	-0.514573173	0.200659451
TM9SF3	-0.514573173	0.200659451
BORCS6	-0.514573173	0.200659451
HP1BP3	-0.514573173	0.200659451
SF3B1	-0.514573173	0.187086643
PRPF3	-0.514573173	0.187086643
GLDC	-0.514573173	0.187086643
LACTB	-0.514573173	0.173925197
CD63	-0.514573173	0.167491087
RGS10	-0.514573173	0.148741651
MRPL14	-0.514573173	0.142667504
RIOK2	-0.514573173	0.142667504
SRSF9	-0.514573173	0.142667504
ARHGEF7	-0.514573173	0.13667714
LMNB2	-0.514573173	0.13667714
COQ9	-0.514573173	0.13667714
FAM3C	-0.514573173	0.13667714
HNRNPUL2	-0.514573173	0.13076828
GTF2F2	-0.514573173	0.13076828
PPP1R8	-0.514573173	0.096910013

SEC62	-0.736965594	3.113509275
ATP1A1	-0.736965594	2.602059991
CSE1L	-0.736965594	2
NCKAP1L	-0.736965594	1.920818754
CSTF2	-0.736965594	1.48148606
DPP3	-0.736965594	1.443697499
LTA4H	-0.736965594	1.346787486
CPSF6	-0.736965594	1.283996656
BZW1	-0.736965594	1.236572006
STXBP2	-0.736965594	1.236572006
ACADM	-0.736965594	1.180456064
TXNL1	-0.736965594	1.15490196
DNM2	-0.736965594	1.13076828
TFG	-0.736965594	1.096910013
EIF6	-0.736965594	1.055517328
SRPRA	-0.736965594	1.045757491
IFI30	-0.736965594	1.008773924
CHMP4B	-0.736965594	1
PSMB9	-0.736965594	1
IPO5	-0.736965594	0.920818754
SH3KBP1	-0.736965594	0.886056648
ADK	-0.736965594	0.886056648
CLNS1A	-0.736965594	0.886056648
PPP4R3A	-0.736965594	0.795880017
Cyfip2	-0.736965594	0.795880017
FUS	-0.736965594	0.769551079
MARS	-0.736965594	0.744727495
ANP32E	-0.736965594	0.744727495
AIP	-0.736965594	0.744727495
PDAP1	-0.736965594	0.744727495
APEH	-0.736965594	0.744727495
MAT2B	-0.736965594	0.744727495
ACOT7	-0.736965594	0.721246399
API5	-0.736965594	0.698970004
PNPT1	-0.736965594	0.698970004
GFM1	-0.736965594	0.698970004
FKBP5	-0.736965594	0.677780705
TYMP	-0.736965594	0.677780705
SNX5	-0.736965594	0.677780705
TXLNA	-0.736965594	0.657577319
UGP2	-0.736965594	0.657577319
USP14	-0.736965594	0.638272164
RAD23A	-0.736965594	0.638272164
IL4I1	-0.736965594	0.638272164
RALY	-0.736965594	0.638272164
LARP7	-0.736965594	0.638272164
HCFC1	-0.736965594	0.602059991

VBP1	-0.736965594	0.602059991
PGRMC1	-0.736965594	0.602059991
PRPF4	-0.736965594	0.602059991
PPIF	-0.736965594	0.602059991
TSN	-0.736965594	0.585026652
FIP1L1	-0.736965594	0.585026652
RAP1B	-0.736965594	0.568636236
DDRGK1	-0.736965594	0.568636236
G6PD	-0.736965594	0.552841969
ADSS	-0.736965594	0.552841969
EIF2B3	-0.736965594	0.552841969
MAP2K3	-0.736965594	0.552841969
EXOSC9	-0.736965594	0.552841969
CD2AP	-0.736965594	0.537602002
DTYMK	-0.736965594	0.537602002
SDCBP	-0.736965594	0.537602002
RPS6KA3	-0.736965594	0.537602002
RPAP3	-0.736965594	0.522878745
GGCT	-0.736965594	0.522878745
IPO9	-0.736965594	0.522878745
ENO2	-0.736965594	0.494850022
DDX39B	-0.736965594	0.494850022
MRPS27	-0.736965594	0.48148606
IPO4	-0.736965594	0.48148606
BYSL	-0.736965594	0.48148606
AP1G2	-0.736965594	0.48148606
AKT2	-0.736965594	0.468521083
CRKL	-0.736965594	0.468521083
SRP19	-0.736965594	0.468521083
Aars	-0.736965594	0.468521083
NOB1	-0.736965594	0.443697499
HAT1	-0.736965594	0.443697499
MARS2	-0.736965594	0.443697499
IK	-0.736965594	0.431798276
VARS	-0.736965594	0.420216403
TOE1	-0.736965594	0.420216403
TOR1AIP1	-0.736965594	0.420216403
DCTPP1	-0.736965594	0.408935393
NNT	-0.736965594	0.397940009
SYAP1	-0.736965594	0.397940009
NCAPG	-0.736965594	0.387216143
PACSIN2	-0.736965594	0.387216143
HLA-DRB3	-0.736965594	0.387216143
SEC24A	-0.736965594	0.387216143
NSDHL	-0.736965594	0.37675071
URI1	-0.736965594	0.37675071
DBI	-0.736965594	0.37675071

MRPS23	-0.736965594	0.37675071
MAX	-0.736965594	0.37675071
KPNA4	-0.736965594	0.37675071
SPATA5	-0.736965594	0.37675071
OSBP	-0.736965594	0.366531544
ITPK1	-0.736965594	0.366531544
TNFAIP8	-0.736965594	0.366531544
METTL1	-0.736965594	0.356547324
HRNR	-0.736965594	0.356547324
ATP6V1E1	-0.736965594	0.356547324
SLC9A3R1	-0.736965594	0.346787486
UBA6	-0.736965594	0.346787486
MTREX	-0.736965594	0.346787486
EYA3	-0.736965594	0.346787486
EIF2D	-0.736965594	0.346787486
AGFG1	-0.736965594	0.337242168
SMS	-0.736965594	0.337242168
GIT2	-0.736965594	0.337242168
SCAMP2	-0.736965594	0.337242168
PEX19	-0.736965594	0.337242168
PBDC1	-0.736965594	0.318758763
PRKD2	-0.736965594	0.30980392
IFI35	-0.736965594	0.301029996
LSR	-0.736965594	0.292429824
TMEM70	-0.736965594	0.292429824
SUPV3L1	-0.736965594	0.292429824
NEK9	-0.736965594	0.292429824
NOP9	-0.736965594	0.292429824
BAG5	-0.736965594	0.27572413
CTH	-0.736965594	0.27572413
STK10	-0.736965594	0.259637311
ARHGAP45	-0.736965594	0.259637311
STOM	-0.736965594	0.259637311
AGPAT5	-0.736965594	0.251811973
SARS2	-0.736965594	0.251811973
DUS3L	-0.736965594	0.251811973
LYPLA2	-0.736965594	0.236572006
CD37	-0.736965594	0.236572006
SCAMP1	-0.736965594	0.236572006
ABI1	-0.736965594	0.236572006
TMED2	-0.736965594	0.236572006
PGP	-0.736965594	0.236572006
STX18	-0.736965594	0.229147988
RBM3	-0.736965594	0.214670165
AK3	-0.736965594	0.214670165
LSS	-0.736965594	0.214670165
PAIP1	-0.736965594	0.214670165

PLEKHA2	-0.736965594	0.214670165
PPP2R1B	-0.736965594	0.207608311
PRKRA	-0.736965594	0.193820026
IKZF2	-0.736965594	0.193820026
LTF	-0.736965594	0.187086643
DNPEP	-0.736965594	0.187086643
CDC23	-0.736965594	0.187086643
HM13	-0.736965594	0.187086643
NOMO3	-0.736965594	0.15490196
MAPKAPK2	-0.736965594	0.148741651
ETV6	-0.736965594	0.148741651
MYBBP1A	-0.736965594	0.13076828
DARS2	-0.736965594	0.13076828
WDHD1	-0.736965594	0.119186408
BCCIP	-1	1.958607315
NDUFS2	-1	1.795880017
UBE3A	-1	1.657577319
ARHGAP4	-1	1.619788758
WBP11	-1	1.537602002
OXSR1	-1	1.522878745
ANXA11	-1	1.522878745
CPT2	-1	1.508638306
ERO1A	-1	1.443697499
FKBP8	-1	1.431798276
CAST	-1	1.37675071
PRKCD	-1	1.366531544
FEN1	-1	1.337242168
PSMA2	-1	1.22184875
THOP1	-1	1.167491087
SEC23B	-1	1.080921908
PTCD3	-1	1.075720714
CALU	-1	1.070581074
ADD1	-1	1.050609993
SAMHD1	-1	1.017728767
ALDH16A1	-1	1.008773924
TBC1D15	-1	1.004364805
ENSA	-1	1.004364805
RCSD1	-1	1
CLINT1	-1	1
HARS	-1	0.958607315
LPXN	-1	0.958607315
VPS4B	-1	0.958607315
NMT1	-1	0.920818754
CIAPIN1	-1	0.920818754
CRYZ	-1	0.920818754
XPO5	-1	0.886056648
SRSF7	-1	0.886056648

ATP5PB	-1	0.853871964
ERAP1	-1	0.853871964
RABGAP1L	-1	0.853871964
VAV1	-1	0.853871964
POLDIP2	-1	0.853871964
CTSH	-1	0.853871964
MTA1	-1	0.853871964
HSPB1	-1	0.823908741
ALDH1B1	-1	0.769551079
SRPRB	-1	0.769551079
AARSD1	-1	0.769551079
NUCB1	-1	0.744727495
ATP2A2	-1	0.744727495
BCAT1	-1	0.744727495
Pura	-1	0.744727495
APPL1	-1	0.744727495
EBNA1BP2	-1	0.744727495
THUMPD3	-1	0.744727495
C11orf98	-1	0.744727495
PSMD14	-1	0.721246399
EIF1AX	-1	0.721246399
TIPIN	-1	0.721246399
DCUN1D1	-1	0.721246399
IARS2	-1	0.721246399
RMDN1	-1	0.721246399
FARS2	-1	0.721246399
CHORDC1	-1	0.698970004
SAMSN1	-1	0.698970004
SSU72	-1	0.698970004
ECSIT	-1	0.698970004
ATG3	-1	0.698970004
STT3A	-1	0.677780705
WDR61	-1	0.677780705
SNX8	-1	0.677780705
PLPBP	-1	0.677780705
COA7	-1	0.677780705
SEC61B	-1	0.677780705
GSR	-1	0.657577319
SRSF6	-1	0.657577319
ALDH9A1	-1	0.657577319
FTO	-1	0.657577319
APRT	-1	0.638272164
DYNC1I2	-1	0.638272164
TNPO3	-1	0.638272164
OSTF1	-1	0.638272164
UCK2	-1	0.638272164
SNX27	-1	0.638272164

ASCC2	-1	0.638272164
IMPDH1	-1	0.638272164
ZPR1	-1	0.619788758
Csnk2a1	-1	0.619788758
CUSTOS	-1	0.619788758
RBM12	-1	0.602059991
SART3	-1	0.602059991
COPS3	-1	0.602059991
WIPF1	-1	0.602059991
COX5B	-1	0.602059991
CD70	-1	0.585026652
NSF	-1	0.585026652
MRPL37	-1	0.568636236
DRAP1	-1	0.568636236
HACD3	-1	0.568636236
QRICH1	-1	0.568636236
NARS2	-1	0.568636236
UCHL3	-1	0.552841969
MPP6	-1	0.552841969
HMOX2	-1	0.552841969
DDX20	-1	0.552841969
DHPS	-1	0.552841969
PEA15	-1	0.552841969
EPB41	-1	0.522878745
PDCL3	-1	0.522878745
TMX2	-1	0.522878745
ELOC	-1	0.522878745
SP100	-1	0.508638306
WDR82	-1	0.494850022
GPKOW	-1	0.443697499
WASH2P	-1	0.431798276
CAMK1D	-1	0.408935393
SNUPN	-1	0.397940009
IRGQ	-1	0.387216143
LAMTOR1	-1	0.387216143
YAF2	-1	0.387216143
SEC11C	-1	0.387216143
GCDH	-1	0.37675071
GPATCH4	-1	0.37675071
KRT6A	-1	0.366531544
CCDC47	-1	0.356547324
CNOT10	-1	0.318758763
STK38L	-1	0.318758763
RBM42	-1	0.301029996
GMPPB	-1	0.301029996
SEC63	-1	0.301029996
HEBP2	-1	0.301029996

CEP41	-1	0.27572413
SLAIN2	-1	0.251811973
TMED8	-1	0.251811973
CLCC1	-1	0.236572006
MRPL15	-1	0.236572006
PITPNA	-1	0.236572006
RNPEP	-1	0.200659451
DPH2	-1	0.187086643
Rad21	-1	0.187086643
HSF1	-1	0.180456064
OPTN	-1	0.180456064
PPCS	-1	0.173925197
EEFSEC	-1	0.173925197
NUDT1	-1	0.173925197
DHRS4	-1	0.173925197
Emc7	-1	0.173925197
CHAF1B	-1	0.173925197
CCDC25	-1	0.173925197
SGPL1	-1	0.173925197
MRPS25	-1	0.161150909
NOP58	-1.321928095	2.823908741
CLPTM1	-1.321928095	2.823908741
NMI	-1.321928095	2.537602002
SOD1	-1.321928095	2.214670165
APMAP	-1.321928095	1.698970004
TKFC	-1.321928095	1.619788758
ZRANB2	-1.321928095	1.366531544
IFI44	-1.321928095	1.301029996
NAA15	-1.321928095	1.26760624
TTC1	-1.321928095	1.229147988
CDV3	-1.321928095	1.22184875
NT5DC1	-1.321928095	1.187086643
NELFA	-1.321928095	1.161150909
PTPN11	-1.321928095	1.107905397
PHF6	-1.321928095	1.107905397
SRSF11	-1.321928095	1.107905397
NUDCD1	-1.321928095	1.107905397
COASY	-1.321928095	1.096910013
SLC25A13	-1.321928095	1.080921908
SLC4A1AP	-1.321928095	1.070581074
SPC24	-1.321928095	1.070581074
NELFE	-1.321928095	1.055517328
UBE2J1	-1.321928095	1.055517328
MRPS18B	-1.321928095	1.036212173
POR	-1.321928095	1.031517051
DPH5	-1.321928095	1.013228266
RBM39	-1.321928095	0.958607315

GSDME	-1.321928095	0.958607315
DDX42	-1.321928095	0.920818754
SNRNP70	-1.321928095	0.920818754
STK24	-1.321928095	0.920818754
NFKB2	-1.321928095	0.886056648
UFD1	-1.321928095	0.886056648
DRG2	-1.321928095	0.853871964
FAM192A	-1.321928095	0.853871964
GNL1	-1.321928095	0.853871964
CELF2	-1.321928095	0.853871964
LARS	-1.321928095	0.823908741
WDR12	-1.321928095	0.795880017
FABP5	-1.321928095	0.744727495
WDR18	-1.321928095	0.744727495
TTC4	-1.321928095	0.721246399
GRK2	-1.321928095	0.721246399
Mbnl1	-1.321928095	0.698970004
CAT	-1.321928095	0.677780705
TFEB	-1.321928095	0.677780705
Slc38a1	-1.321928095	0.677780705
SPCS3	-1.321928095	0.638272164
MRPS31	-1.321928095	0.619788758
C12orf45	-1.321928095	0.619788758
FLAD1	-1.321928095	0.619788758
RNF113A	-1.321928095	0.602059991
PPP1R2	-1.321928095	0.602059991
CD40	-1.321928095	0.602059991
UBQLN4	-1.321928095	0.585026652
MED4	-1.321928095	0.568636236
HGS	-1.321928095	0.552841969
CLIP2	-1.321928095	0.552841969
SNRNP40	-1.321928095	0.494850022
REL	-1.321928095	0.48148606
LMAN2	-1.321928095	0.48148606
NFKBIE	-1.321928095	0.48148606
CD22	-1.321928095	0.468521083
RTF1	-1.321928095	0.468521083
SQSTM1	-1.321928095	0.431798276
MAGED2	-1.321928095	0.431798276
PAFAH1B3	-1.321928095	0.431798276
CLPB	-1.321928095	0.397940009
NAPG	-1.321928095	0.37675071
MRPS5	-1.321928095	0.356547324
MBD2	-1.321928095	0.346787486
DEF6	-1.321928095	0.292429824
MAGOH	-1.321928095	0.283996656
NLRP2	-1.321928095	0.200659451

RBM25	-1.736965594	1.920818754
PAFAH1B1	-1.736965594	1.920818754
GTF3C5	-1.736965594	1.920818754
RBM28	-1.736965594	1.920818754
DYNC1LI2	-1.736965594	1.823908741
BPNT1	-1.736965594	1.744727495
ACSL4	-1.736965594	1.677780705
FAF2	-1.736965594	1.677780705
VPS26A	-1.736965594	1.677780705
MRE11	-1.736965594	1.552841969
NPLOC4	-1.736965594	1.508638306
HPDL	-1.736965594	1.508638306
JAK3	-1.736965594	1.431798276
GYS1	-1.736965594	1.346787486
SART1	-1.736965594	1.301029996
Srsf5	-1.736965594	1.26760624
HMGB3	-1.736965594	1.259637311
BAG1	-1.736965594	1.050609993
MFAP1	-1.736965594	1.050609993
JUNB	-1.736965594	1.050609993
PITPNB	-1.736965594	0.958607315
DAZAP1	-1.736965594	0.958607315
OSBPL9	-1.736965594	0.920818754
PREP	-1.736965594	0.920818754
TMED10	-1.736965594	0.853871964
ILK	-1.736965594	0.823908741
RNMT	-1.736965594	0.744727495
PTPN7	-1.736965594	0.744727495
TAOK3	-1.736965594	0.677780705
THRAP3	-1.736965594	0.677780705
RAB1B	-1.736965594	0.552841969
TRMT6	-1.736965594	0.48148606
UGDH	-1.736965594	0.48148606
GGA2	-1.736965594	0.48148606
NCAPD2	-1.736965594	0.48148606
FAM114A2	-1.736965594	0.468521083
SEC11A	-1.736965594	0.468521083
YKT6	-1.736965594	0.468521083
MRPS7	-1.736965594	0.468521083
NECAP2	-1.736965594	0.468521083
RNF20	-1.736965594	0.468521083
NEXN	-1.736965594	0.468521083
SAMD1	-1.736965594	0.468521083
KRI1	-1.736965594	0.443697499
IFNGR1	-1.736965594	0.443697499
PPP1R14B	-1.736965594	0.431798276
AMPD2	-1.736965594	0.431798276

TMEM263	-1.736965594	0.431798276
ERGIC3	-1.736965594	0.420216403
UBE2R2	-1.736965594	0.420216403
HEXIM1	-1.736965594	0.420216403
CD2BP2	-1.736965594	0.420216403
NRBP1	-1.736965594	0.420216403
CD180	-1.736965594	0.420216403
PMM2	-1.736965594	0.37675071
POLR2C	-1.736965594	0.251811973
CD300A	-2.321928095	2.124938737
TROVE2	-2.321928095	2.065501549
NOLC1	-2.321928095	2.022276395
TOMM22	-2.321928095	1.638272164
MTMR14	-2.321928095	1.494850022
TBCC	-2.321928095	1.494850022
PTBP3	-2.321928095	1.408935393
MOB3A	-2.321928095	1.327902142
PARVB	-2.321928095	1.292429824
GORASP2	-2.321928095	1.244125144
DPF2	-2.321928095	1.161150909
MAPK14	-2.321928095	1.148741651
PEX14	-2.321928095	1.124938737
L2HGDH	-2.321928095	1.102372909
GSTM1	-2.321928095	1.102372909
GTF2A1	-2.321928095	1.102372909
SMARCD2	-2.321928095	1.017728767
FNTA	-2.321928095	1
RNASET2	-2.321928095	1
CIP2A	-2.321928095	1
MANF	-2.321928095	1
IRAK4	-2.321928095	1
TRIP13	-2.321928095	0.823908741
RDH11	-2.321928095	0.823908741
SETD3	-2.321928095	0.823908741
HOOK3	-2.321928095	0.657577319
ST6GAL1	-2.321928095	0.657577319
LPGAT1	-2.321928095	0.638272164
KCNAB2	-2.321928095	0.638272164
OTULIN	-2.321928095	0.619788758
IRF2BPL	-2.321928095	0.48148606
ZFAND6	-2.321928095	0.48148606
ALB	-3.321928095	3.37675071
DTX3L	-3.321928095	1.30980392
SPAG7	-3.321928095	1.301029996
BIN2	-3.321928095	1.180456064
RRP1	-3.321928095	1.180456064
TCOF1	-3.836501268	0.823908741

REFERENCES

1 Chen, H. C., Byrd, J. C. & Muthusamy, N. Differential role for cyclic AMP response element binding protein-1 in multiple stages of B cell development, differentiation, and survival. *Journal of Immunology* **176**, 2208-2218 (2006). https://doi.org:10.4049/jimmunol.176.4.2208

2 Bichi, R. *et al.* Human chronic lymphocytic leukemia modeled in mouse by targeted TCL1 expression. *Proc Natl Acad Sci U S A* **99**, 6955-6960 (2002). https://doi.org:10.1073/pnas.102181599

3 Johnson, A. J. *et al.* Characterization of the TCL-1 transgenic mouse as a preclinical drug development tool for human chronic lymphocytic leukemia. *Blood* **108**, 1334-1338 (2006). https://doi.org:10.1182/blood-2005-12-011213

4 Chang, J. *et al.* Siglec-6 on Chronic Lymphocytic Leukemia Cells Is a Target for Post-Allogeneic Hematopoietic Stem Cell Transplantation Antibodies. *Cancer Immunology Research* **6**, 1008-1013 (2018). https://doi.org:10.1158/2326-6066.cir-18-0102

5 Cyr, M. G. *et al.* Patient-derived Siglec-6-targeting antibodies engineered for T-cell recruitment have potential therapeutic utility in chronic lymphocytic leukemia. *J Immunother Cancer* **10** (2022). https://doi.org:10.1136/jitc-2022-004850

6 Bagriaçik, E. U. & Miller, K. S. Cell surface sialic acid and the regulation of immune cell interactions: the neuraminidase effect reconsidered. *Glycobiology* **9**, 267-275 (1999). https://doi.org:10.1093/glycob/9.3.267

7 Ghendler, Y., Smolyar, A., Chang, H. C. & Reinherz, E. L. One of the CD3epsilon subunits within a T cell receptor complex lies in close proximity to the Cbeta FG loop. *J Exp Med* **187**, 1529-1536 (1998). https://doi.org:10.1084/jem.187.9.1529

8 Wang, N. *et al.* Expression of a CD3 epsilon transgene in CD3 epsilon(null) mice does not restore CD3 gamma and delta expression but efficiently rescues T cell development from a subpopulation of prothymocytes. *Int Immunol* **10**, 1777-1788 (1998). https://doi.org:10.1093/intimm/10.12.1777

Unprocessed western blots

Supplementary Fig S5a

1) **Cytoplasmic fraction CLL vs normal donor**

1: CLL untreated

2: CLL sTn

3: Normal donor untreated

4: Normal donor sTn

Western blot images were acquired using BioRad Chemidoc imaging system.

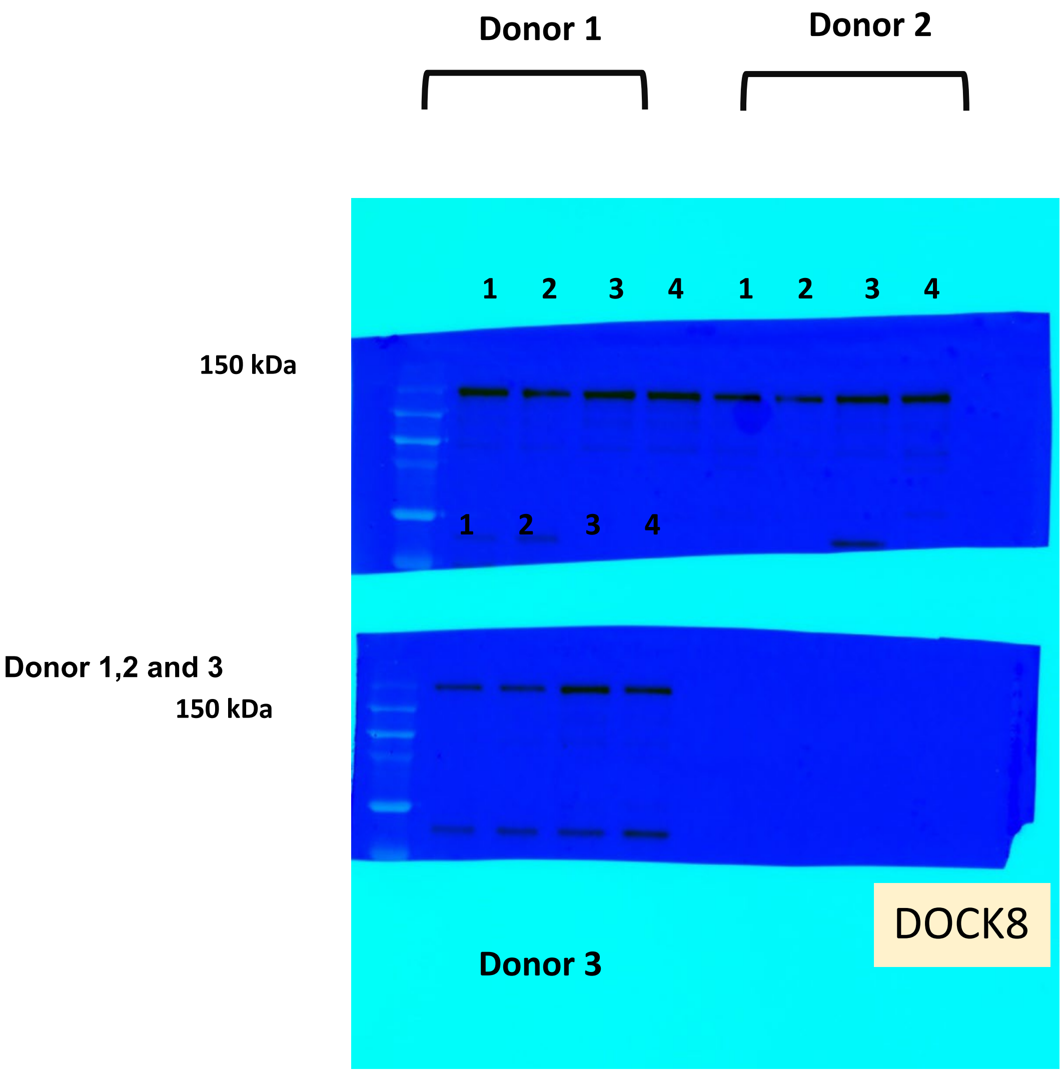

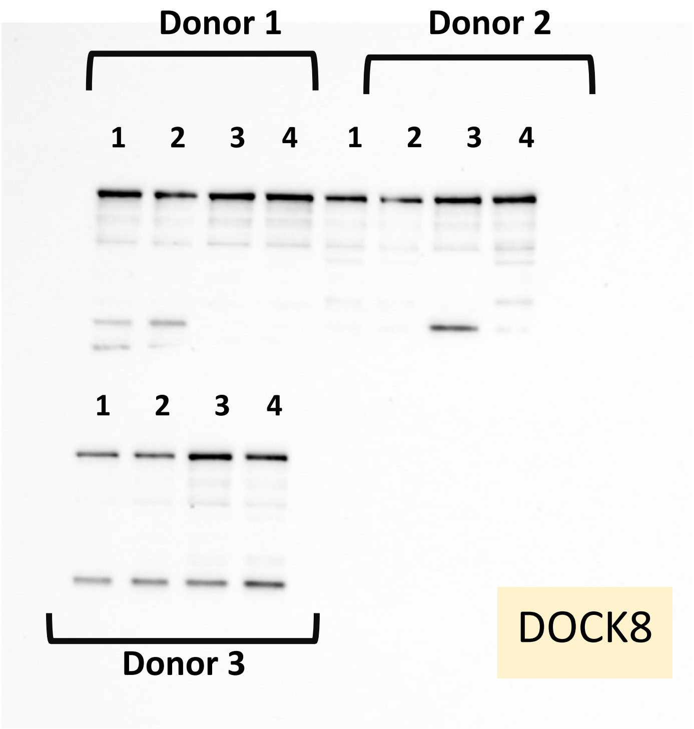
Donor 1
Donor 2
1 2 3 4 1 2 3 4
1 2 3 4
Donor 3
DOCK8

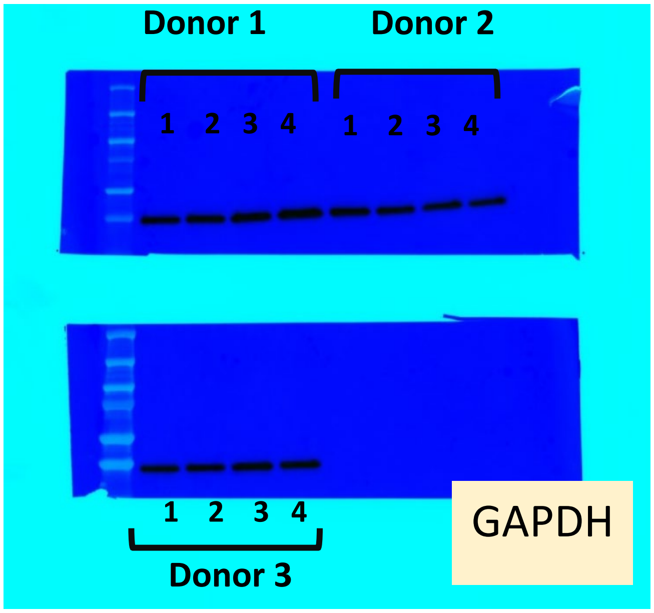
Donor 1
Donor 2
1 2 3 4 1 2 3 4
1 2 3 4
Donor 3
GAPDH

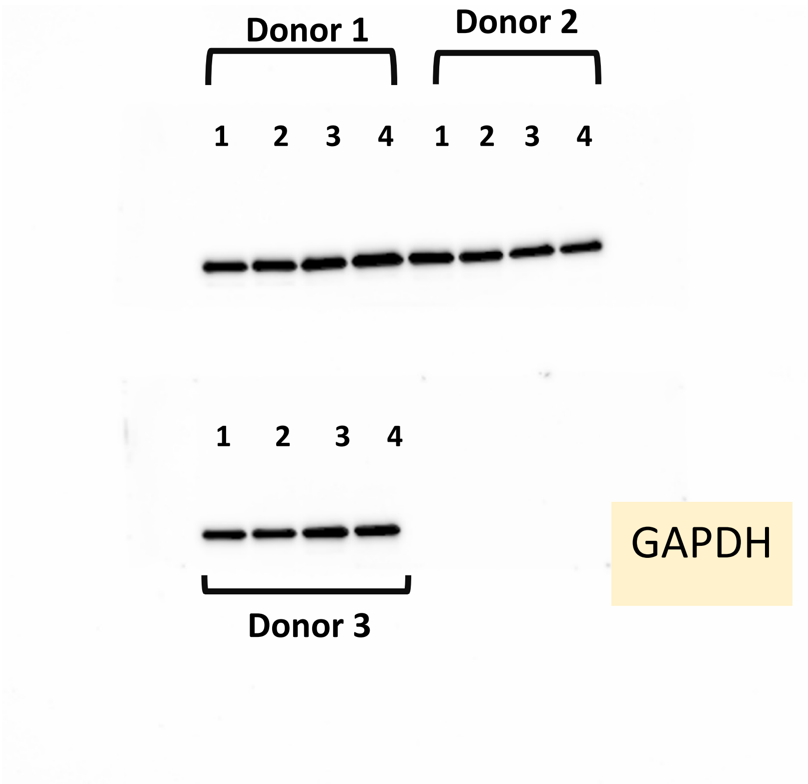
Donor 1
Donor 2
1 2 3 4 1 2 3 4
1 2 3 4
GAPDH
Donor 3

Donor 4 and 5

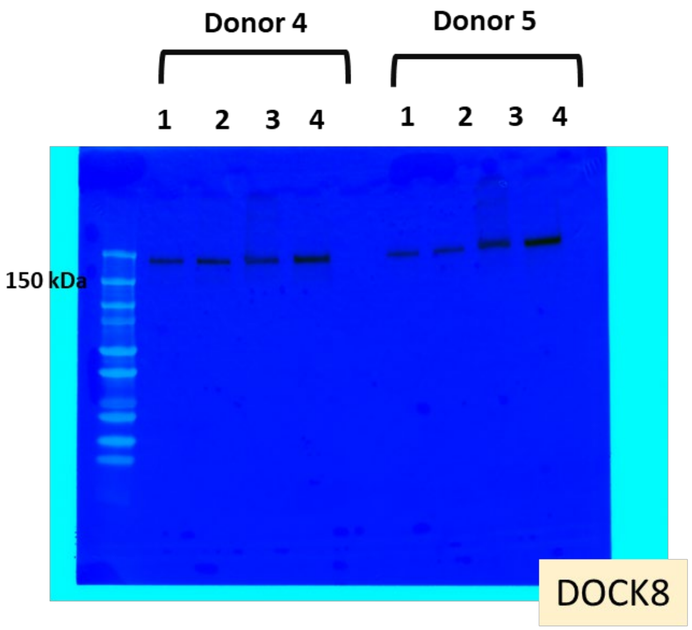
Donor 4
Donor 5
1 2 3 4
1 2 3 4
150 kDa
DOCK8

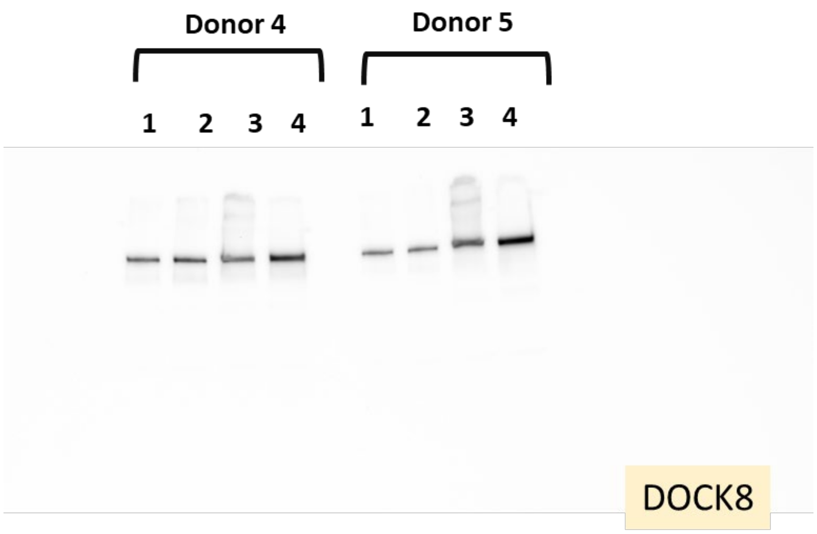
Donor 4
Donor 5
1 2 3 4
1 2 3 4
DOCK8

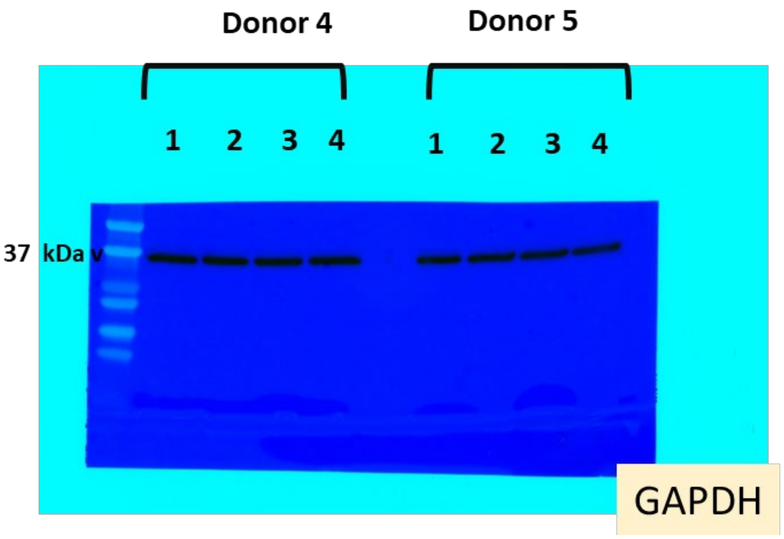
Donor 4
Donor 5
1 2 3 4
1 2 3 4
37 kDa
GAPDH

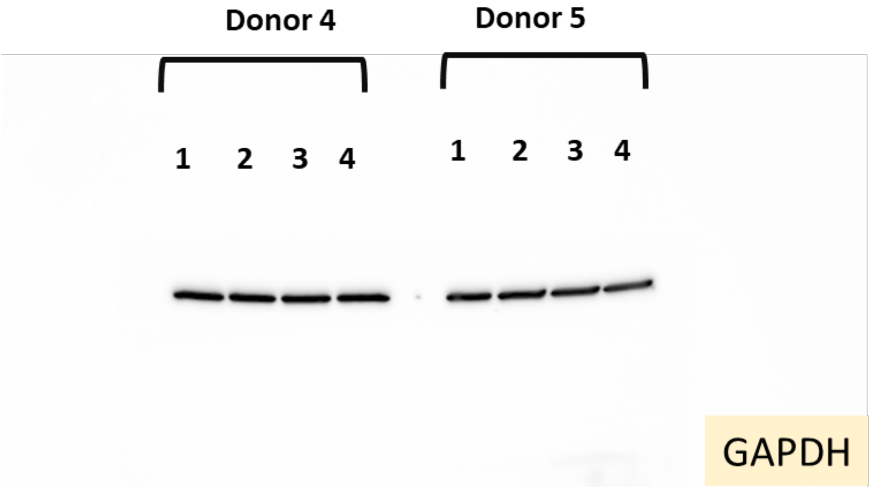

For Siglec-6 expression, only one donor was chosen because all these samples were analysed for Siglec-6 expression in Fig 1a.

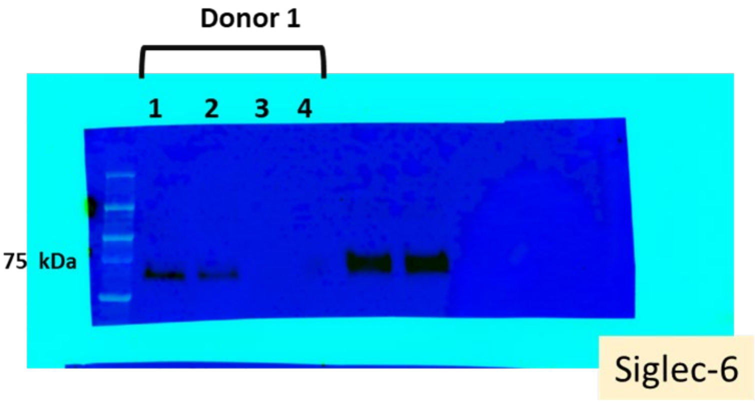

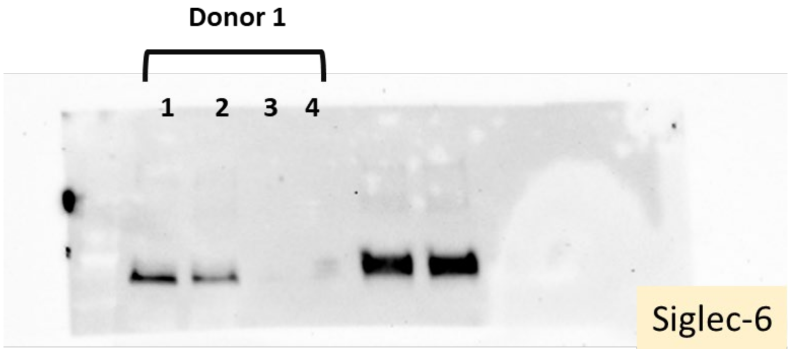

2) **Cytoplasmic fraction MEC1-002 WT, Siglec-6 KO and DOCK8 KO**

1: WT untreated

2: WT sTn

3: Siglec-6 KO untreated

4: Siglec-6 KO sTn

3: DOCK8 KO untreated

4: DOCK8 KO sTn

Western blot images were acquired using X ray films

Replicate 1

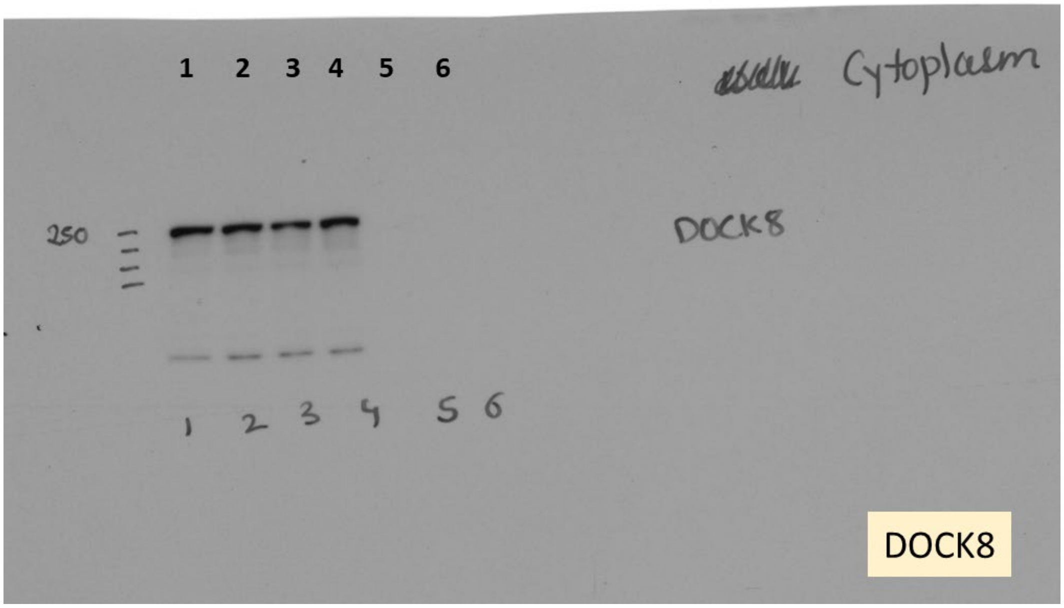

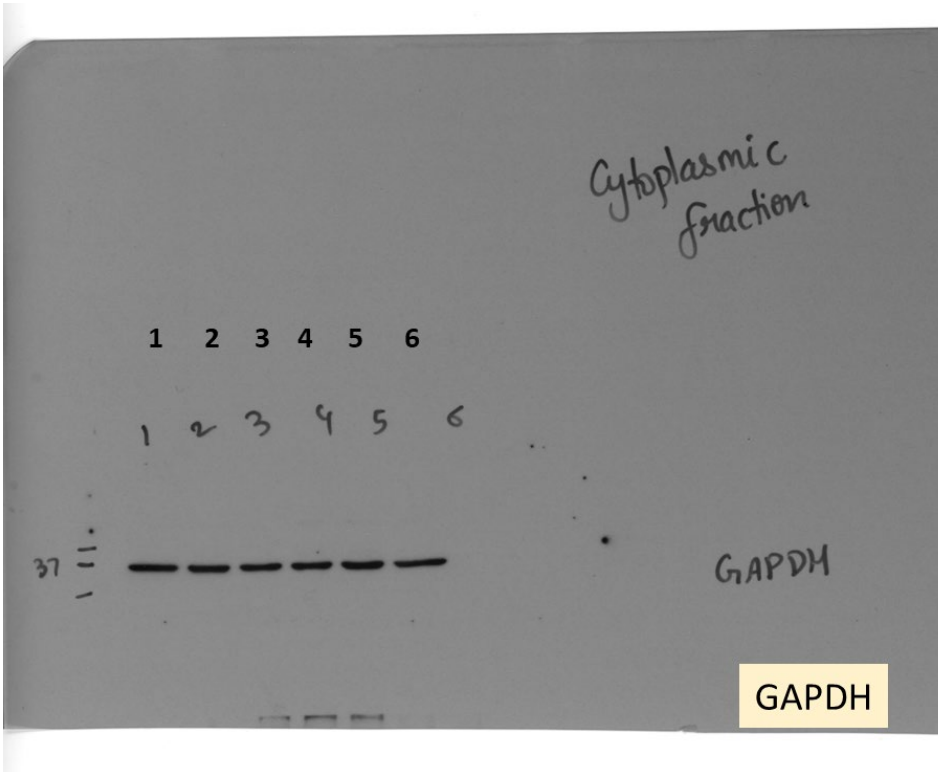
Cytoplasmic fraction
1 2 3 4 5 6
1 2 3 4 5 6
37
GAPDH
GAPDH

Replicate 2

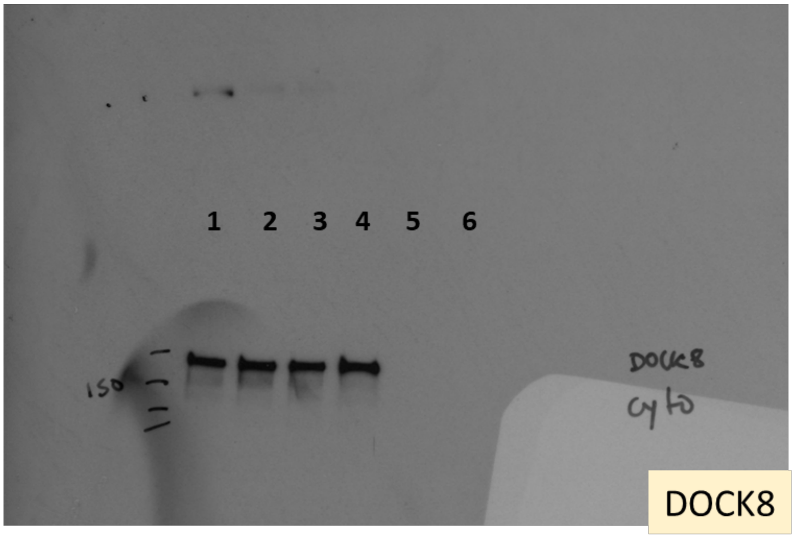
1 2 3 4 5 6
150
DOCK8
cyto
DOCK8

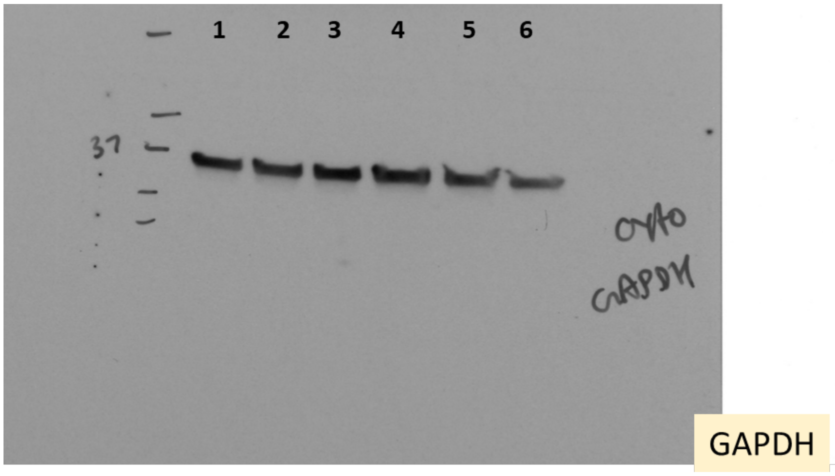

Replicate 3

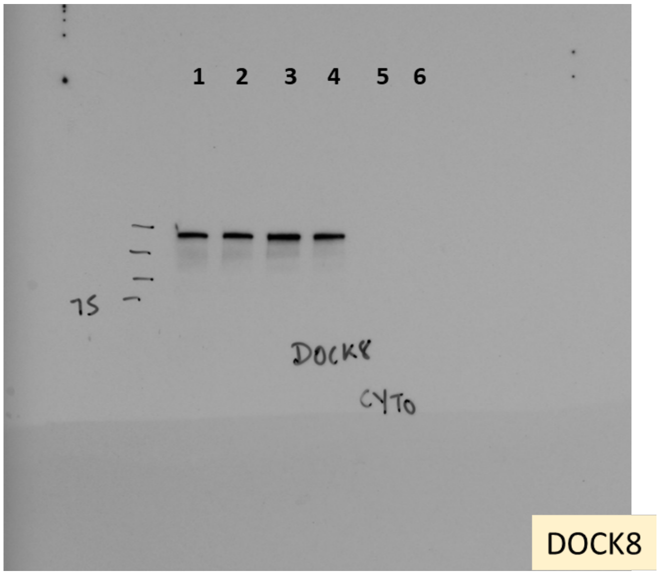

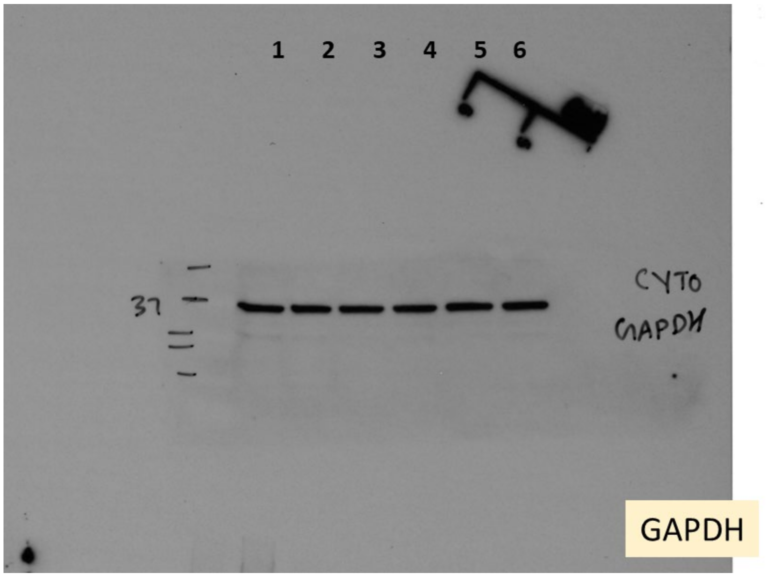

Supplementary Fig S5b

1) **Nuclear fraction CLL vs normal donor**

1: CLL untreated

2: CLL sTn

3: Normal donor untreated

4: Normal donor sTn

Western blot images were acquired using BioRad Chemidoc imaging system.

Donor 1

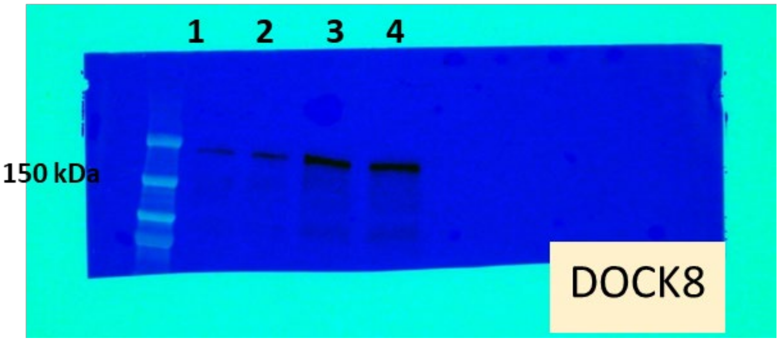

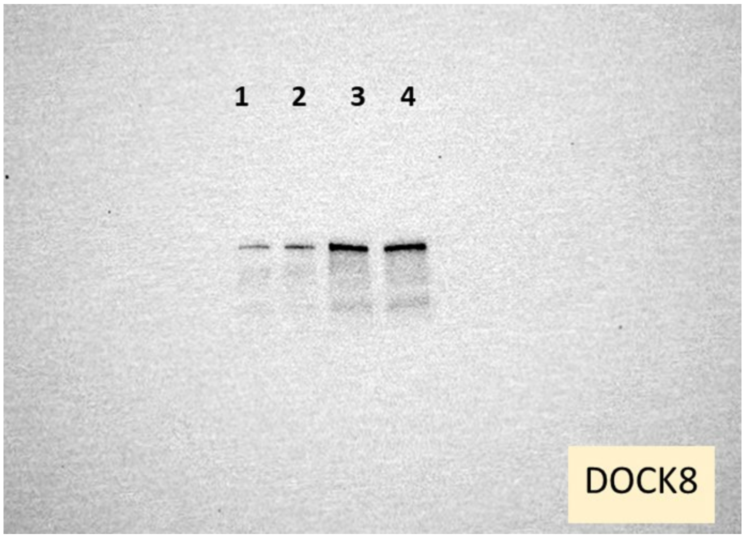

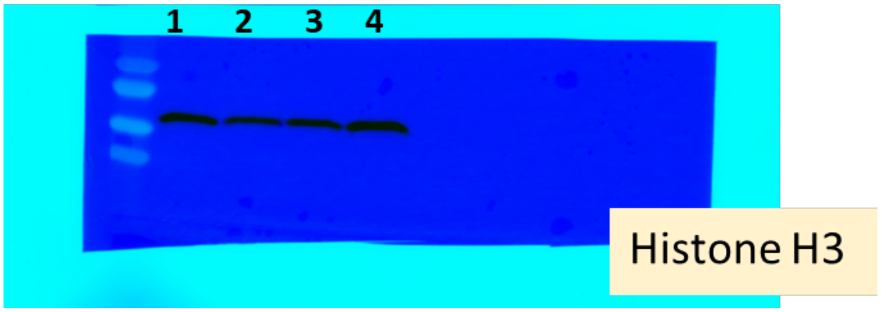

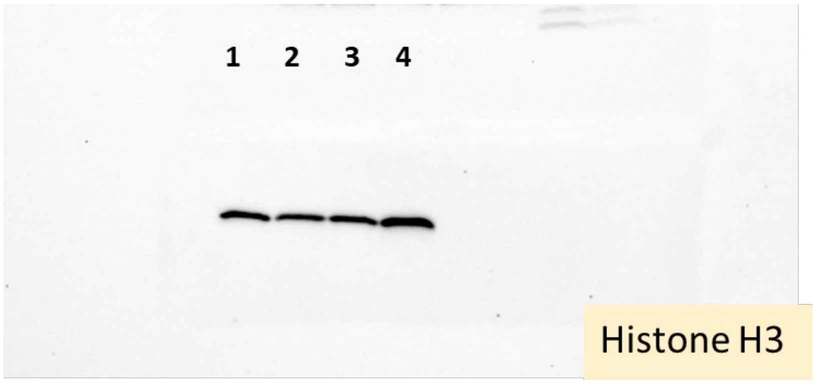

Donor 2

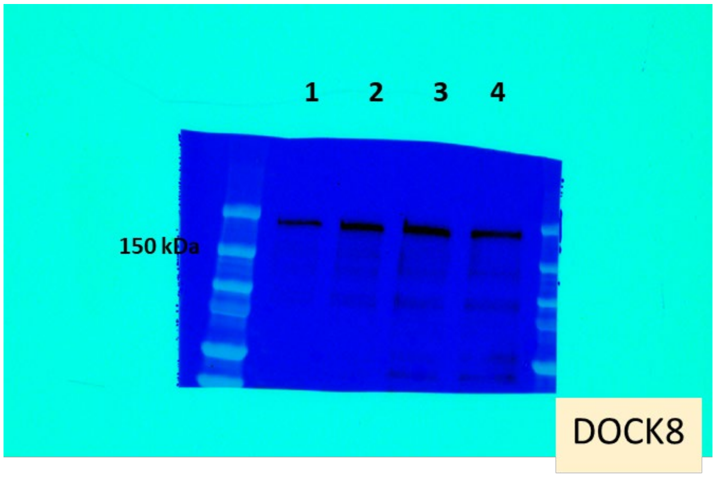

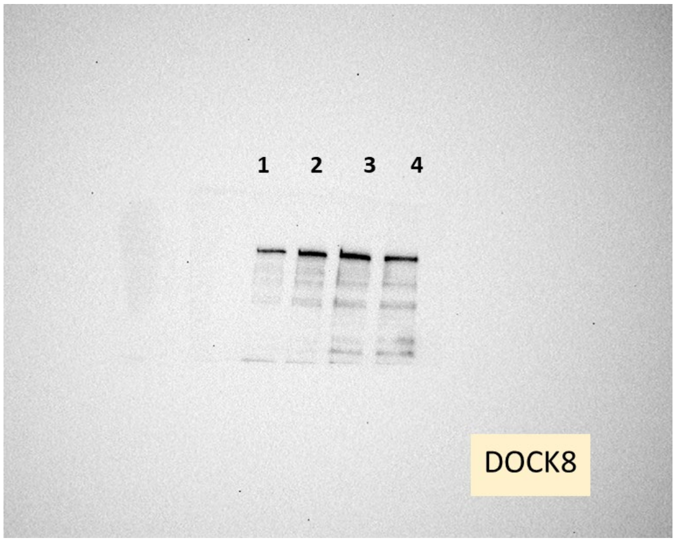

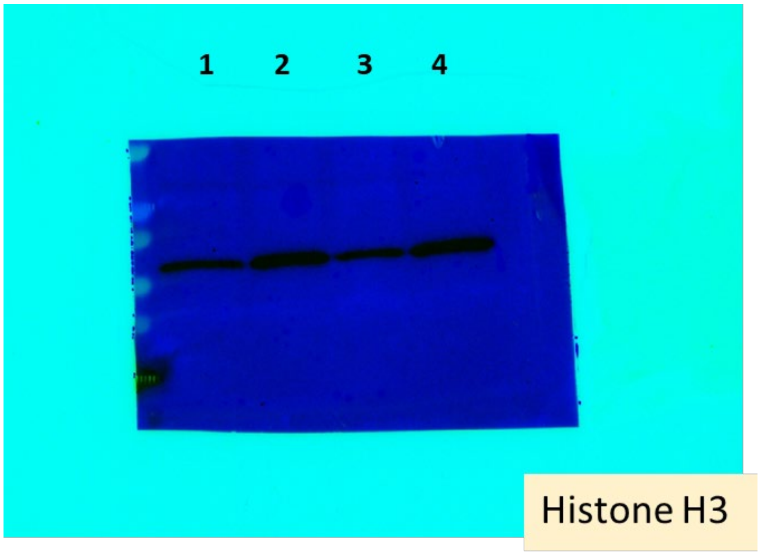
1 2 3 4
Histone H3

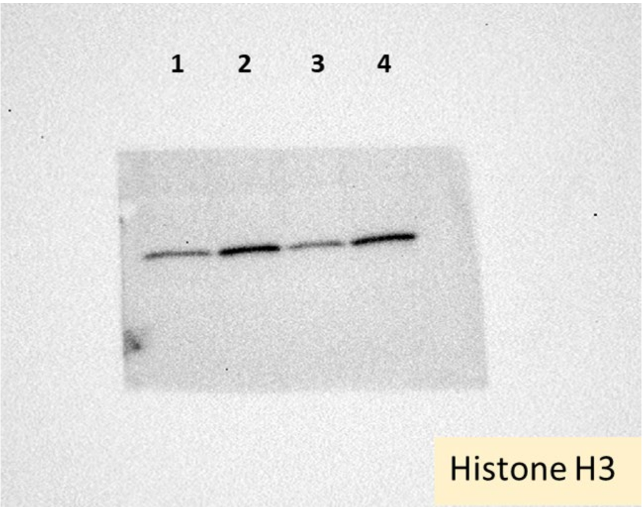
1 2 3 4
Histone H3

Donor 3

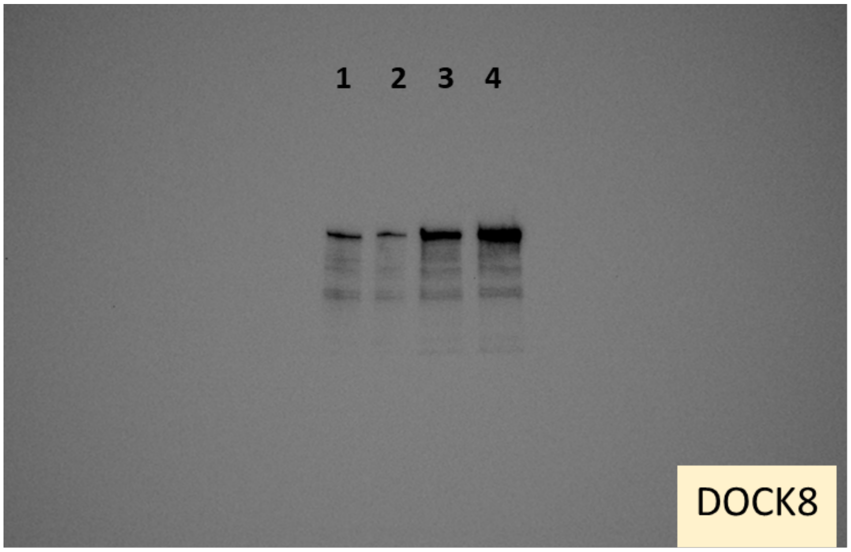

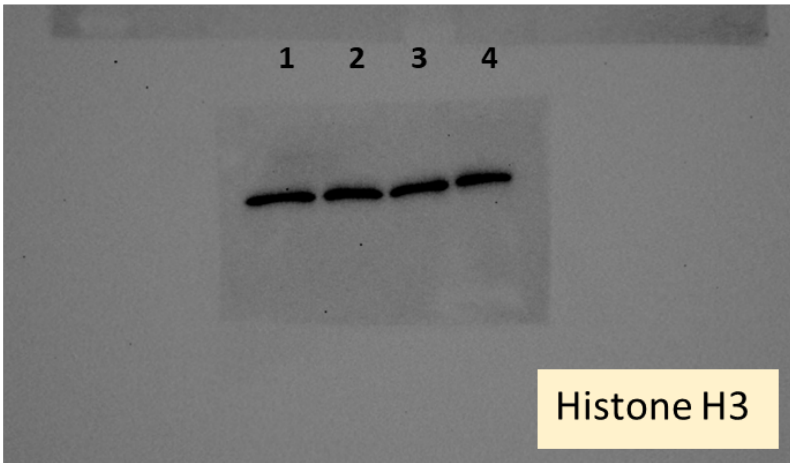

Donor 4 and 5

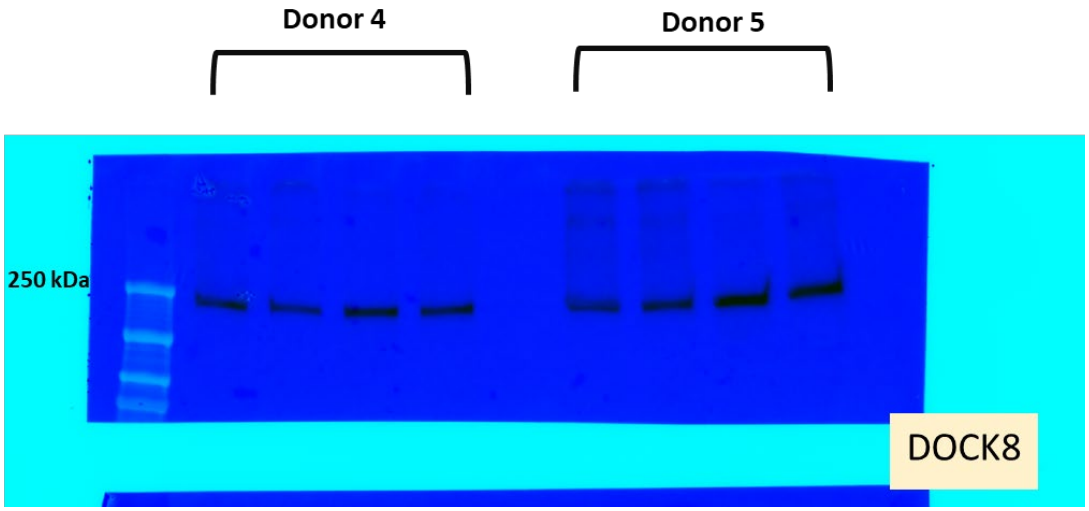

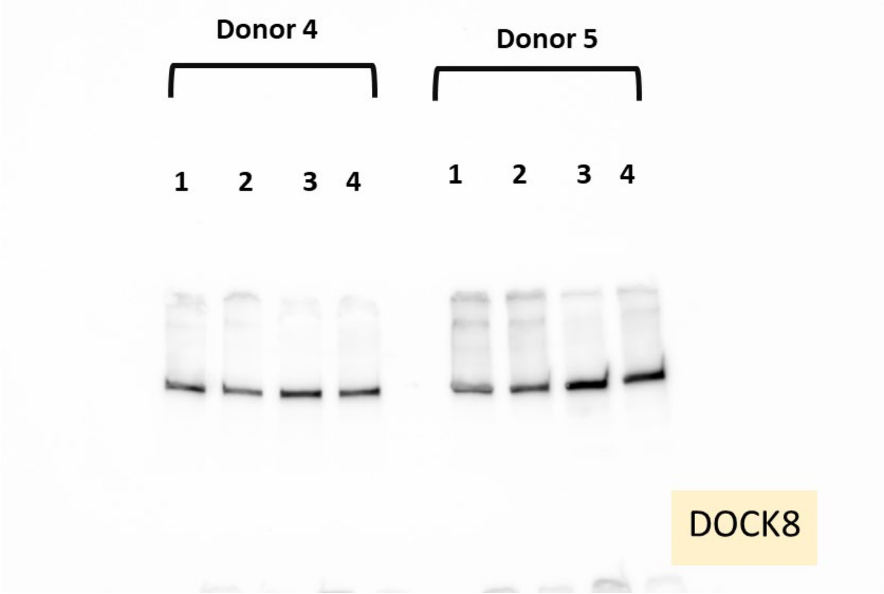

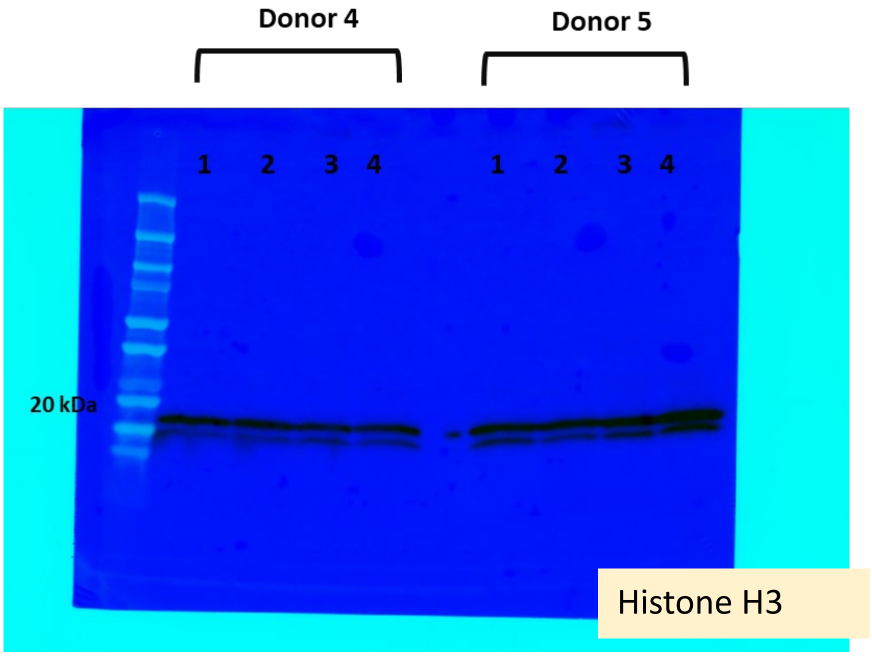
Donor 4
Donor 5
1 2 3 4
1 2 3 4
20 kDa
Histone H3

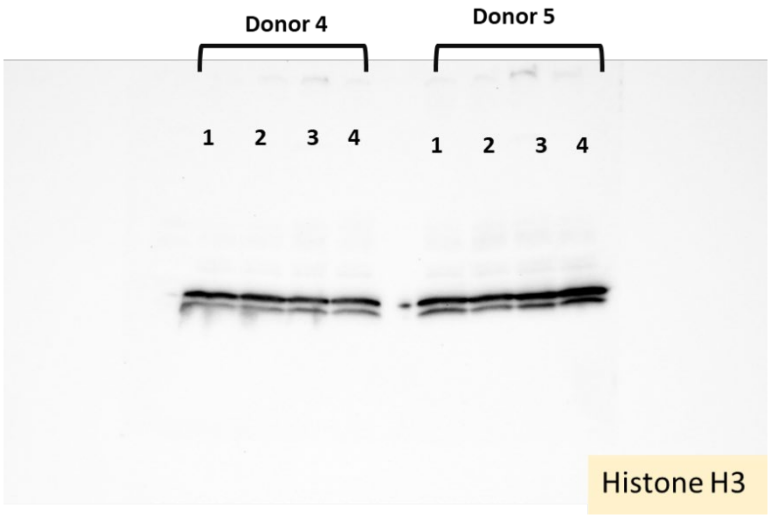
Donor 4
Donor 5
1 2 3 4
1 2 3 4
Histone H3

For Siglec-6 expression, only one donor was chosen because all these samples were analysed for Siglec-6 expression in Fig 1a.

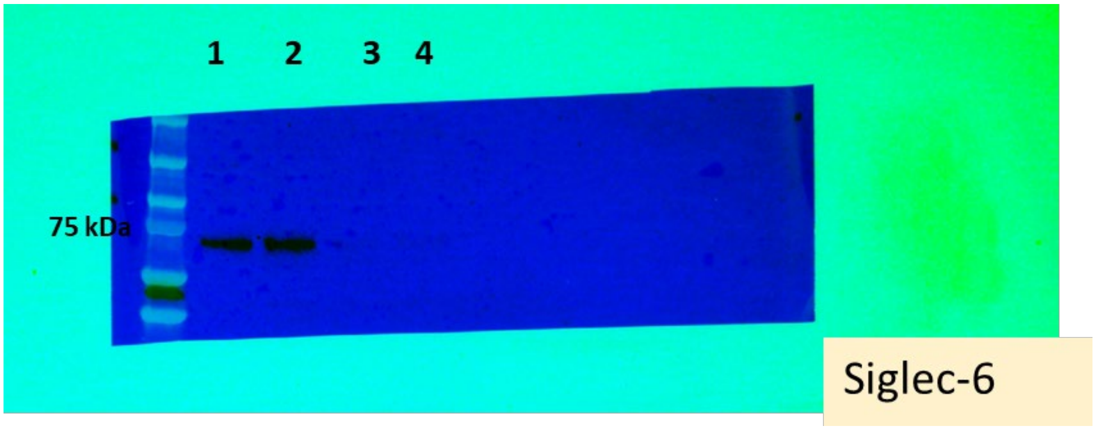

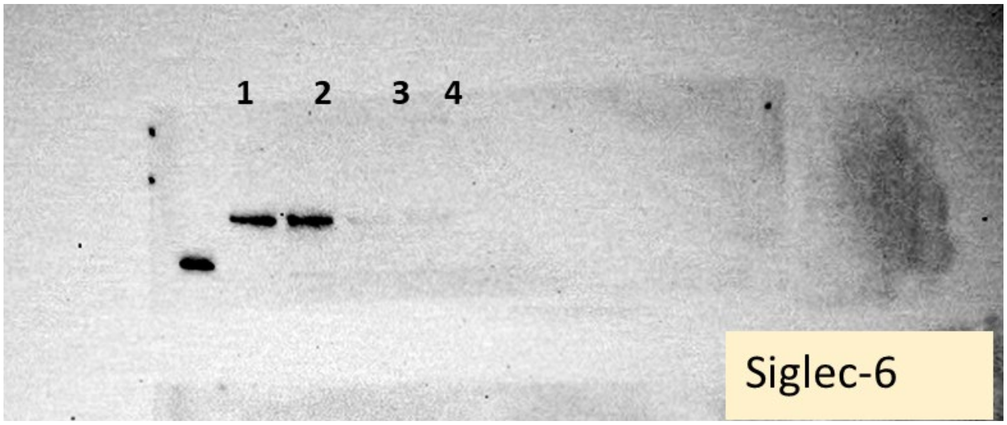

2) Nuclear fraction MEC1-002 WT, Siglec-6 KO and DOCK8 KO

1: WT untreated

2: WT sTn

3: Siglec-6 KO untreated

4: Siglec-6 KO sTn

3: DOCK8 KO untreated

4: DOCK8 KO sTn

Western blot images were acquired using BioRad Chemidoc imaging system

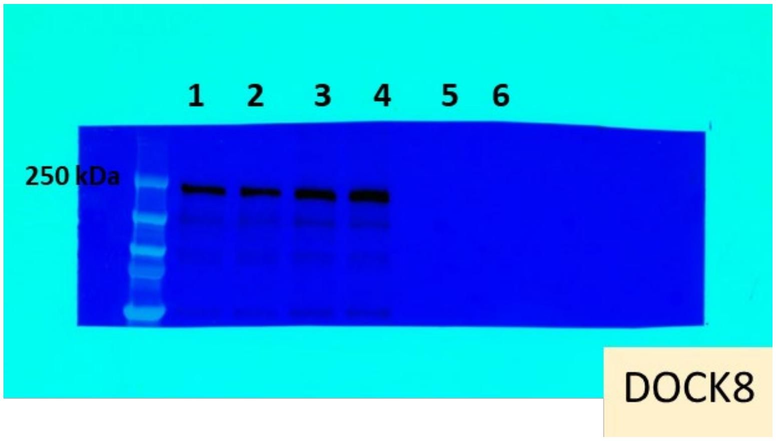

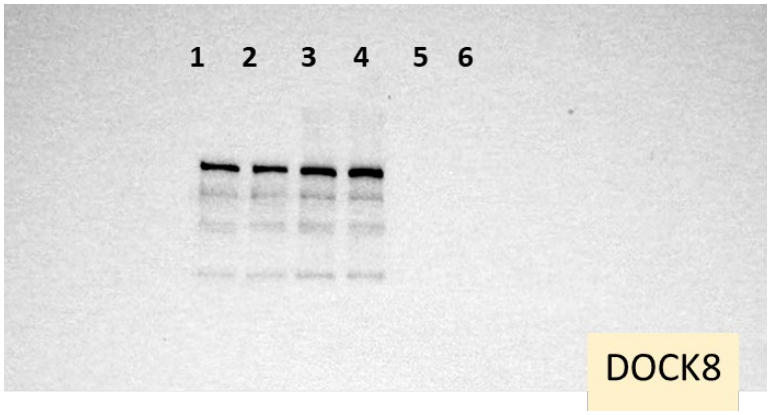

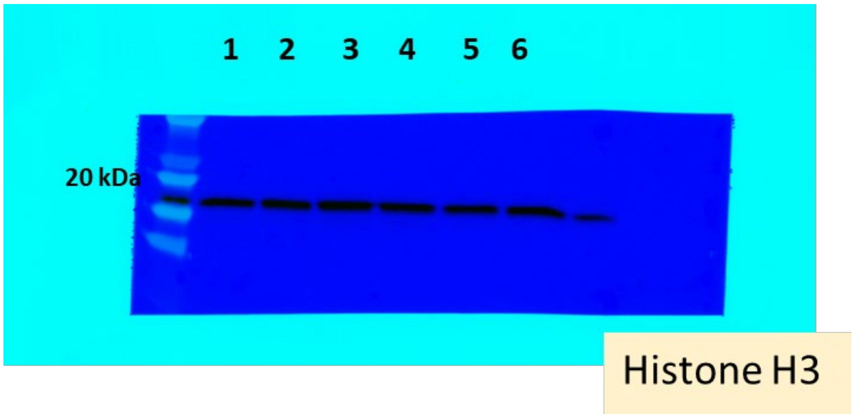
1 2 3 4 5 6
20 kDa
Histone H3

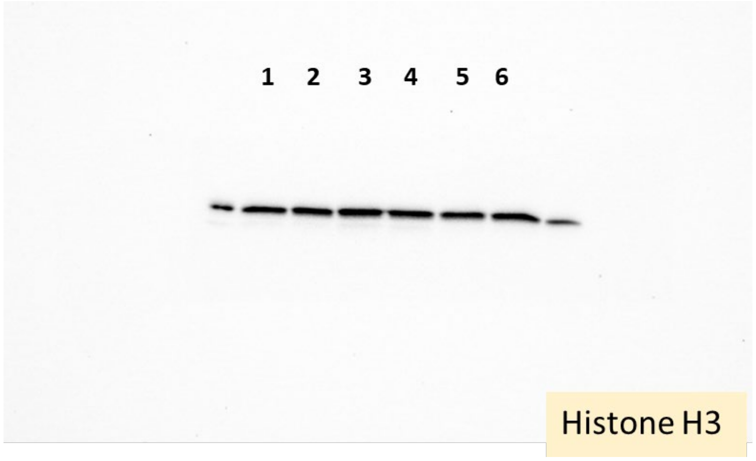
1 2 3 4 5 6
Histone H3

Replicate 2

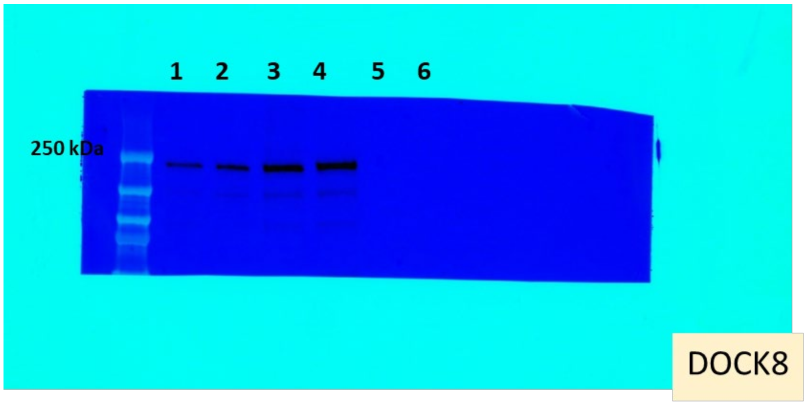
1 2 3 4 5 6
250 kDa
DOCK8

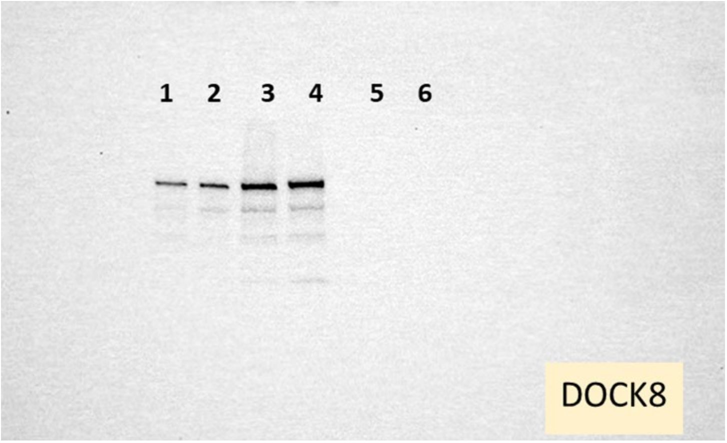
1 2 3 4 5 6
DOCK8

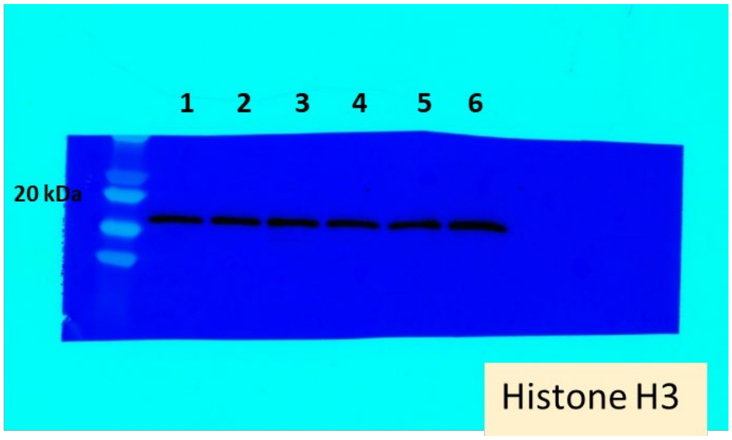
1 2 3 4 5 6
20 kDa
Histone H3

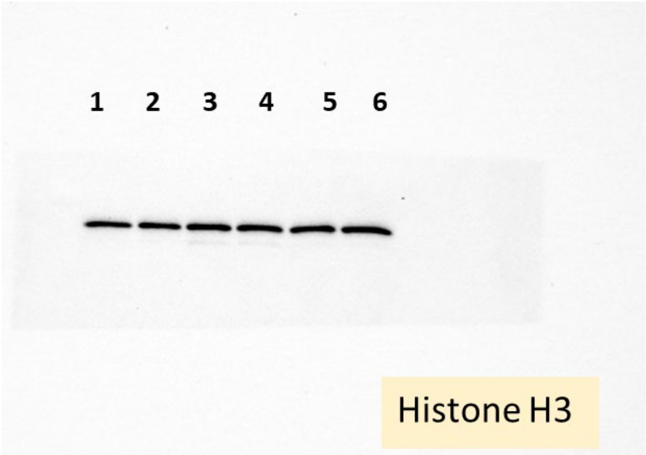
1 2 3 4 5 6
Histone H3

Replicate 3

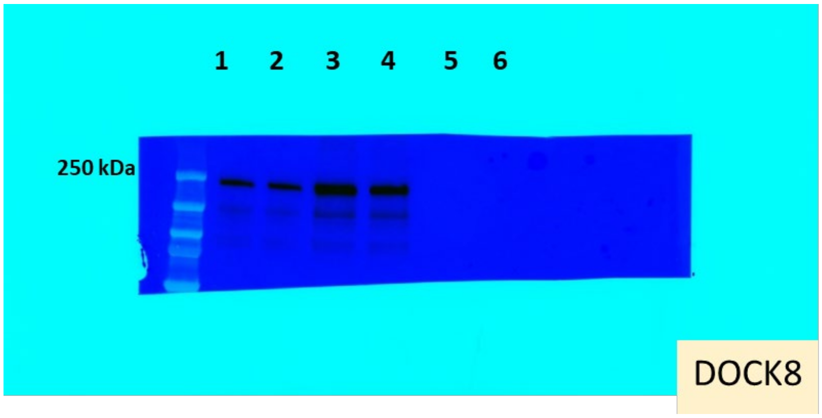

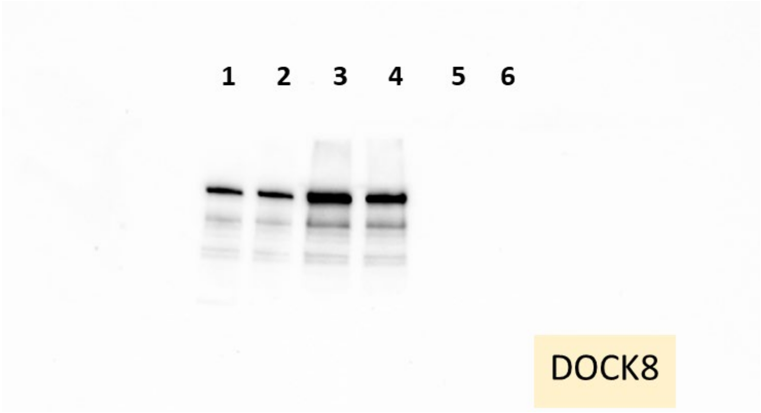

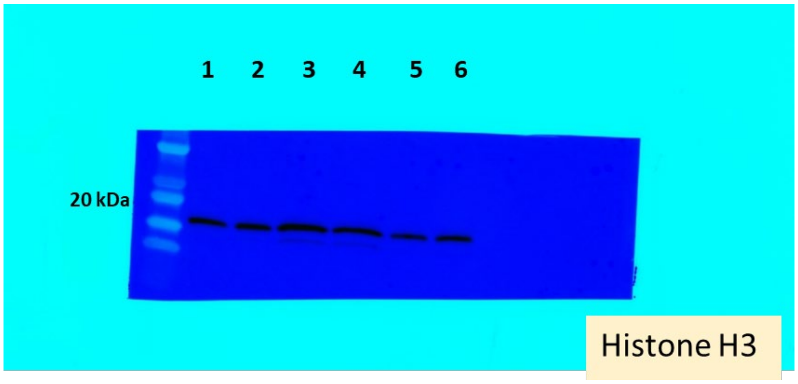

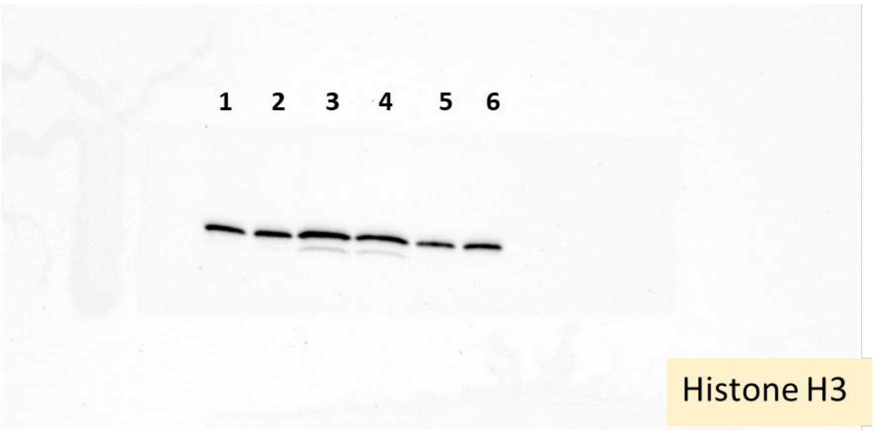

Supplementary Figure S6

1: Non-targeting siRNA_untreated

2: Non-targeting siRNA_sTn

3: Non-targeting siRNA_antibody + sTn

4: SHP-2 siRNA_untreated

5: SHP-2 siRNA_sTn

6: SHP-2 siRNA_ antibody + sTn

Replicate 1

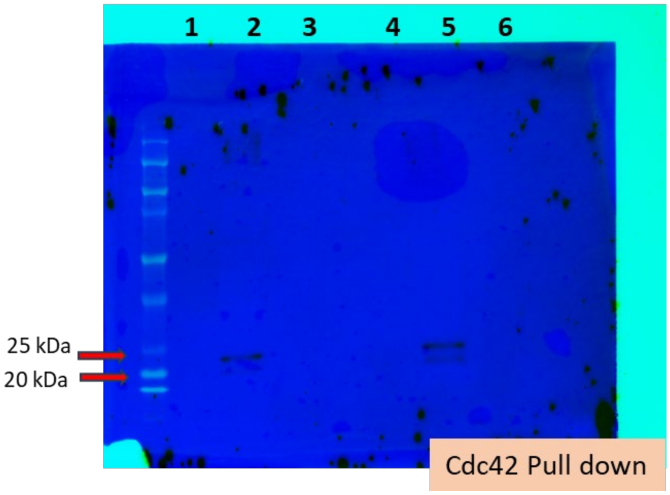
1
2
3
4
5
6
25 kDa
20 kDa
Cdc42 Pull down

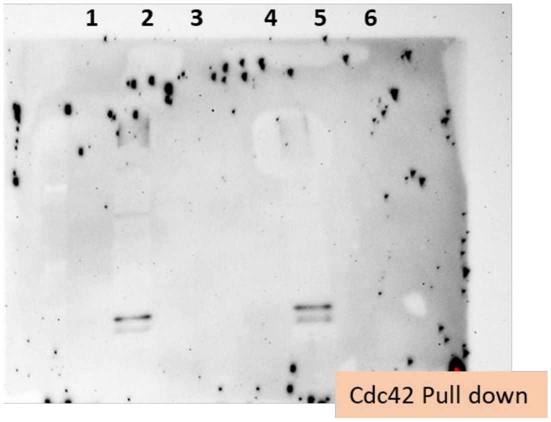
1
2
3
4
5
6
Cdc42 Pull down

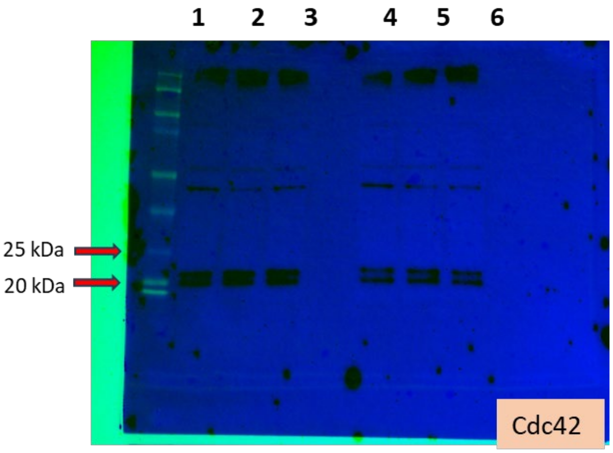
1
2
3
4
5
6
25 kDa
20 kDa
Cdc42

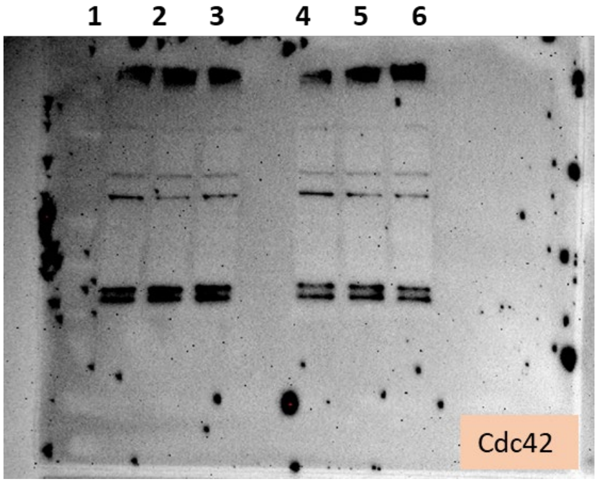
1 2 3 4 5 6
Cdc42

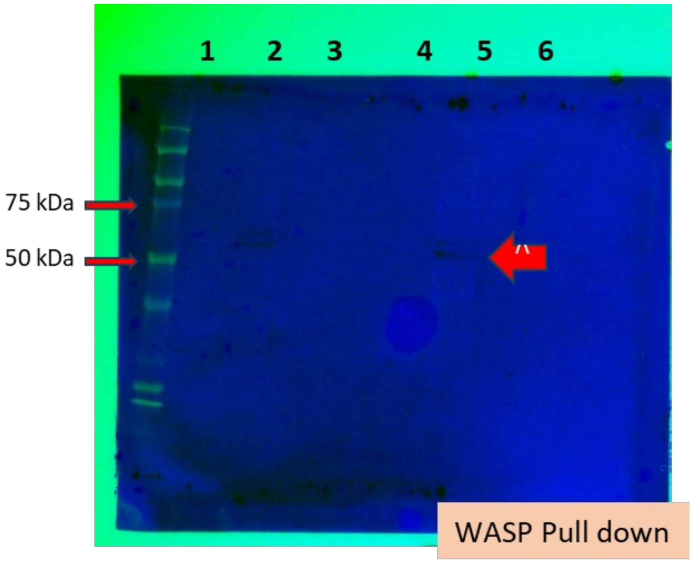
1 2 3 4 5 6
75 kDa
50 kDa
WASP Pull down

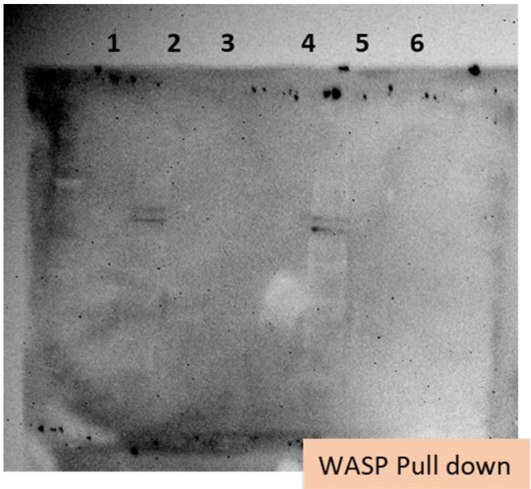
1 2 3 4 5 6
WASP Pull down

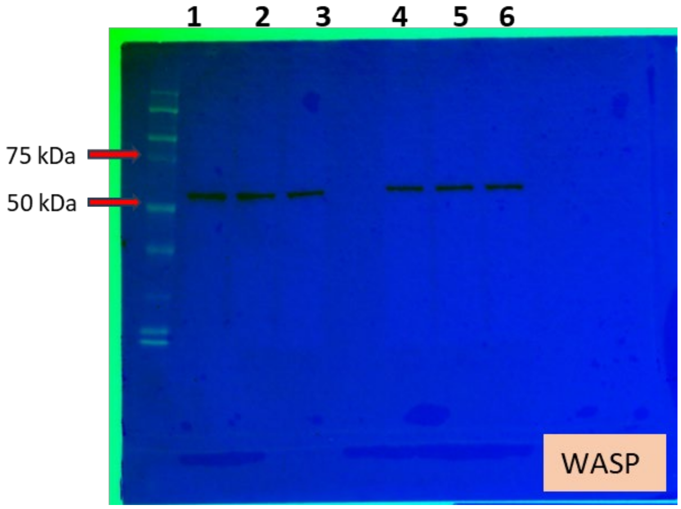
1 2 3 4 5 6
75 kDa
50 kDa
WASP

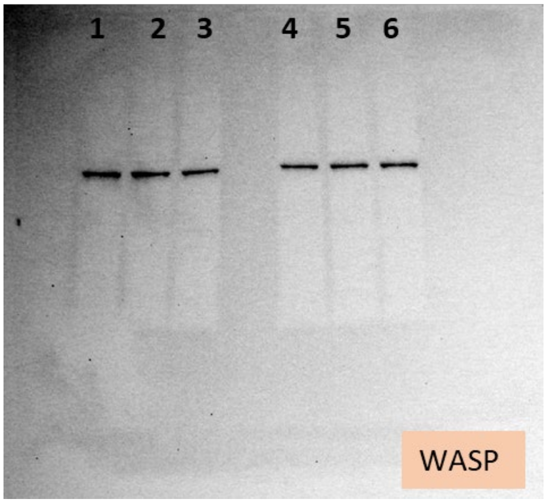
1 2 3 4 5 6
WASP

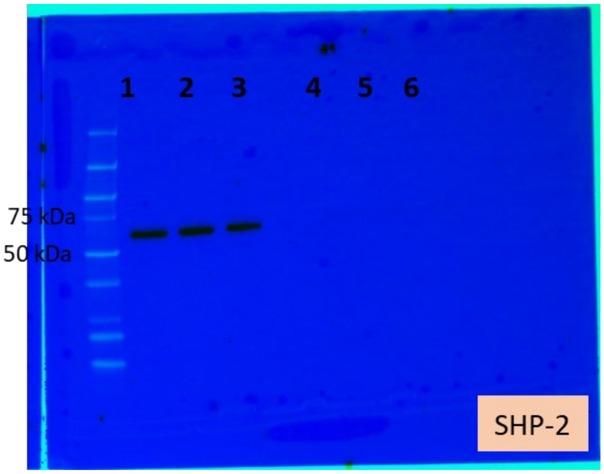
1 2 3 4 5 6
75 kDa
50 kDa
SHP-2

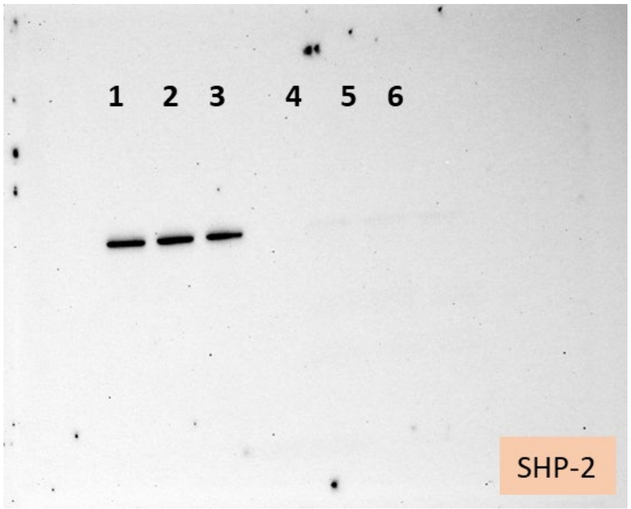
1 2 3 4 5 6
SHP-2

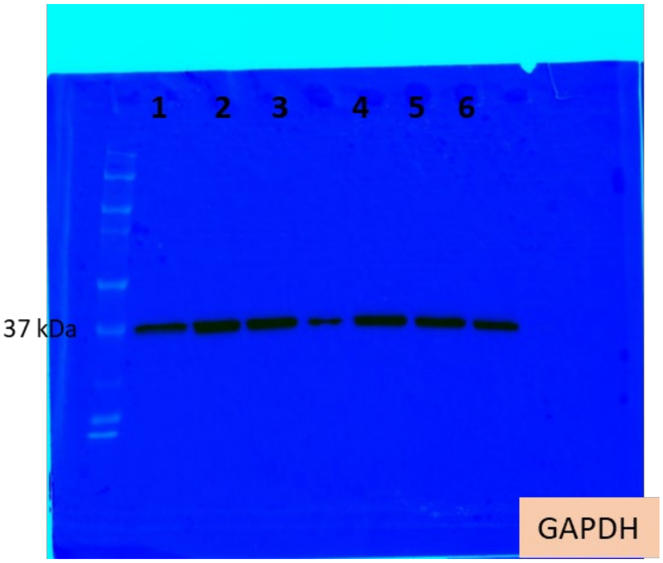
1 2 3 4 5 6
37 kDa
GAPDH

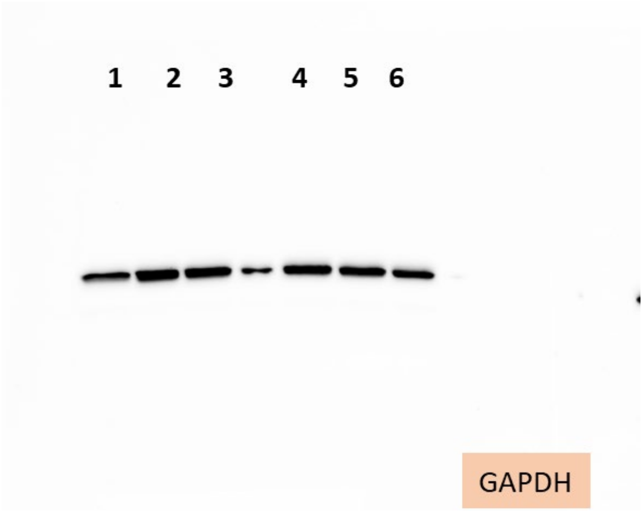
1 2 3 4 5 6
GAPDH

Replicate 2

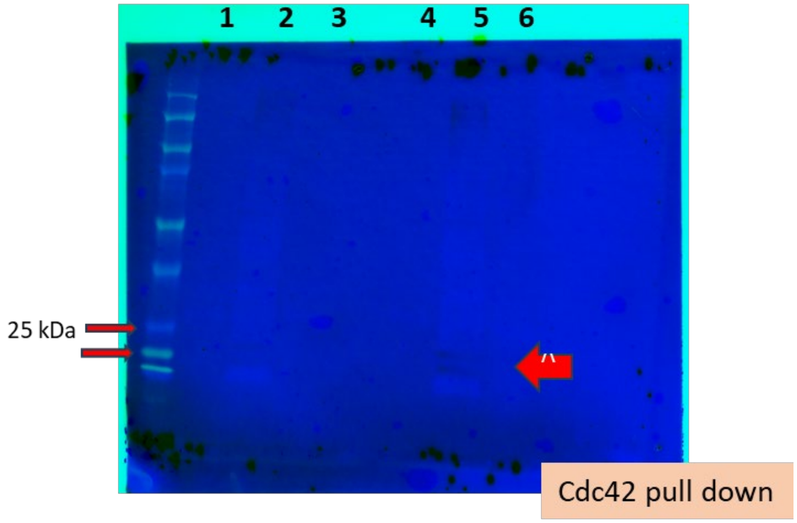
1
2
3
4
5
6
25 kDa
^
Cdc42 pull down

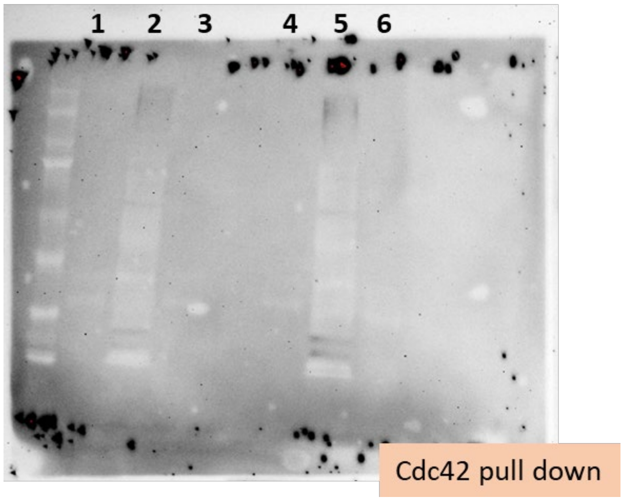
1
2
3
4
5
6
Cdc42 pull down

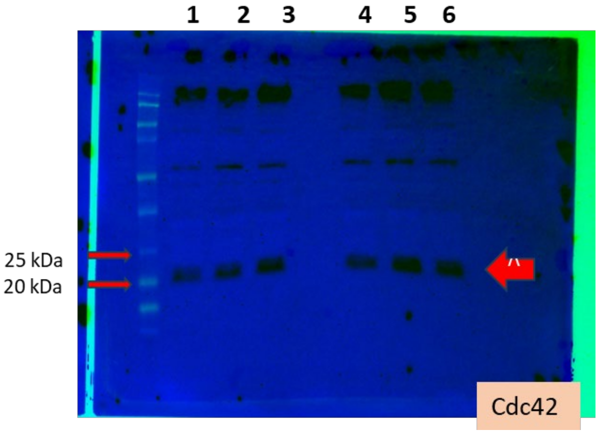
1
2
3
4
5
6
25 kDa
20 kDa
^
Cdc42

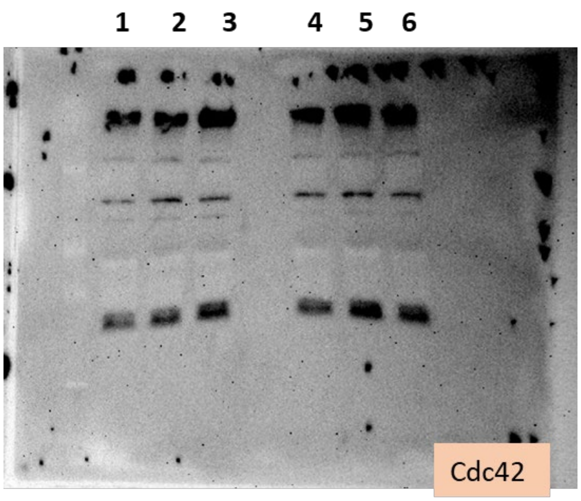
1 2 3 4 5 6
Cdc42

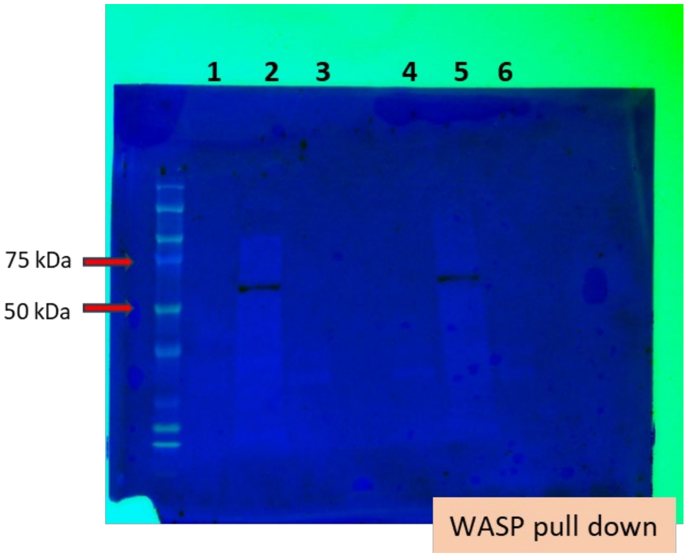
1 2 3 4 5 6
75 kDa
50 kDa
WASP pull down

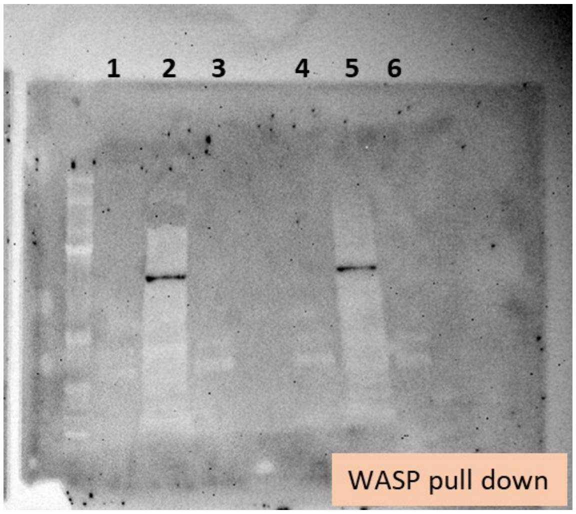
1 2 3 4 5 6
WASP pull down

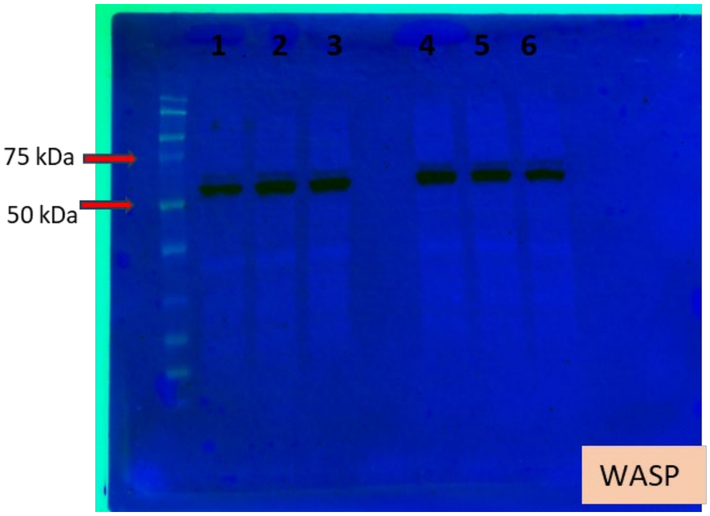
1 2 3 4 5 6
75 kDa
50 kDa
WASP

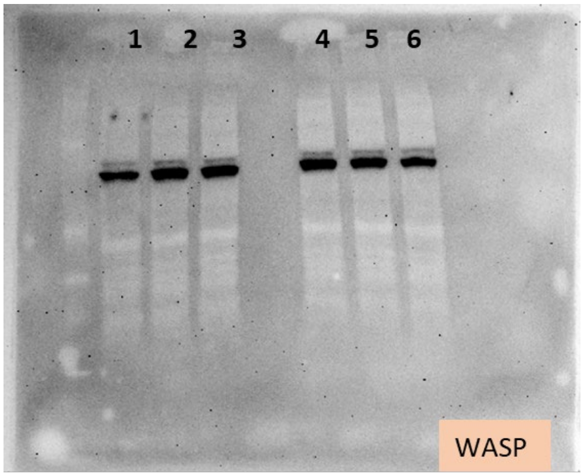
1 2 3 4 5 6
WASP

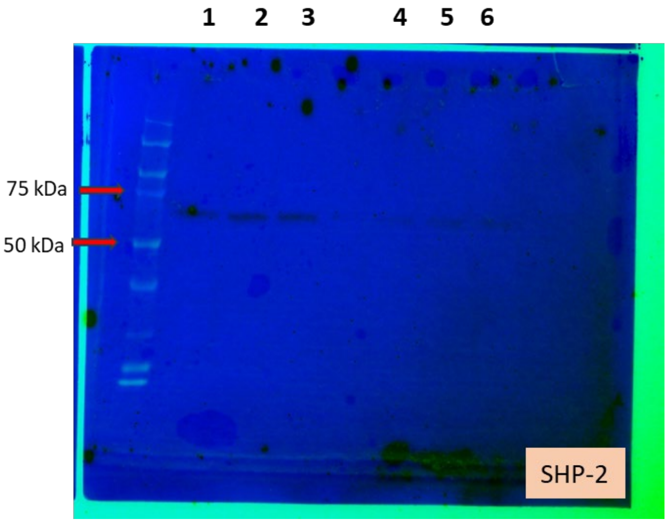
1 2 3 4 5 6
75 kDa
50 kDa
SHP-2

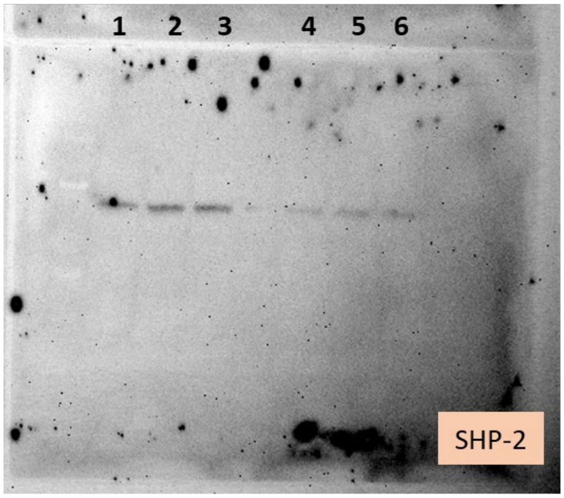
1 2 3 4 5 6
SHP-2

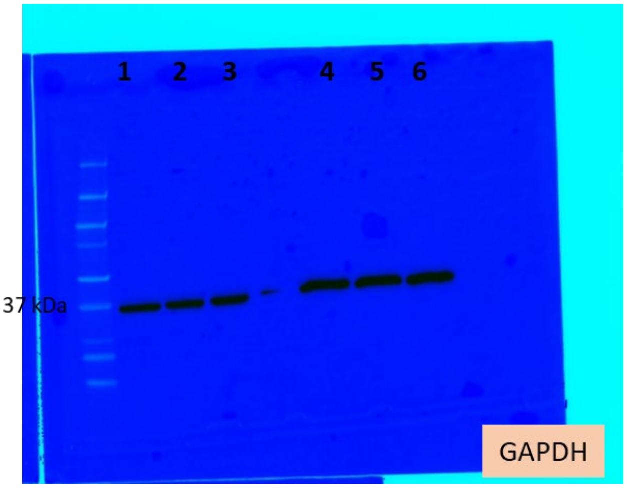
1 2 3 4 5 6
37 kDa
GAPDH

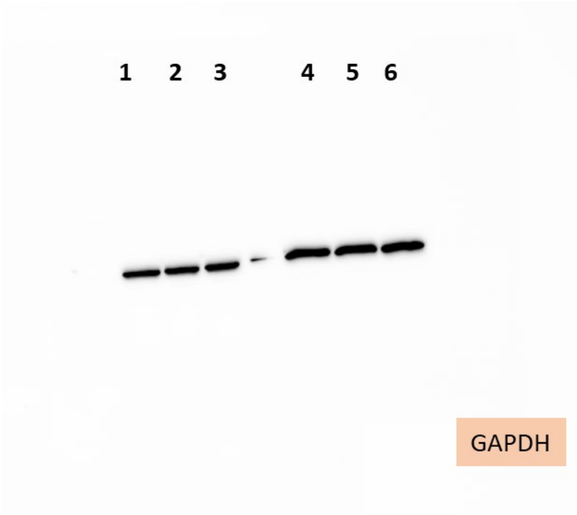
1 2 3 4 5 6
GAPDH

Replicate 3

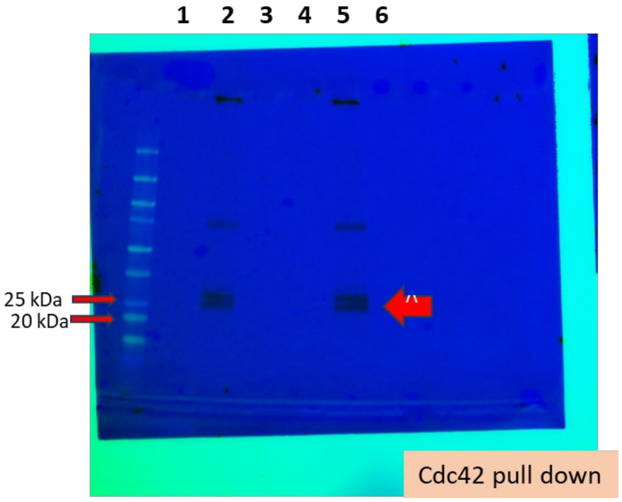
1 2 3 4 5 6
25 kDa
20 kDa
Cdc42 pull down

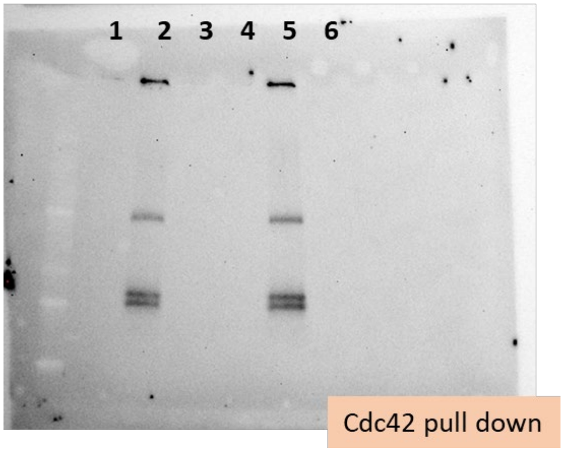
1 2 3 4 5 6
Cdc42 pull down

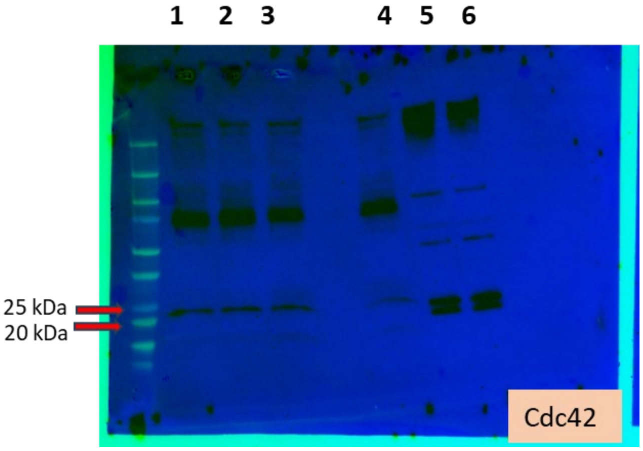
1 2 3 4 5 6
25 kDa
20 kDa
Cdc42

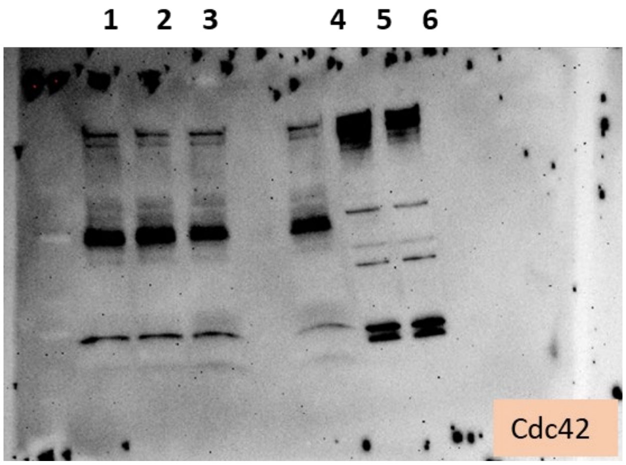
1 2 3 4 5 6
Cdc42

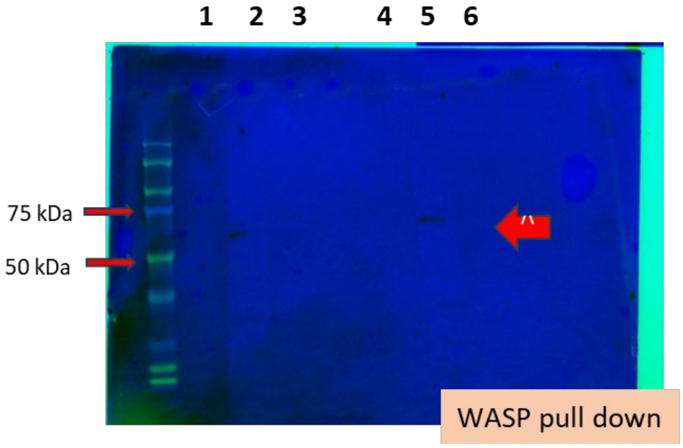
1 2 3 4 5 6
75 kDa
50 kDa
WASP pull down

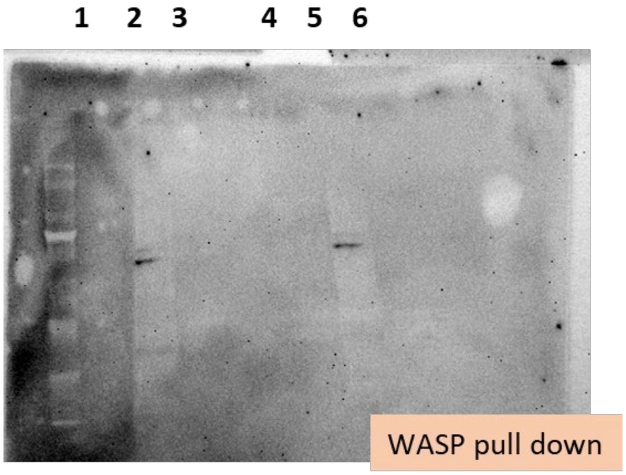
1 2 3 4 5 6
WASP pull down

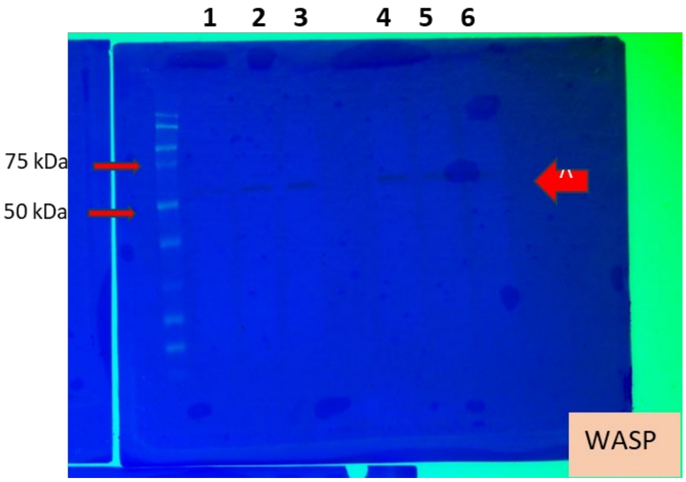
1 2 3 4 5 6
75 kDa
50 kDa
WASP

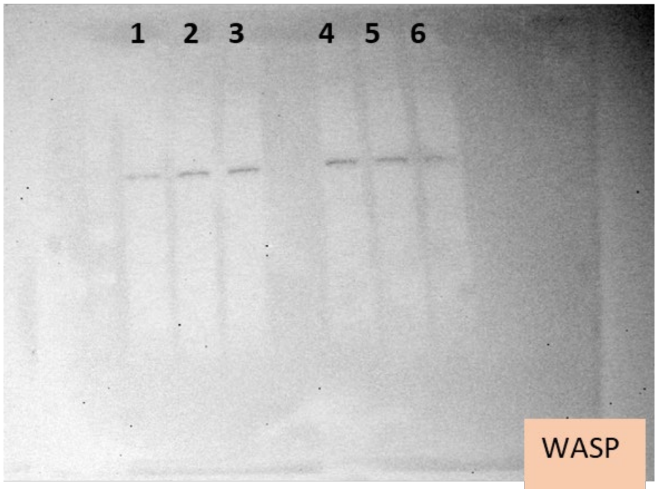
1 2 3 4 5 6
WASP

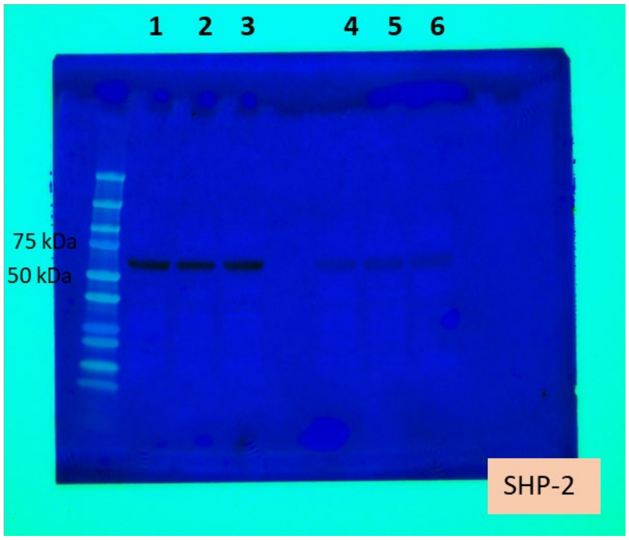
1 2 3 4 5 6
75 kDa
50 kDa
SHP-2

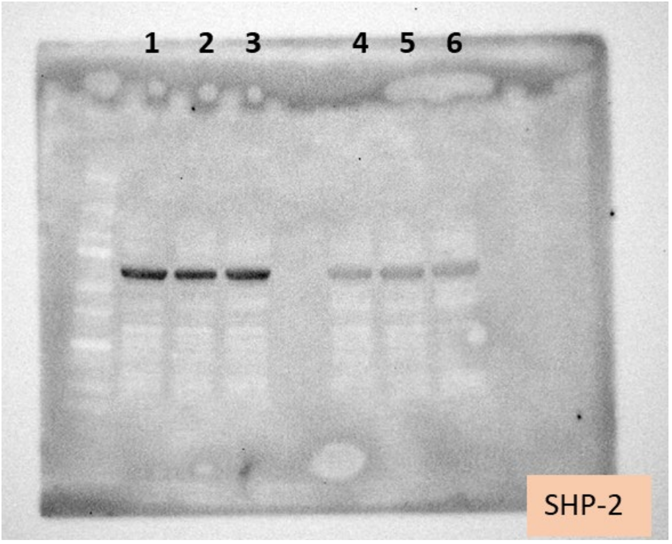
1 2 3 4 5 6
SHP-2

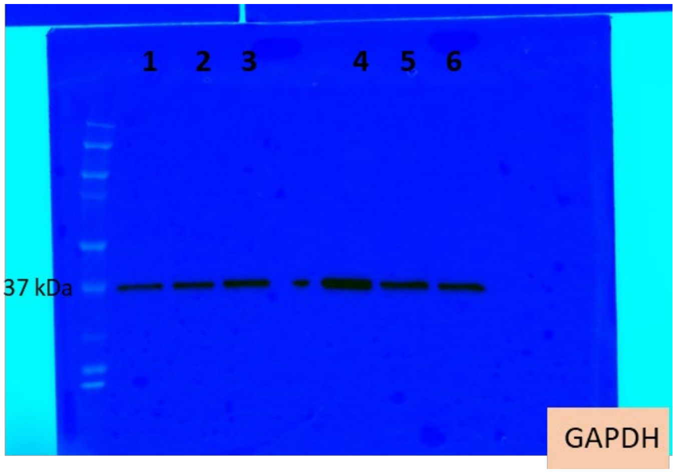
1 2 3 4 5 6
37 kDa
GAPDH

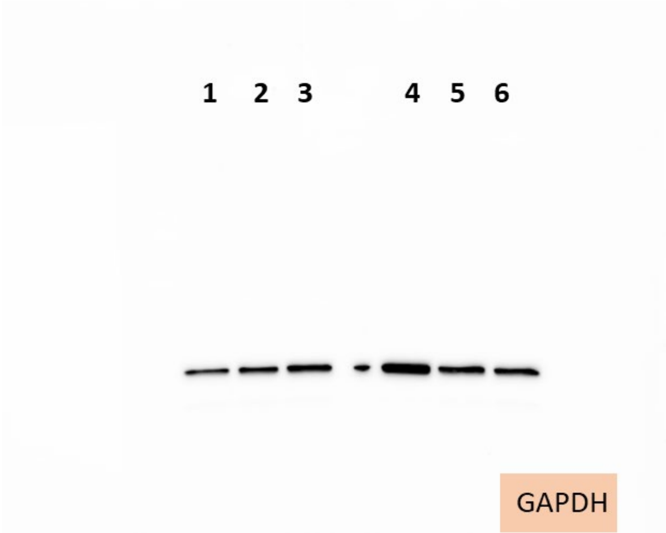
1 2 3 4 5 6
GAPDH
