# Supplementary material for: Siglec-6 as a therapeutic target for cell migration and adhesion in chronic lymphocytic leukemia
Source: Nat Commun. 2024 Jun 18;15:5180. doi: 10.1038/s41467-024-48678-3 (PMC11189495; doi:10.1038/s41467-024-48678-3)
Supplement: Supplementary file 2 — Reporting Summary [file 41467_2024_48678_MOESM2_ESM.pdf]

nature portfolio

Corresponding author(s): Natarajan Muthusamy, PhD

Last updated by author(s): Dec 18, 2023

Reporting Summary

Nature Portfolio wishes to improve the reproducibility of the work that we publish. This form provides structure for consistency and transparency in reporting. For further information on Nature Portfolio policies, see our Editorial Policies and the Editorial Policy Checklist.

Please do not complete any field with "not applicable" or n/a. Refer to the help text for what text to use if an item is not relevant to your study.
For final submission: please carefully check your responses for accuracy; you will not be able to make changes later.

Statistics

For all statistical analyses, confirm that the following items are present in the figure legend, table legend, main text, or Methods section.

n/a	Confirmed	
☐	☒	The exact sample size (*n*) for each experimental group/condition, given as a discrete number and unit of measurement
☐	☒	A statement on whether measurements were taken from distinct samples or whether the same sample was measured repeatedly
☐	☒	The statistical test(s) used AND whether they are one- or two-sided<br>*Only common tests should be described solely by name; describe more complex techniques in the Methods section.*
☐	☒	A description of all covariates tested
☐	☒	A description of any assumptions or corrections, such as tests of normality and adjustment for multiple comparisons
☐	☒	A full description of the statistical parameters including central tendency (e.g. means) or other basic estimates (e.g. regression coefficient) AND variation (e.g. standard deviation) or associated estimates of uncertainty (e.g. confidence intervals)
☐	☒	For null hypothesis testing, the test statistic (e.g. *F*, *t*, *r*) with confidence intervals, effect sizes, degrees of freedom and *P* value noted<br>*Give P values as exact values whenever suitable.*
☒	☐	For Bayesian analysis, information on the choice of priors and Markov chain Monte Carlo settings
☐	☒	For hierarchical and complex designs, identification of the appropriate level for tests and full reporting of outcomes
☒	☐	Estimates of effect sizes (e.g. Cohen's *d*, Pearson's *r*), indicating how they were calculated

Our web collection on statistics for biologists contains articles on many of the points above.

Software and code

Policy information about availability of computer code

Data collection	Graph pad Prismv9.2.0, Kaluza v2.0
Data analysis	Mass spectrometry database searching - All MS/MS samples were analyzed using Mascot (Matrix Science, London, UK; version 2.6.2). Mascot was set up to search the UNIPROT_HUMAN_Reviewed_20181126 database (49259 entries) assuming the digestion enzyme stricttrypsin. Mascot was searched with a fragment ion mass tolerance of 0.50 Da and a parent ion tolerance of 5.0 PPM. Carbamidomethyl of cysteine was specified in Mascot as a fixed modification. Deamidated of asparagine and glutamine and oxidation of methionine were specified in Mascot as variable modifications.<br>Criteria For Protein Identification- Scaffold (version Scaffold_4.11.0, Proteome Software Inc., Portland, OR) was used to validate MS/MS based peptide and protein identifications. Peptide identifications were accepted if they could be established at greater than 91.0% probability to achieve an FDR less than 1.0% by the Peptide Prophet algorithm (Keller, A et al Anal. Chem. 2002;74(20):5383-92) with Scaffold delta-mass correction. Protein identifications were accepted if they could be established at greater than 99.0% probability to achieve an FDR less than 1.0% and contained at least 2 identified peptides. Protein probabilities were assigned by the Protein Prophet algorithm (Nesvizhskii, Al et al Anal. Chem. 2003;75(17):4646-58). Proteins that contained similar peptides and could not be differentiated based on MS/MS analysis alone were grouped to satisfy the principles of parsimony.<br>For quantitation, a two tailed student t test was performed for these two group. Proteins with P value less than 0.05 (or 5%) were considered to be significantly changed. Proteins with a fold change >2 or <0.5 were considered as up- and down-regulation.<br>Statistical analysis: All analyses were performed by the OSU Center for Biostatistics using previously described models using SAS/STAT software, v9.4 of the SAS System for Windows (SAS Institute Inc., Cary, NC).<br>This proteomics dataset was generated for this study.

For manuscripts utilizing custom algorithms or software that are central to the research but not yet described in published literature, software must be made available to editors and reviewers. We strongly encourage code deposition in a community repository (e.g. GitHub). See the Nature Portfolio guidelines for submitting code & software for further information.

Data

Policy information about availability of data

All manuscripts must include a data availability statement. This statement should provide the following information, where applicable:

- Accession codes, unique identifiers, or web links for publicly available datasets
- A description of any restrictions on data availability

The authors declare that the data supporting the findings of this study are available within the paper and its supplementary information files. All the raw data generated in this study are provided in the Source Data file. The mass spectrometry publicly available data used in this study have been deposited in the ProteomeXchange database (Project accession: PXD039892). The complete list of proteins identified in the mass spectrometry experiment is provided in the Supplementary Information. Other relevant data that further support the findings of this study are available from the corresponding author upon request. Source data are provided with this paper.

Research involving human participants, their data, or biological material

Policy information about studies with human participants or human data. See also policy information about sex, gender (identity/presentation), and sexual orientation and race, ethnicity and racism.

Reporting on sex and gender	All the CLL and healthy donor samples were de-identified and as a result, information regarding sex and age was not available.
Reporting on race, ethnicity, or other socially relevant groupings	All the CLL and healthy donor samples were de-identified and as a result, information regarding race and ethnicity was not available.
Population characteristics	All patients examined had CLL which was defined by the 2008 IWCLL criteria. Covariate-relevant characteristics collected include: IGHV mutation status (un-mutated/mutated), 17p deletion (yes/no), 13q deletion (yes/no), previous treatment (yes/no).
Recruitment	Peripheral blood or lymph node biopsy from CLL patients was obtained after written informed consent in accordance with the Declaration of Helsinki, under a protocol approved by the institutional review board (IRB) at The Ohio State University. Participants were not prospectively identified for the current study. The population chosen is representative for a tertiary referral center without any identifiable bias.
Ethics oversight	Protocol was approved by the Ohio State University

Note that full information on the approval of the study protocol must also be provided in the manuscript.

Field-specific reporting

Please select the one below that is the best fit for your research. If you are not sure, read the appropriate sections before making your selection.

[X] Life sciences [ ] Behavioural & social sciences [ ] Ecological, evolutionary & environmental sciences

For a reference copy of the document with all sections, see nature.com/documents/nr-reporting-summary-flat.pdf

Life sciences study design

All studies must disclose on these points even when the disclosure is negative.

Sample size	No statistical tests were done to pre-determine samples sizes. Based on previous publications from our group and upon recommendation from Dr. Xiaokui Mo at the OSU Center for Biostatistics, a sample size of n ≥ 3 for experiments with cell lines and n ≥ 5 for experiments with primary cells was chosen and was sufficient to detect a response between groups. For all mouse experiments, 4-9 mice were used/group to allow sufficient power analysis.
Data exclusions	No datapoints were excluded from any of the experiments.

Replication

All replication attempts to verify experimental findings, including engraftment and therapeutic studies in the *in vivo* experiments, were successful.

Randomization

In engraftment studies with immunocompetent huCD3 mice, mice were randomly assigned to treatment groups according to the date on which their circulating leukemia exceeded the 5 % threshold and evenly distributed according to disease severity at that date. For the *in vivo* migration experiment, NSG mice were randomly assigned to each group and there were no covariates since they were genetically similar. For the CLL PDX model, 2 mice received one CLL patient donor engraftment (with a total of 6 mice and 3 patients) and were then randomly assigned into control or treated group.

Blinding

For the proteomics analysis, the investigator performing the analysis was blinded to the isotype versus Siglec-6 antibody pull down group. For statistical analysis, the biostatistician was blinded to group allocation. For all therapeutic and other mouse experiments, veterinary mouse technicians determining euthanasia criteria were blinded to the treatment enrollment group.

Reporting for specific materials, systems and methods

We require information from authors about some types of materials, experimental systems and methods used in many studies. Here, indicate whether each material, system or method listed is relevant to your study. If you are not sure if a list item applies to your research, read the appropriate section before selecting a response.

Materials & experimental systems

n/a	Involved in the study
☐	☒ Antibodies
☐	☒ Eukaryotic cell lines
☒	☐ Palaeontology and archaeology
☐	☒ Animals and other organisms
☒	☐ Clinical data
☒	☐ Dual use research of concern
☒	☐ Plants

Methods

n/a	Involved in the study
☒	☐ ChIP-seq
☐	☒ Flow cytometry
☒	☐ MRI-based neuroimaging

Antibodies

Antibodies used

Antibodies used for immunoblotting:
1) GAPDH (1:600000, Millipore Sigma, #G5262)
2) WASP (1:1000, Cell Signaling Technology, #4860)
3) Cdc42 (1:1000, Cell Signaling Technology, #2462)
4) AIF (1:1000, Cell Signaling Technology, #5318)
5) Histone H3 (1:1000, Cell Signaling Technology, #4499)
6) Siglec-6 (1:1000, Abcam, #ab262851)
7) DOCK8 (1:3000, Abcam, #ab175208)
8) Sialyl Tn (1:100, LifeSpan Biosciences, #LS-C170901-0.5)
9) Anti-mouse IgG HRP (1:3000, Biorad, #1706516)
10) Anti-Rabbit IgG HRP (1:3000, Cell Signaling Technology, #7074)

Antibodies used for immunofluorescence:
1) Siglec-6 (1:500, Sigma Aldrich, #HPA009084)
2) DOCK8 (1:100, Origene, #TA506484)
3) Anti-rabbit IgG Alexa fluor 488 (1:1000, Invitrogen, #A-11008)
4) Anti-Mouse IgG Alexa fluor 594 (1:1000, Invitrogen, #A-11005)

Antibody used for immunoprecipitation:
1) Siglec-6 (1:50, LifeSpan Biosciences, #LS-C196855)

Antibodies used for flow cytometry:
1) BV510 anti-human CD5 (1:30, BD Biosciences, #563380)
2) BV421 anti-human CD3 (1:30, BD Biosciences, #562426)
3) BV786 anti-human CD19 (1:30, BD Biosciences, #563325)
4) PECF594 anti-human CD73 (1:30, BD Biosciences, #562817)
5) PerCP-Cy5.5 anti-human CD90 (1:30, BD Biosciences, #561557)
6) BV421 anti-human CD34 (1:30, BD Biosciences, #562577)
7) APC anti-human CD45 (1:10, BD Biosciences, #555485)
8) FITC anti-human CD49d (1:30, Biolegend, #304316)
9) PerCP-Cy5.5 anti-human CXCR4 (1:30, Biolegend, #306516)
10) Unconjugated anti-human sialyl Tn (1:25, Abcam, ab115957)

11) PE-Cy7 Anti-mouse (1:25, ThermoFisher, #25-4015-82)- Secondary for Sialyl Tn antibody
12) PECF594 anti-mouse CD45 (1:30, BD Biosciences, #562420)
13) APC-Cy7 anti-mouse CD45 (1:30, Biolegend, #103116)
14) FITC anti-mouse CD19 (1:30, Biolegend, #115506)
15) BUV395 anti-human CD3 (1:30, BD Biosciences, #564000)
16) BUV737 anti-mouse CD5 (1:30, BD Biosciences, #612809)
17) APC-Cy7 anti-mouse CD3 (1:30, BD Biosciences, #557596)
18) PE anti-human Siglec-6 (1:10, R&D Systems, FAB2859)
19) Biotin anti-human Siglec-6 (1:100, Collaborator)

Validation

Flow cytometry gating strategies were followed according to published data and technical resource publications. They were adapted to allow exclusion and interrogation of CD19+CD5+ CLL-like populations. Fluorescence-minus-one or isotype controls were used for gate positions.

All antibodies used for western blotting, immunofluorescence, and flow cytometry are from commercial sources (Abcam, Millipore Sigma, Cell Signaling Technology, BD Biosciences, Invitrogen, Origene, LifeSpan Bioseceinces). Validation data are available on the manufacturer's website and datasheets:

1) GAPDH (Millipore Sigma, #G5262) -> https://www.sigmaaldrich.com/US/en/product/sigma/g5262
2) WASP (Cell Signaling Technology, #4860) -> https://www.cellsignal.com/products/primary-antibodies/wasp-antibody/4860
3) Cdc42 (Cell Signaling Technology, #2462) -> https://www.cellsignal.com/products/primary-antibodies/cdc42-antibody/2462
4) AIF (1:1000, Cell Signaling Technology, #5318) -> https://www.cellsignal.com/products/primary-antibodies/aif-d39d2-xp-rabbit-mab/5318
5) Histone H3 (Cell Signaling Technology, #4499) -> https://www.cellsignal.com/products/primary-antibodies/histone-h3-d1h2-xp-rabbit-mab/4499
6) Siglec-6 (Abcam, #ab262851) -> https://www.abcam.com/products/primary-antibodies/siglec6-antibody-ab262851.html
7) DOCK8 (Abcam, #ab175208) -> https://www.abcam.com/products/primary-antibodies/dock8-antibody-epr12511-ab175208.html
8) Sialyl Tn (LifeSpan Biosciences, #LS-C170901-0.5) -> https://www.lsbio.com/antibodies/sialyl-tn-antibody-clone-b35.1-elisa-ihc-wb-western-ls-c170901/178302
9) Anti-mouse IgG HRP (Biorad, #1706516) -> https://www.bio-rad.com/en-us/sku/1706516-goat-anti-mouse-igg-h-l-hrp-conjugate?ID=1706516
10) Anti-Rabbit IgG HRP (Cell Signaling Technology, #7074) -> https://www.cellsignal.com/products/secondary-antibodies/anti-rabbit-igg-hrp-linked-antibody/7074
11) Siglec-6 (Sigma Aldrich, #HPA009084) -> https://www.sigmaaldrich.com/US/en/product/sigma/hpa009084
12) DOCK8 (Origene, #TA506484) -> https://www.origene.com/catalog/antibodies/primary-antibodies/ta506484/dock8-mouse-monoclonal-antibody-clone-id-oti2c11
13) Anti-rabbit IgG Alexa fluor 488 (Invitrogen, #A-11008) -> https://www.thermofisher.com/antibody/product/Goat-anti-Rabbit-IgG-H-L-Cross-Adsorbed-Secondary-Antibody-Polyclonal/A-11008
14) Anti-Mouse IgG Alexa fluor 594 Invitrogen, #A-11005) -> https://www.thermofisher.com/antibody/product/Goat-anti-Mouse-IgG-H-L-Cross-Adsorbed-Secondary-Antibody-Polyclonal/A-11005
15) Siglec-6 (LifeSpan Biosciences, #LS-C196855) -> https://www.lsbio.com/antibodies/siglec6-antibody-clone-2g6-elisa-ip-wb-western-ls-c196855/204911
16) BV510 anti-human CD5 (BD Biosciences, #563380) -> https://www.bdbiosciences.com/en-us/products/reagents/flow-cytometry-reagents/research-reagents/single-color-antibodies-ruo/bv510-mouse-anti-human-cd5.563380
17) BV421 anti-human CD3 (BD Biosciences, #562426) -> https://www.bdbiosciences.com/en-us/products/reagents/microscopy-imaging-reagents/immunofluorescence-reagents/bv421-mouse-anti-human-cd3.562426
18) BV786 anti-human CD19 (BD Biosciences, #563325) -> https://www.bdbiosciences.com/en-us/products/reagents/flow-cytometry-reagents/research-reagents/single-color-antibodies-ruo/bv786-mouse-anti-human-cd19.563325
19) PECF594 anti-human CD73 (BD Biosciences, #562817) -> https://www.bdbiosciences.com/en-us/products/reagents/flow-cytometry-reagents/research-reagents/single-color-antibodies-ruo/pe-cf594-mouse-anti-human-cd73.562817
20) PerCP-Cy5.5 anti-human CD90 (BD Biosciences, #561557) -> https://www.bdbiosciences.com/en-us/products/reagents/flow-cytometry-reagents/research-reagents/single-color-antibodies-ruo/percp-cy-5-5-mouse-anti-human-cd90.561557
21) BV421 anti-human CD34 (BD Biosciences, #562577) -> https://www.bdbiosciences.com/en-us/products/reagents/flow-cytometry-reagents/research-reagents/single-color-antibodies-ruo/bv421-mouse-anti-human-cd34.562577

22) APC anti-human CD45 (BD Biosciences, #555485) -> https://www.bdbiosciences.com/en-us/products/reagents/flow-cytometry-reagents/research-reagents/single-color-antibodies-ruo/apc-mouse-anti-human-cd45.555485
23) FITC anti-human CD49d (Biolegend, #304316) -> https://www.biolegend.com/en-us/products/fitc-anti-human-cd49d-antibody-8325
24) PerCP-Cy5.5 anti-human CXCR4 (Biolegend, #306516) -> https://www.biolegend.com/en-us/products/percp-cyanine5-5-anti-human-cd184-cxcr4-antibody-6777
25) Unconjugated anti-human sialyl Tn (Abcam, ab115957) -> https://www.abcam.com/products/primary-antibodies/sialyl-tn-antibody-stn-219-ab115957.html
26) PE-Cy7 Anti-mouse (ThermoFisher, #25-4015-82)- Secondary for Sialyl Tn antibody -> https://www.thermofisher.com/antibody/product/Rat-anti-Mouse-IgG1-Secondary-Antibody-clone-M1-14D12-Monoclonal/25-4015-82
27) PECF594 anti-mouse CD45 (BD Biosciences, #562420) -> https://www.bdbiosciences.com/en-us/products/reagents/flow-cytometry-reagents/research-reagents/single-color-antibodies-ruo/pe-cf594-rat-anti-mouse-cd45.562420
28) APC-Cy7 anti-mouse CD45 (Biolegend, #103116) -> https://www.biolegend.com/en-us/products/apc-cyanine7-anti-mouse-cd45-antibody-2530
29) FITC anti-mouse CD19 (Biolegend, #115506) -> https://www.biolegend.com/en-us/products/fitc-anti-mouse-cd19-antibody-1528
30) BUV395 anti-human CD3 (BD Biosciences, #564000) -> https://www.bdbiosciences.com/en-us/products/reagents/flow-cytometry-reagents/research-reagents/single-color-antibodies-ruo/buv395-mouse-anti-human-cd3.564000
31) BUV737 anti-mouse CD5 (BD Biosciences, #612809) -> https://www.bdbiosciences.com/en-us/products/reagents/flow-cytometry-reagents/research-reagents/single-color-antibodies-ruo/buv737-rat-anti-mouse-cd5.612809
32) APC-Cy7 anti-mouse CD3 (BD Biosciences, #557596) -> https://www.bdbiosciences.com/en-us/products/reagents/flow-cytometry-reagents/research-reagents/single-color-antibodies-ruo/apc-cy-7-hamster-anti-mouse-cd3e.557596
33) PE anti-human Siglec-6 (R&D Systems, FAB2859) -> https://www.rndsystems.com/products/human-siglec-6-cd327-pe-conjugated-antibody-767329_fab2859p

Eukaryotic cell lines

Policy information about cell lines and Sex and Gender in Research

Cell line source(s)

MEC1 cell line was obtained from DSMZ (ACC 497) and DT-40 cell line was obtained from ATCC (CRL-2111). The MEC1-002 cell line was derived from the Siglec-6+ fraction of the MEC1 cell line by FACS sorting and was provided by Dr. Rader (University of Florida) under a Material Transfer Agreement (MTA) and Institutional Review Board (IRB) approval.

Authentication

MEC1 identity was reconfirmed via fluorescence in situ hybridization (FISH) using CLL probe panel, i.e. CEP12/13q14/13q34, ATM/p53, BCL6, MYC, IGH/CCND1 (Abbott Molecular, Des Plaines, IL), and 6q21 (Kreatech, Buffalo Grove, IL) (results for 200 analyzed cells were reported). All other cell lines were authenticated by short tandem repeat DNA profiling at The Ohio State University Genomic Services Core.

Mycoplasma contamination

All cell lines were confirmed to be mycoplasma negative using the MycoAlert™ Mycoplasma Detection Kit from Lonza (Rockland, ME) used within 3-4 weeks from thawing.

Commonly misidentified lines
(See ICLAC register)

none

Animals and other research organisms

Policy information about studies involving animals; ARRIVE guidelines recommended for reporting animal research, and Sex and Gender in Research

Laboratory animals

Human Siglec-6 transgenic mouse (hSiglec-6-Tg) was generated on a C57BL/6 background (Jackson laboratory, 000664) at the OSUCCC Transgenic Mouse Facility. Mice were housed in an environment with 12-hour light/12-hour dark cycle and temperatures were maintained at 75°F +/- 2°F with 30-60% humidity. Food and water were always accessible. Mice were grouped into cages and provided enrichment whenever possible.

	An equal ratio of 8-12 week male and female mice were maintained for experiments with huCD3 (B6.Cg-Tg (CD3E) 600Cpt/J) mice. For experiments with NSG mice (NOD.Cg-Prkdc^{scid} Il2rg^{tm1Wjl}/SzJ), 8-week male mice were used.
Wild animals	This study did not involve wild animals.
Reporting on sex	An equal ratio of 8-12 week male and female mice were maintained for experiments with huCD3 (B6.Cg-Tg (CD3E) 600Cpt/J) mice. For experiments with NSG mice (NOD.Cg-Prkdc^{scid} Il2rg^{tm1Wjl}/SzJ), 8-week male mice were used since they were more cost-effective.
Field-collected samples	This study did not involve field collected samples.
Ethics oversight	All experiments were carried out under protocols approved by The Ohio State University Institutional Animal Care and Use Committee. Pre-defined euthanasia criteria for mouse experiments included lethargy, impaired motility, splenomegaly, enlarged lymph nodes and/or superficial lymphoid tumors greater than 1.6 cm in diameter (or cumulative diameter of 1.6 cm if multiple tumors), decrease in body weight (>20%), development of tumor masses, ruffled fur, hunched back, failure to nest, and loss of appetite.

Note that full information on the approval of the study protocol must also be provided in the manuscript.

Clinical data

Policy information about clinical studies

All manuscripts should comply with the ICMJE guidelines for publication of clinical research and a completed CONSORT checklist must be included with all submissions.

Clinical trial registration	No clinical trials were included in this manuscript.
Study protocol	Patient samples used in this manuscript were obtained from stored samples in the Leukemia Tissue Bank (LTB) that were deposited under an IRB approved protocol. Samples were de-identified, and these patients were not recruited for clinical trials.
Data collection	No clinical trials were included in this manuscript.
Outcomes	No clinical trials were included in this manuscript.

Experiments of concern

Does the work involve any of these experiments of concern:

No	Yes	
X		Demonstrate how to render a vaccine ineffective
X		Confer resistance to therapeutically useful antibiotics or antiviral agents
X		Enhance the virulence of a pathogen or render a nonpathogen virulent
X		Increase transmissibility of a pathogen
X		Alter the host range of a pathogen
X		Enable evasion of diagnostic/detection modalities
X		Enable the weaponization of a biological agent or toxin
X		Any other potentially harmful combination of experiments and agents

Flow Cytometry

Plots

Confirm that:

- [X] The axis labels state the marker and fluorochrome used (e.g. CD4-FITC).
- [X] The axis scales are clearly visible. Include numbers along axes only for bottom left plot of group (a 'group' is an analysis of identical markers).
- [X] All plots are contour plots with outliers or pseudocolor plots.
- [X] A numerical value for number of cells or percentage (with statistics) is provided.

Methodology

Sample preparation	Samples were either cell lines, primary patient samples and healthy donors (B-CLL cells or bone marrow stromal cells). Single cell suspension from spleen was processed by physical mashing and RBC lysis. Single cell bone marrow suspension was prepared by flushing out femur, tibia and fibia bones using 30G syringes.
Instrument	LSRII Fortessa (BD Biosciences) was used for flow cytometry. Cell sorting was performed using a BD Bioscience BD FACSAria'M Fusion flow cytometer.
Software	Kaluza Analysis Software
Cell population abundance	For sorting of Siglec-6+ cells, purity was determined by re-running the sorted sample on the same machine. Sorted samples with >95% Siglec-6+ CD5+CD19+CD3- leukemic cells purity were used for in vivo studies
Gating strategy	Gating was first done in a FSC-A versus SSC-A plot to exclude debri and/or RBCs. A FSC-A versus FSC-H plot was then generated and gated on the cells from the FSC/SSC plot. Singlets were then selected. Live/dead staining was done and plotted against SSC-A followed by gating on singlets in order to select live cells.

- [X] Tick this box to confirm that a figure exemplifying the gating strategy is provided in the Supplementary Information.

This checklist template is licensed under a Creative Commons Attribution 4.0 International License, which permits use, sharing, adaptation, distribution and reproduction in any medium or format, as long as you give appropriate credit to the original author(s) and the source, provide a link to the Creative Commons license, and indicate if changes were made. The images or other third party material in this article are included in the article's Creative Commons license, unless indicated otherwise in a credit line to the material. If material is not included in the article's Creative Commons license and your intended use is not permitted by statutory regulation or exceeds the permitted use, you will need to obtain permission directly from the copyright holder. To view a copy of this license, visit http://creativecommons.org/licenses/by/4.0/
