# Supplementary material for: Siglec-6 as a therapeutic target for cell migration and adhesion in chronic lymphocytic leukemia
Source: Nat Commun. 2024 Jun 18;15:5180. doi: 10.1038/s41467-024-48678-3 (PMC11189495; doi:10.1038/s41467-024-48678-3)
Supplement: Supplementary file 3 — Description of Additional Supplementary Files [file 41467_2024_48678_MOESM3_ESM.pdf]

Supplementary Data 1: List of proteins identified from mass spectrometry analysis.

Protein lysates were prepared from MEC1-002 cells followed by pull down with anti-human Siglec-6 antibody or mouse IgG2b isotype control antibody conjugated to Dynabeads® Magnetic Beads. Proteins that interact with Siglec-6 were identified using Capillary-liquid chromatography-nanospray tandem mass spectrometry (Capillary-LC/MS/MS), performed on a Thermo Scientific orbitrap Fusion mass spectrometer.
